# Investigating the bioorthogonality of isocyanides

**DOI:** 10.1039/d5cc04136h

**Published:** 2025-12-15

**Authors:** Ryota Nakao, Viktor Savic, Jennifer A. Miles, Keith Livingstone, Marko D. Mihovilovic, Piotr Raubo, Stuart L. Warriner, Megan H. Wright

**Affiliations:** a School of Chemistry and Astbury Centre for Structural Molecular Biology, University of Leeds Leeds LS2 9JT UK S.L.Warriner@leeds.ac.uk M.H.Wright@leeds.ac.uk; b Institute of Applied Synthetic Chemistry, TU Wien Getreidemarkt 9/163-OC 1060 Vienna Austria; c Faculty of Biological Sciences and Astbury Centre for Structural Molecular Biology, University of Leeds Leeds LS2 9JT UK; d Research and Early Development, Oncology R&D, AstraZeneca Cambridge CB2 0AA UK

## Abstract

Isocyanides have been applied in bioorthogonal reactions as triggers for uncaging reactions. However, emerging evidence suggests that they may label proteins covalently. We synthesised a fluorophore-conjugated isocyanide and analysed its protein reactivity. Our results suggest that isocyanides cannot be considered to be bioorthogonal in chemical biology.

## Abstract 2

Isocyanides have been explored as bioorthogonal triggers for uncaging reactions. However, our findings show they can covalently label proteins, indicating they may not be fully bioorthogonal in biological systems.

## Graphical abstract

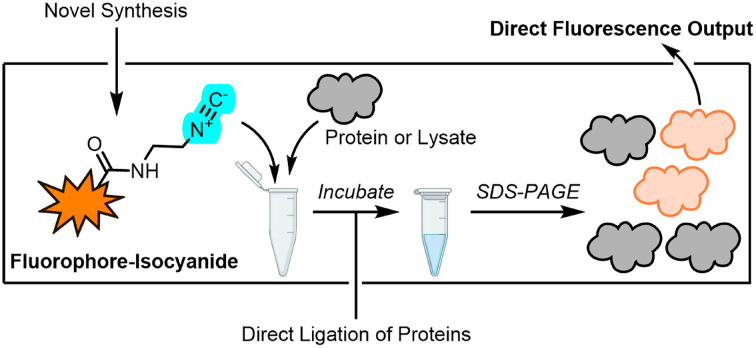

The formally divalent carbon atom of the isocyanide functional group^1^ is able to react as a nucleophile,^2^ an electrophile,^3^ a somophile,^4^ or a carbene,^5^ and is a valuable tool in organic synthesis (*e.g.* the Passerini^6^ and Ugi multicomponent reactions (Scheme 1a)).^7^ Additionally, isocyanides undergo [4+1] cycloadditions with tetrazines, furnishing 4-imino pyrazoles following loss of N_2_.^8^ In 2011, Stöckmann *et al.*^9^ highlighted the potential of this reaction as an orthogonal biomolecule ligation tool.^10^ Recently, Tu *et al.* introduced a leaving group to trigger a β-elimination and used this reaction as a bioorthogonal method of payload release.^11^ The process has since been applied to on-demand click-to-release of photosensitiser NC-DSBDP used in photodynamic therapy (Scheme 1b).^12^ Other examples of nucleophilic isocyanides in bioorthogonal ligation are also present in recent literature.^13^

**Scheme 1:**
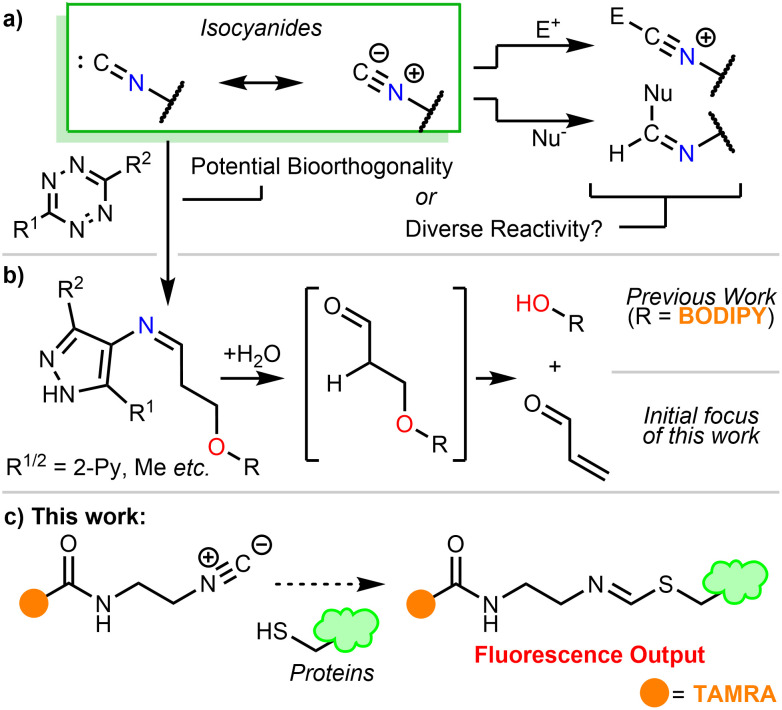
(a) The varied reactivity of isocyanides. (b) The application of isocyanides in bioorthogonal uncaging chemistry. They have previously been used as a method to release fluorophore (BODIPY) photosensitiser NC-DSBDP *in situ*. (c) This work: protein modification by fluorescent isocyanide tool compound.

A recent report by Geißler *et al.* proposed that aryl isocyanides exhibit antibiotic properties through covalent modification of cysteines,^14^ raising questions over their bioorthogonality. Isocyanide-containing natural products (*e.g.* Xanthocillin) have also shown antibiotic properties.^15^ While initially ascribed to isocyanide coordination with metal cations, covalent modification remains a plausible explanation. The ambiguous nature of the true reactivity of isocyanides in biological contexts remains underexplored.

We embarked on this study with the aim of developing bioorthogonal, click-to-release probes. We sought to utilise the isocyanide-tetrazine reaction to release a protein-reactive α,β-unsaturated carbonyl product (Scheme 1b).^11,12^ We serendipitously discovered that the isocyanide probes reacted with proteins in the absence of any additional reagents, adding weight to the recent results from Geißler *et al.*^14^ To understand the true reactivity of the isocyanide functional group in a biological context, we designed and synthesised a fluorescent probe that would allow us to unequivocally assess its suitability as a bioorthogonal reagent (Scheme 1c).

Isocyanide-containing probes 9 and 10, which were envisaged to react with tetrazines to form a reactive Michael acceptor warhead *in situ* (Scheme 1b and c), were designed and synthesised (Scheme 2). Both probes included the key 3-isocyanopropyl group and a terminal alkyne for protein visualisation *via* click chemistry, but with either a caged enal 9, or enone 10 warhead. A pivalate ester was incorporated as the leaving group for the β-elimination step, as this afforded a reasonable balance between stability and reactivity.

**Scheme 2:**
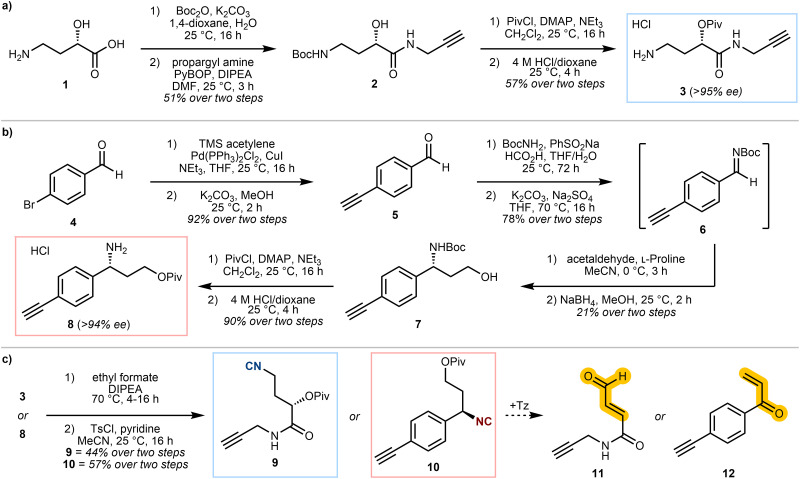
(a) Synthesis of amine 3. (b) Synthesis of amine 8. (c) Synthesis of probes 9 & 10, with hypothesised probe release with tetrazine (Tz) to form the reactive species 11 and 12. The enantiomeric excess values were estimated using Mosher's method (SI, Fig. S1).

To investigate the suitability of these probes as caged Michael acceptors, we carried out a proteome labelling experiment on U-2OS cell lysate, with varying probe concentrations. Following a click reaction with 5-carboxyrhodamine (TAMRA)-azide, the results were analysed by SDS-PAGE (Fig. 1). While a combination of probe 9 or 10 and dipyridyl tetrazine resulted in apparent labelling of the lysate, to our surprise similar results were also obtained without the use of tetrazine (Fig. 1, green box). This implied that the isocyanide on its own may covalently modify proteins, and that it may be an unsuitable precursor to uncage covalent warheads.

**Fig. 1:**
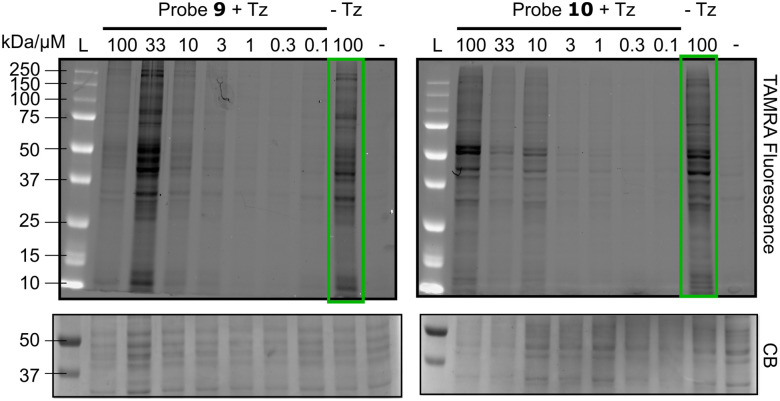
Protein profiling experiment with the isocyanide probes, analysed *via* SDS-PAGE (µM = concentrations of 9 or 10 + dipyridyl tetrazine [equimolar]; –Tz = 100 µM 9 or 10 with no tetrazine; − = DMSO; click reaction carried out on all lanes with: CuSO_4_ [100 µM], TAMRA-azide [100 µM], TCEP [tris(2-carboxyethyl)phosphine, 1.8 mM], TBTA [tris(benzyltriazolylmethyl)amine, 100 µM]; CB = Coomassie Blue stain).

Despite this, we reasoned that isocyanide uncaging may still be viable, provided that the rate of uncaging was significantly faster than the rate of isocyanide-protein modification. To investigate whether predicted product enone 12 had been liberated from 10 during the experiments in Fig. 1, we synthesised 12 and conducted a lysate labelling experiment to allow a direct comparison (Fig. S3). 12 showed considerably increased labelling compared to 10 plus tetrazine, suggesting that little, if any, enone is liberated by the reaction of probe 10 with dipyridyl tetrazine.

To clarify these results using a simplified system, we used a recombinant protein consisting of the catalytic domain of Aurora A (AurA) kinase (residues 116–389), fused with the activation domain of TPX2 (residues 7–20, TPX2:AurA, SI). TPX2:AurA was selected as this protein–protein interaction has been extensively characterised,^16^ and the construct contains three cysteine residues that could be used to assess previous literature claims that isocyanides are Cys-reactive.^14,17^ Labelling was observed by SDS-PAGE, indicating successful ligation with the protein, while no labelling was observed when centrifugal filtration was carried out to remove small molecules prior to the click reaction (Fig. S4). Analysis of the same sample *via* intact protein MS showed only the unmodified protein mass (data not shown), preventing further characterisation.

Since transition metal catalysts are known to promote *α*,*α*-insertion in organic reactions,^18^ we reasoned that the isocyanide motif may be activated by the Cu(i) catalyst during our gel-based studies. To simplify our investigation and better understand our emerging hypothesis, we reasoned that a probe directly bonded to a fluorophore would enable straightforward monitoring of isocyanide biocompatibility. TAMRA 15 was selected as the fluorophore, for its high extinction coefficient and quantum yield (Scheme 3).^19,20^ An amide coupling of mono-formylated ethylene diamine and TAMRA *N*-hydroxysuccinimide (NHS) ester, followed by dehydration *via* Burgess reagent, afforded probe 17.

**Scheme 3:**
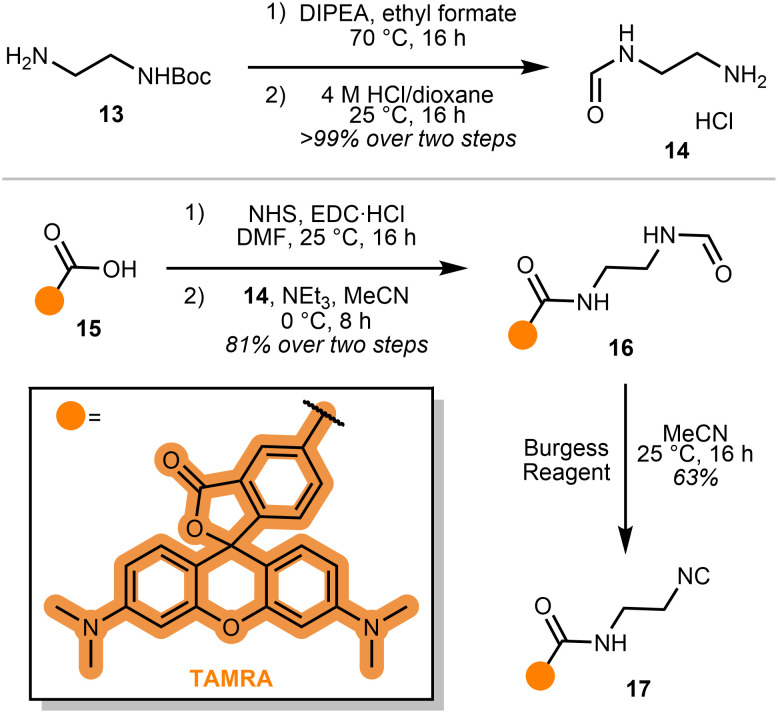
Synthesis of probe 17.

With probe 17 in hand, we carried out a gel based imaging experiment with TPX2:AurA kinase, and an additional C290A:C393A mutated AurA kinase (Fig. 2a). While both TPX2:AurA and C290A:C393A AurA contain two buried cysteine residues (C247 and C319), TPX2:AurA contains an additional solvent-exposed cysteine (C290) that is absent in the C290A:C393A AurA (see SI for sequences).

**Fig. 2:**
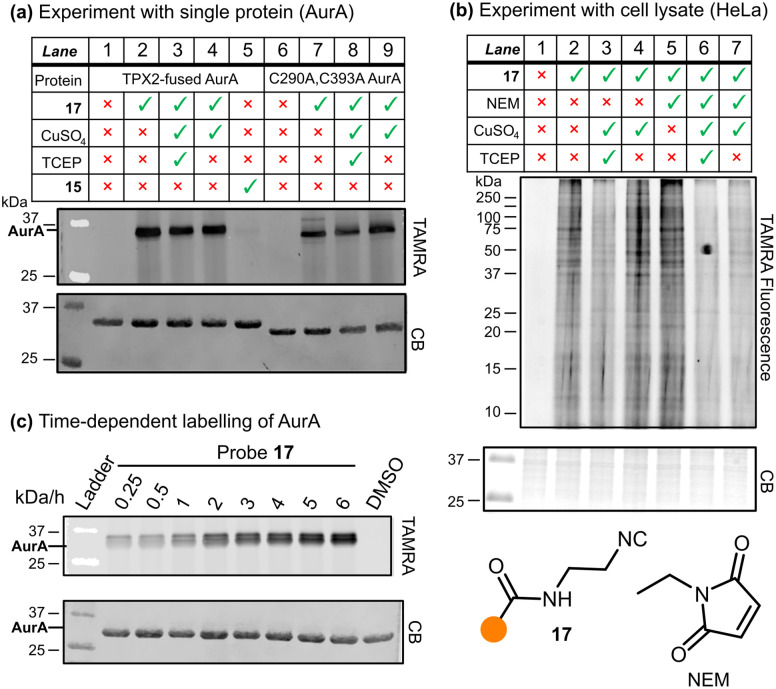
(a) Labelling experiment with the two mutants of AurA kinase, analysed *via* SDS-PAGE. 15 = TAMRA-CO_2_H. CB = Coomassie. (b) Competitive labelling experiment on HeLa cell lysate, analysed *via* SDS-PAGE. (c) Time-dependent labelling of AurA.

Incubation of probe 17 with TPX2:AurA (Fig. 2a, Lane 2) showed strong labelling, confirming the hypothesis that isocyanides covalently modify proteins in the absence of any additional reagents (control Lanes 1, 5 and 6). The addition of click reagents (Lanes 3 and 4) led to no observable difference in the reactivity profile. Similarly, probe 17 successfully modified C290A:C393A AurA kinase, with or without the copper reagents (Lanes 7–9). Given that buried residues C247 and C319 are generally considered to be challenging to modify,^21^ this result indicates that probe 17 may label other functional groups than cysteine.

To understand isocyanide reactivity in a more complex system, this experiment was repeated using HeLa cell lysate (Fig. 2b); as before, probe 17 was incubated with or without the click reagents (Lanes 2, 3, and 4). Similar to the AurA experiments, 17 extensively labelled the proteome. The addition of the copper catalyst, regardless of its oxidation state (controlled by the addition of the reducing agent TCEP), did not appear to affect the labelling significantly. To investigate the residue selectivity of isocyanide labelling, we introduced competition experiments against *N*-ethyl maleimide (NEM), a known covalent modifier for cysteine residues (Lane 5).^22,23^ This resulted in a retention of signal, suggesting the site of modification by probe 17 is not exclusive to cysteine residues. This aligns with the broad reactivity profile demonstrated by isocyanides in small molecule chemistry (Scheme 1a). Intriguingly, Geißler *et al.* carried out a similar experiment with *S. aureus* NCTC 8325 lysate and iodoacetamido-alkyne which indicated that their isocyanide probes covalently modified cysteine residues;^14^ however, our findings suggest that other residues must also be considered as potential ligation handles. When the competition experiment with NEM was repeated in the presence of a copper catalyst (Lanes 6 and 7), the intensity of the signals diminished. However, the Cu-catalysed click reaction can cause oxidative protein damage and aggregation,^24^ complicating interpretation of this result.

We next sought to better understand the specificity and extent of this isocyanide-protein modification. Benzyl isocyanide was incubated with an assortment of nucleophilic amino acids and its stability was monitored by ^1^H NMR spectroscopy (Fig. S5). Under these conditions, reaction of the isocyanide was observed only with cysteine, generating a species consistent with the imidothiolate adduct proposed by Geißler *et al.*^14^ This process was relatively slow, modifying ∼13% of the benzyl isocyanide over 24 hours. Hydrolysis of benzyl isocyanide to the corresponding formamide was also observed in the presence of aspartate (Fig. S5), indicating isocyanide lability in the presence of carboxylic acids.

Further tests using Ellman's assay with glutathione and benzyl isocyanide also afforded 13% modification over 16 hours (Table S1). As small molecule reactivity is not always representative of reaction rate in larger biomolecules, we next tested TPX2:AurA with either probe 17 or TAMRA-maleimide and compared the relative fluorescence outputs by SDS-PAGE (Fig. S6). After 2 hours of incubation, probe 17 produced a signal that was equivalent to just 0.2% of TAMRA–maleimide. This supported our earlier findings that the reaction of isocyanides with cysteines is a relatively inefficient process; however, the imidothiolate adduct may hydrolyse to the formamide under these conditions, meaning the resulting readout may not be fully representative.

Given this relatively low level of modification, we investigated the rate of reaction between isocyanides and proteins. Slower reaction rates may permit isocyanides to be applied in bioorthogonal settings in conjunction with a faster-acting reagent. Probes 10 and 17 were incubated with TPX2:AurA or HeLa lysate, quenched with acetone at various timepoints, and visualised *via* SDS-PAGE (Fig. 2c, S7). Clear labelling was observed after 15 minutes and increased up to 3 hours, timescales similar to those applied in bioorthogonal experiments.^10,11^ Interestingly, a further increase in fluorescence intensity was observed between 5 and 6 hours, suggesting further reaction pathways at longer timepoints.

Isocyanides are a prevalent motif within organic chemistry, yet their behaviour in a biological setting is poorly understood. While they have been identified in natural products and antibacterials, they have also found application in bioorthogonal click-to-release strategies. Our results using a fluorophore-linked isocyanide provide evidence that isocyanides cannot be considered bioorthogonal in chemical biology. Probe 17 was found to modify proteins in the absence of additional reagents on intact protein and in cell lysate. Our small molecule studies provided direct evidence for the reaction between isocyanides and cysteines; however our protein and cell lysate experiments also showed that further reactivity with additional residues cannot be ruled out. The labelling efficiency of the protein-isocyanide reaction appears to be negligible in comparison to established warheads such as maleimide by SDS-PAGE, although the likely instability of the resulting adduct makes accurate quantification challenging. Taken together, these findings indicate that if an isocyanide click-to-release strategy is used in a biologically relevant media, careful experimental design is needed to avoid unwanted side reactions.

Nevertheless, these results provide an opportunity to apply isocyanides as a novel warhead in covalent protein modification. Straightforward incorporation of the motif afforded easily detectable adducts in both single protein and cell lysate studies. Further mechanistic studies are required to fully characterise the residue selectivity of isocyanide-protein ligation, and these are currently underway in our laboratory.

## Author contributions

RN and VS synthesised the probes; RN performed protein modification experiments; JAM cloned and expressed AurA recombinant proteins; KL, SLW, PR and MHW provided supervisor support and intellectual input; SLW and MHW conceptualised the work; MDM secured funding for aspects of the research; RN, KL, and SLW wrote the manuscript with additional input from all authors.

## Conflicts of interest

There are no conflicts of interest to declare.

## Supplementary Material

CC-062-D5CC04136H-s001

## Data Availability

The data supporting this article have been included as part of the supplementary information (SI). Supplementary information: supplementary figures, and synthetic protocols and characterisation for all compounds. See DOI: https://doi.org/10.1039/d5cc04136h.
