# Supplementary material for: Investigating the bioorthogonality of isocyanides
Source: Chem Commun (Camb). 2025 Dec 15;62(6):1945–9. doi: 10.1039/d5cc04136h (PMC12730495; doi:10.1039/d5cc04136h)
Supplement: CC-062-D5CC04136H-s001 [file CC-062-D5CC04136H-s001.pdf]

Supplementary Information (SI) for ChemComm.
This journal is © The Royal Society of Chemistry 2025

Supplementary Information

Investigating the Bioorthogonality of Isocyanides

Ryota Nakao,[a] Viktor Savic,[b] Jennifer A. Miles,[c] Keith Livingstone,[a] Marko D. Mihovilovic,[b] Piotr Raubo,[d] Stuart L. Warriner,[a]* Megan H. Wright [a]*

a. School of Chemistry and Astbury Centre for Structural Molecular Biology, University of Leeds, Leeds, LS2 9JT, United Kingdom

b. Institute of Applied Synthetic Chemistry, TU Wien, Getreidemarkt 9/163-OC, 1060 Vienna, Austria

c. Faculty of Biological Sciences and Astbury Centre for Structural Molecular Biology, University of Leeds, Leeds, LS2 9JT, United Kingdom

d. Research and Early Development, Oncology R&D, AstraZeneca, Cambridge, CB2 0AA, United Kingdom

Contents

Supplementary Figures ... 3
Methods ... 8
TPX2 fused Aurora A Kinase Sequence[1] ... 8
C290A, C393A mutated Aurora A Kinase Sequence[2] ... 8
Cell Culture ... 9
Lysate preparation ... 9
Gel-based Labelling Experiments ... 9
SDS-PAGE ... 10
Ellman's Assay ... 10
Chemical Synthesis ... 10
General Considerations ... 10
Synthesis of 9 ... 11
Synthesis of 10 ... 15
Synthesis of 17 ... 20
Synthesis of 12 ... 23
Synthesis of Mosher's Amides S1 and S2 ... 24
References ... 24
NMR Spectra ... 26

Supplementary Figures

(a) Diagnostic Peaks in ^{19}F and ^{1}H NMR Spectrum in CD_3OD for S1 (Estimated *ee* >95%)

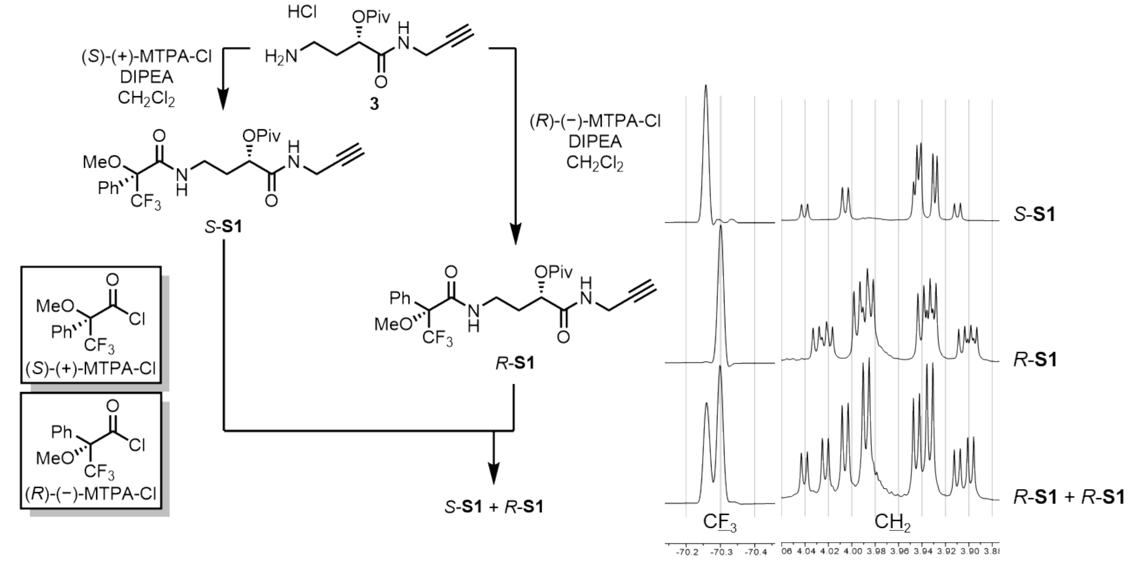

(b) Diagnostic Peaks in ^{19}F and ^{1}H NMR Spectra in $CDCl_3$ for S2 (Estimated *ee* >94%)

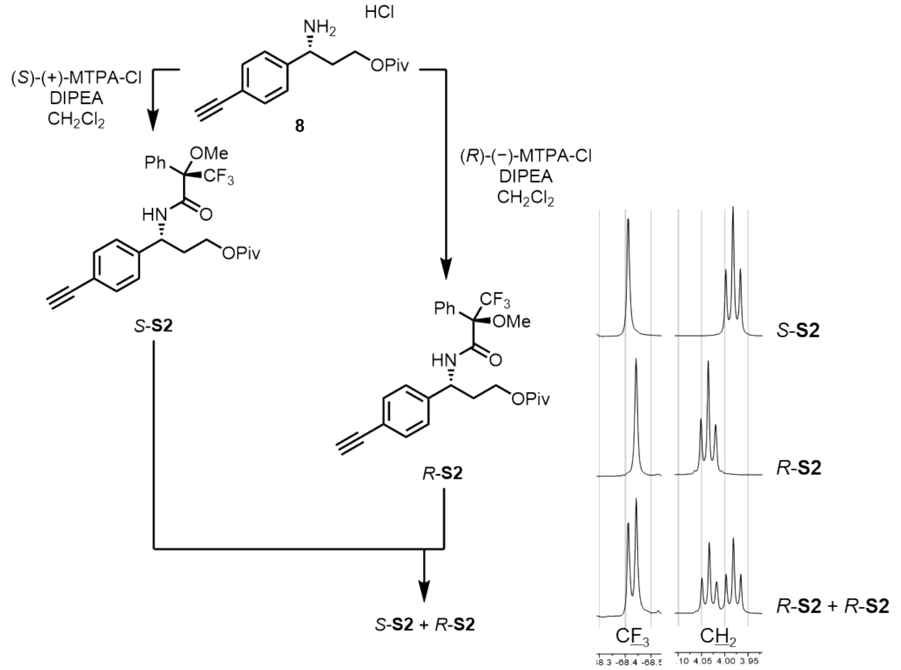

Figure S1. *The ee of probes* ***9*** *and* ***12*** *determined via derivatisation to the Moshers amides.* ***(a)*** *Salient regions in ^{19}F and ^{1}H spectrum of R/S-**S1**.* ***(b)*** *Salient regions in ^{19}F and ^{1}H spectrum of R/S-**S2**.*

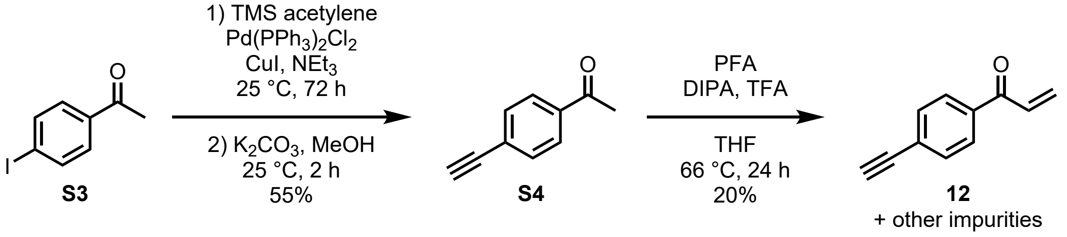

Figure S2. *Synthesis of enone probe* ***12,*** *the envisaged product of the isocyanide-tetrazine reaction of probe* ***10*** *(unoptimised).*

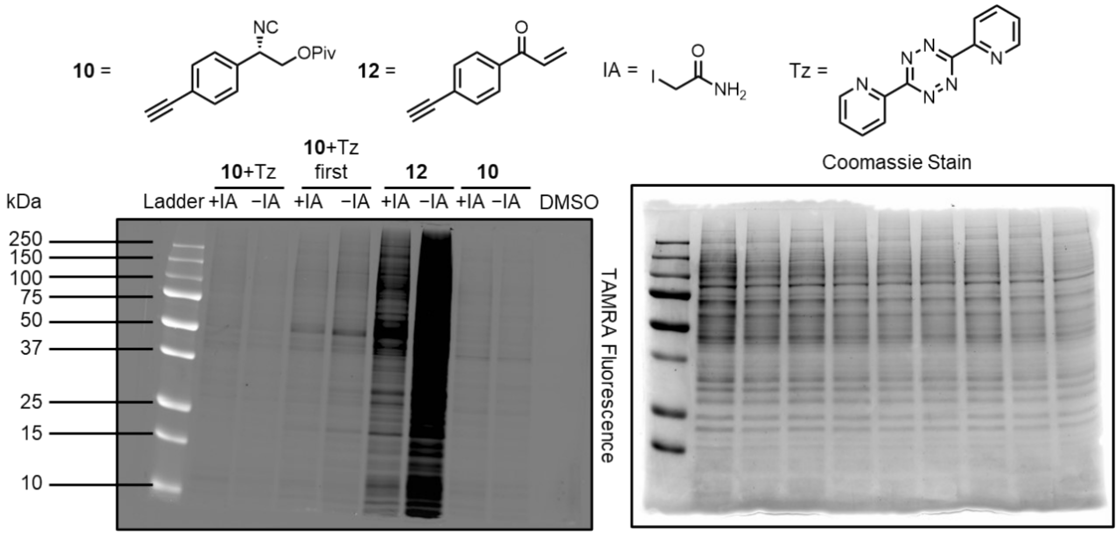

Figure S3. *A competitive proteomics experiment with iodoacetamide (IA) and the uncaged enone probe* ***12****, independently synthesised to compare with the isocyanide probe* ***10*** *with dipyridyltetrazine (Tz), analysed via SDS-PAGE. "****10****+Tz first" represents the lanes where isocyanide and dipyridyltetrazine were mixed first, for 30 minutes prior to the addition of the lysate. Probe* ***10****, tetrazine, probe* ***12*** *and iodoacetamide all at 100 μM concentration. In-gel fluorescence scanning and Coomassie staining are shown.*

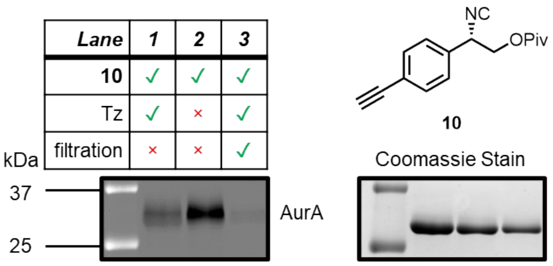

Figure S4. *An experiment with AurA kinase comparing probe* ***10*** *with dipyridyltetrazine (Lane 1), without dipyridyltetrazine (Lane 2), and without dipyridyltetrazine when the sample was filtered via centrifugal filtration (10 kDa filter) before carrying out the click reaction (Lane 3). In-gel fluorescence scanning and Coomassie staining are shown.*

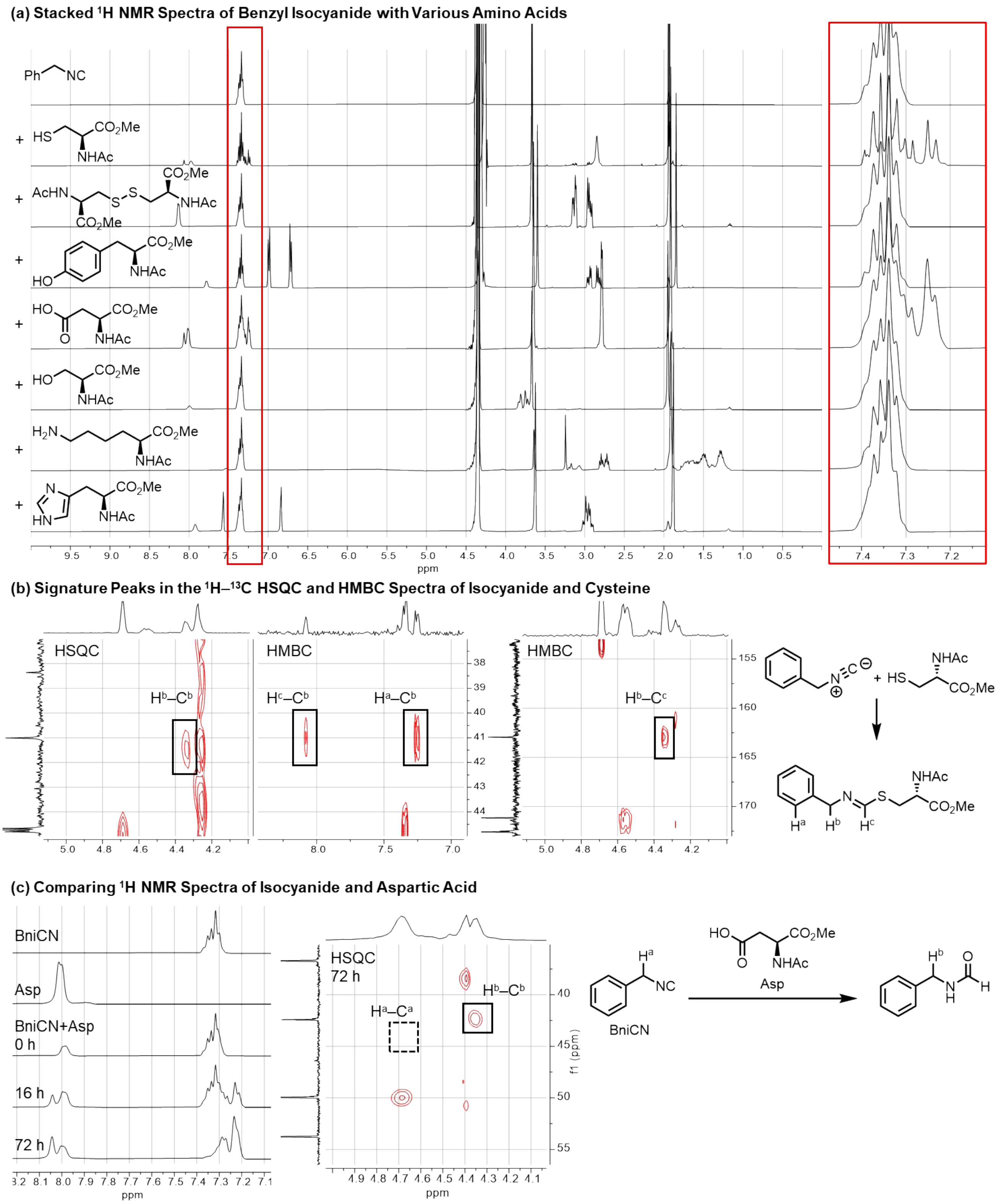

Figure S5. ***(a)*** *^{1}H NMR spectra of benzyl isocyanide (BniCN, 80 mM) with or without various protected amino acids (80 mM), taken after 24 hours of incubation at 25 °C in D_2O (30 μL), PBS buffer (in H_2O, 270 μL) and CD_3CN (200 μL) (spectra acquired with solvent suppression). Aromatic region zoomed in on the right, where additional peaks were observed when benzyl isocyanide was incubated with N-acetyl cysteine methyl ester or N-acetyl aspartate methyl ester, but not with others;* ***(b)*** *Relevant*

correlations in the HSQC and HMBC spectra of BniCN + Cysteine, where the adduct was observed; ***(c)*** *Relevant region of BniCN + Aspartate spectra; under these conditions, by 72 h the isocyanide had hydrolysed to the formamide, as shown by HSQC correlation (loss of Ha-Ca and appearance of Hb-Cb crosspeaks) and appearance of the formamide proton at 8.06 ppm.*

Table S1. *Ellman's assay; Absorbance values recorded at 412 nm.*

**Entry**	**Probe**	**1**	**2**	**3**	**Mean**
1	DMSO Control	0.225	0.246	0.236	0.236
2	Iodoacetamide	−0.017	−0.041	−0.029	−0.029
3	Bn-isocyanide	0.197	0.202	0.216	0.205

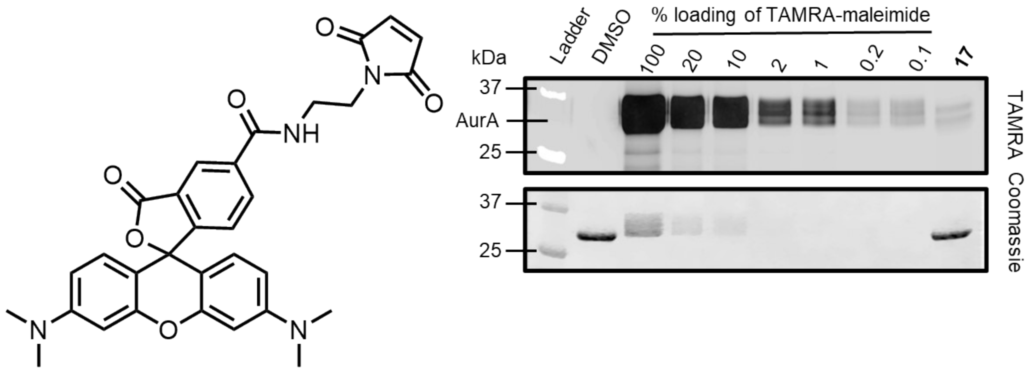

TAMRA-maleimide

Figure S6. *An experiment with AurA kinase comparing the extent of protein modification by TAMRA-maleimide and probe* ***17****. The loading percentage was varied to understand the labelling efficiency of the isocyanide* ***17****, relative to TAMRA-maleimide. This appeared to be comparable when the loading was of TAMRA-maleimide was 0.2%, when the band intensities were measured by ImageJ.*

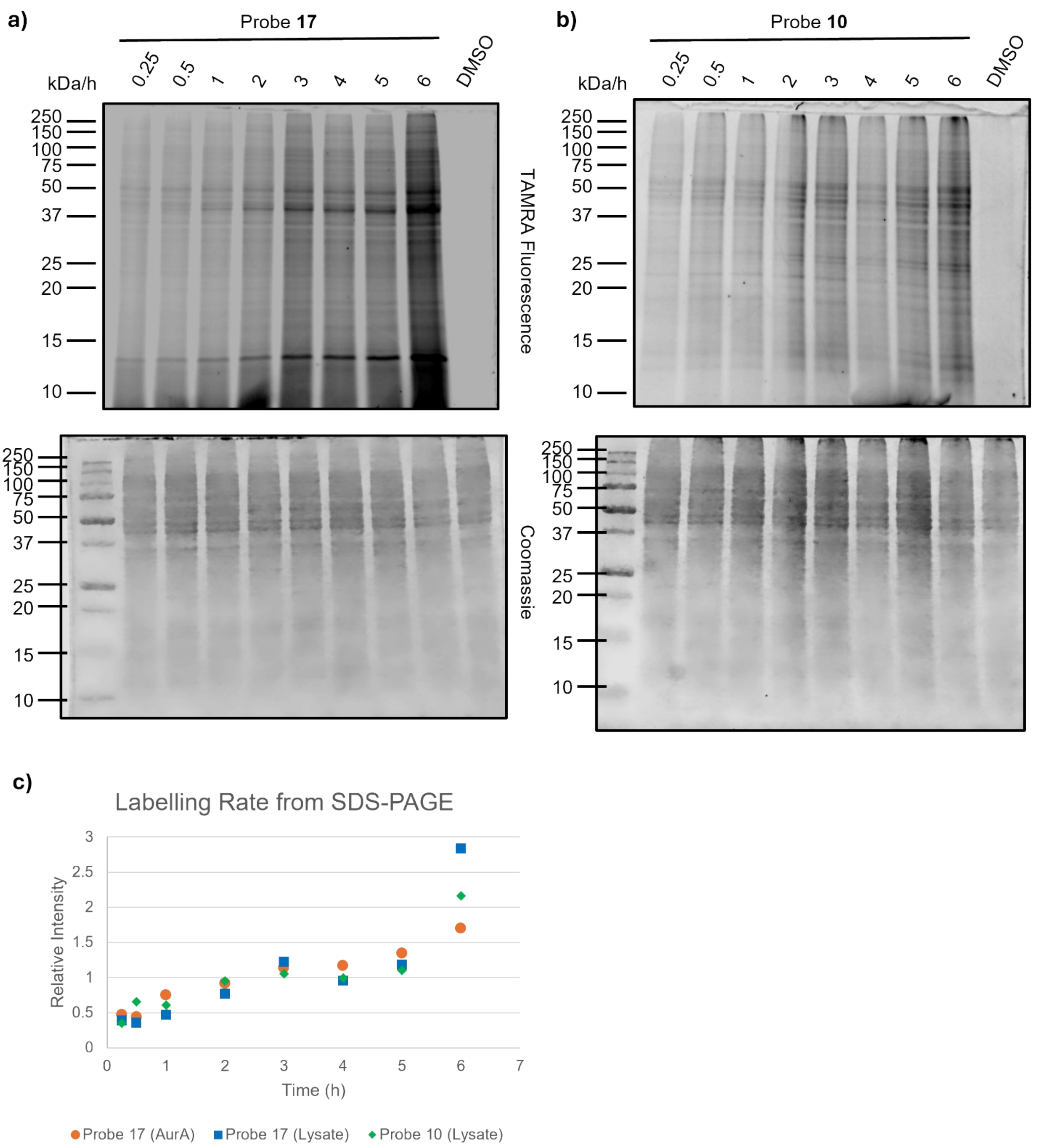

Figure S7. *Evaluation of time-dependent labelling of probe **17** **(a)** and probe **10** **(b)** with lysate. Probe was incubated with lysate for the indicated times at 100 µM; samples were quenched through the addition of ice-cold acetone, proteins precipitated and resuspended. Probe **10** samples were subject to click conditions, and then all samples analysed by SDS-PAGE. Band intensities were measured by ImageJ and normalised to Coomassie stain for loading before plotting **(c)** Main text figure 2c data (probe **17** + AurA) were also plotted.*

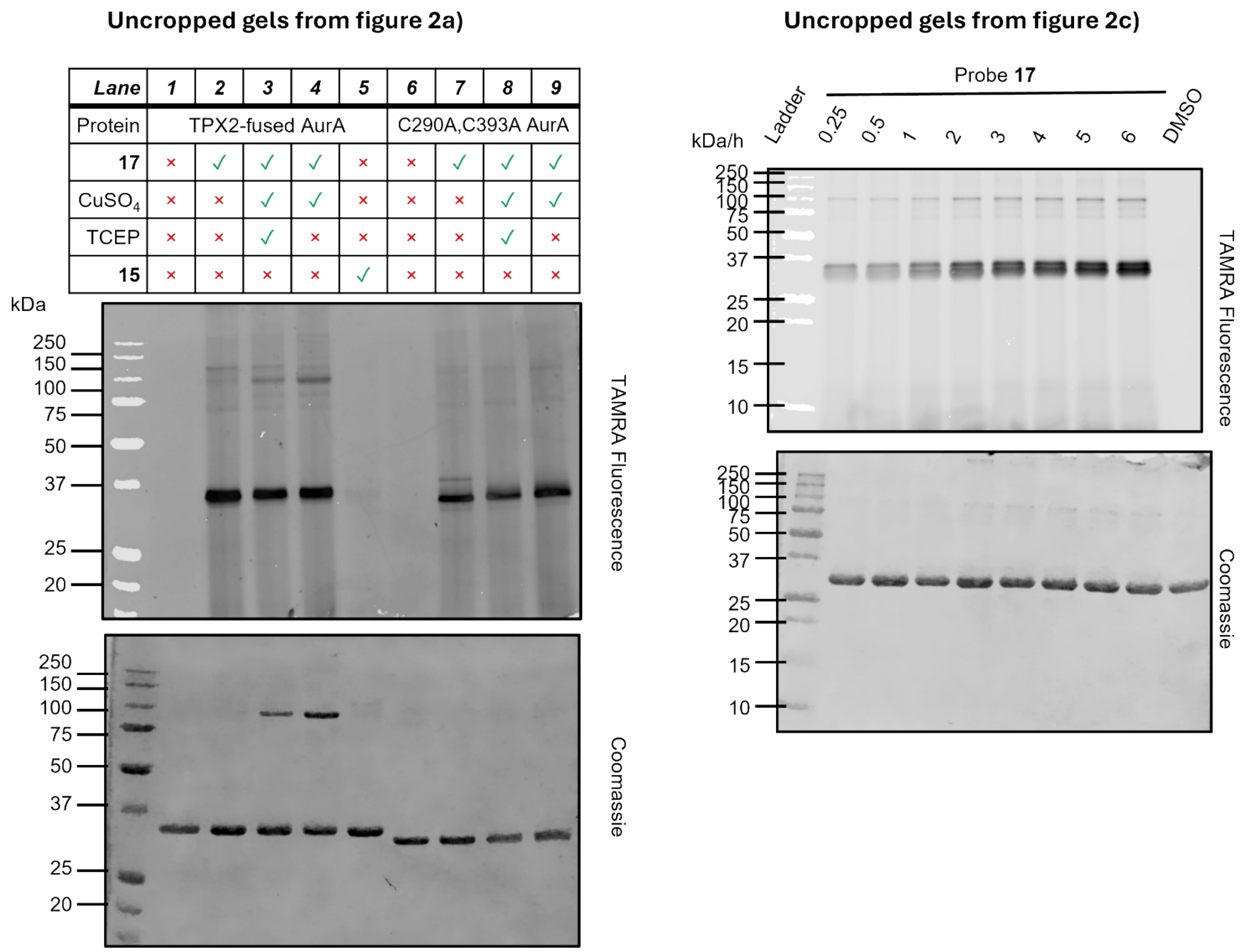

Figure S8. *Uncropped gels from AurA experiments shown in main text figure 2.*

Methods

TPX2 fused Aurora A Kinase Sequence[1]

GAMSYSYDAPSDFINFSSKQKNEESKKRQWALEDFEIGRPLGKGKFGNVYLAREKQSKFILALKVLFKAQLEKAGVEHQLRREVEIQSHLRHPNILRLYGYFHDATRVYLILEYAPLGTVYRELQKLSKFDEQRTATYITELANALSYCHSKRVIHRDIKPENLLLGSAGELKIADFGWSVHAPSSRRTTLCGTLDYLPPEMIEGRMHDEKVDLWSLGVLCYEFLVGKPPFEANTYQETYKRISRVEFTFPDFVTEGARDLISRLLKHNPSQRPMLREVLEHPWITANSSK

C290A, C393A mutated Aurora A Kinase Sequence[2]

GAMESKKRQWALEDFEIGRPLGKGKFGNVYLAREKQSKFILALKVLFKAQLEKAGVEHQLRREVEIQSHLRHPNILRLYGYFHDATRVYLILEYAPLGTVYRELQKLSKFDEQRTATYITELANALSYCHSKRVIHRDIKPENLLLGSAGELKIADFGWSVHAPSSRRTTLAGTLDYLPPEMIEGRMHDEKVDLWSLGVLCYEFLVGKPPFEANTYQETYKRISRVEFTFPDFVTEGARDLISRLLKHNPSQRPMLREVLEHPWITANSSKPSNAQNKESASKQS

Cell Culture

HeLa and U-2 OS cells were cultured in Dulbecco's Modified Eagle Medium (DMEM, high glucose, 4.5 g/L) supplemented with 10% fetal bovine serum (FBS) and 1% Penicillin Streptomycin (Pen Strep) at 37 °C in a 5% CO_2 atmosphere. Trypsin was used for cell detachment, pelleted at 500 × G for 5 min and washed with PBS buffer to remove excess trypsin. Once a confluency of around 80% was reached, cells were collected, pelleted and lysed for labelling experiments.

Lysate preparation

HeLa cells at confluent density (4 × 106 cells/mL) in a T75 were trypsinised, collected in a centrifuge tube. Cell pellets were centrifuged (3000 × g, 5 min, 4 °C) and the supernatant was discarded. HeLa cell pellets were washed with PBS buffer, taken up in PBS buffer (1 mL) followed by cOmplete Mini, EDTA free protease inhibitor cocktail (100 µL from a 10× stock solution, Merck part number: 11836170001). Cells were physically lysed by sonication (3 × 10 s pulses at 40% intensity) on ice, with cooling for 30 seconds between sonication. Unlysed cells were pelleted by centrifugation for 15 minutes (1000 × G, 4 °C) and the supernatant transferred to a new Eppendorf™ tube. The protein concentration was determined by Pierce BCA assay, and aliquots of lysate were snap-frozen in liquid nitrogen and stored at −80 °C.

Gel-based Labelling Experiments

HeLa cell lysate, U-2 OS cell lysate or AurA kinase protein, and probe (2.5 µL, 10%v/v DMSO/PBS) were dissolved in PBS buffer to give the volume of 25 µL at 1 mg/mL lysate proteins or 20 µM isolated protein. The samples were incubated for 30 minutes at 25 °C and 700 rpm. If tetrazine or competitor addition was required, tetrazine or competitor (2.0 µL, 10%v/v DMSO/PBS) was added and were incubated for a further 30 minutes at 25 °C and 700 rpm. 10% SDS buffer (1.25 µL) was added, followed by 2 µL of the click reagent mix, consisting of TAMRA-azide (6 µL, 10 mM in DMSO), $CuSO_4$ (12 µL, 50 mM in H_2O), TCEP (24 µL, 50 mM in H_2O), and TBTA (6 µL, 10 mM in DMSO).[1] The samples were incubated for 1 hour at 25 °C and 550 rpm and quenched with 0.5 µL EDTA solution (0.5 M in H_2O) and cold acetone (100 µL) was added. The samples were precipitated for 16 hours at −5 °C. The samples were centrifuged at 4 °C for 10 minutes at 13,000 × G, the supernatant removed, and cold MeOH (100 µL) was added. This process was repeated twice, before the supernatant was removed and the samples were left to air dry for 30 minutes.

[1] In the case of probe **17**, the TAMRA-azide was left out since the fluorophore is already attached. In the case of applying Cu(II) alone, TCEP and TBTA were replaced with equal volumes of their solvents (H_2O or DMSO respectively).

SDS-PAGE

Protein/lysate samples (12.5 µL in 2% w/v SDS in PBS buffer) were combined with dithiothreitol (DTT) (2 µL, 500 mM) and protein loading buffer (10 µL, final concentration 50 mM Tris HCl, 2% w/v SDS, 10% v/v glycerol, 0.1% w/v bromophenol blue dye, pH 6.8). Proteins were denatured by boiling for 2 minutes at 95 °C. Samples were loaded into hand-cast 1.5 mm 10- or 15-well SDS-PAGE gels (12% or 15% acrylamide) in running buffer (25 mM Tris, 192 mM glycine, 0.1% w/v SDS) in a miniPROTEAN® Tetra Cell tank. All Blue Precision Plus Protein™ standards (10 µL, 10 – 250 kDa) were loaded and the gel run at 180 V for 50 minutes and imaged on a BioRad GelDoc XR Molecular Imager or a LI-COR Odyssey XF Imager. Gels were stained for 16 hours in Coomassie blue stain (2.5 g Coomassie blue, 100 mL AcOH, 400 mL MeOH, 500 mL H_2O) and de-stained for at least 6 hours in de-stain solution (40% methanol, 10% acetic acid) prior to re-imaging. If required, bands were quantified by ImageJ.

Ellman's Assay

Glutathione (20 µM) was incubated with iodoacetamide (100 µM) or benzyl isocyanide (100 µM) for an hour at 25 °C, followed by the addition of Ellman's reagent (1.0 mM) and incubation for 16 hours at 25 °C. UV-Vis spectra were recorded using Thermo Scientific™ NanoDrop™ One Microvolume UV-Vis Spectrophotometer, which was baseline corrected to a mixture of isocyanide (100 µM) and Ellman's reagent (1.0 mM), incubated for 16 hours at 25 °C.

Chemical Synthesis

General Considerations

All reactions were carried out under nitrogen atmosphere and anhydrous conditions unless stated otherwise. All reagents were used as supplied by SigmaAldrich/Merck Ltd (Gillingham, Dorset, UK), Fluorochem (Hadfield, Derbyshire, UK) or Tokyo Chemical Industry (Tokyo, Japan) without further purification apart from triethylamine, *N,N*-diisopropylethylamine, pyridine, propargylamine, and pivaloyl chloride, which were distilled over CaH_2 immediately before use. Innovative Technology PureSolv MD 6 solvent purification system was used to dry and deoxygenate solvents: CH_2Cl_2, THF, MeCN, MeOH prior to use, and *N,N*-dimethylformamide (DMF) was used as supplied by Thermo Fisher (Massachusetts, US) as extra dry over molecular sieves, AcroSeal™. All aqueous solutions were prepared in deionised water. Merck aluminium-backed plates precoated with silica (0.2 mm, 60 F_{254}) were used for thin layer chromatography (TLC) and visualised either with a potassium permanganate stain or a phosphomolybdic acid stain, prepared using standard recipes.[3] Flash column chromatography was performed on silica gel (Merk Kieselgel 60 F_{254} 230 – 400 mesh) using an appropriate eluent system, reported as % volume of the more polar eluent in the less polar eluent.[4] Infrared (IR) spectra were recorded on a Bruker ALPHA II FT-IR as solids or neat liquids, and

only selected absorbances (v_{max}) are reported. Proton nuclear magnetic resonance (^{1}H NMR) spectra were recorded at 501 MHz, and proton decoupled carbon nuclear magnetic resonance ($^{13}C\{^{1}H\}$ NMR) spectra were recorded at 126 MHz, both on Bruker AV4 NEO 11.75 T spectrometer at 25 °C. Chemical shifts are reported on the δ scale in parts per million (ppm) using residual solvent signals.[5] Coupling constants (*J*) are quoted to 1 decimal place in Hz along with the splitting abbreviations as follows: s = singlet, d = doublet, t = triplet, q = quartet, hept = heptet, m = multiplet, br = broad, app. = apparent. These values were calculated using MestReNova NMR software. ^{1}H–^{1}H COSY, ^{1}H–^{13}C HSQC, and ^{1}H–^{13}C HMBC spectra were utilised to assign peaks. For complicated compounds, signal assignments are described using IUPAC convention. Electrospray ionization (ESI) was used to ionize molecules and ions were measured by time of flight (TOF) for high resolution mass spectrometry (HRMS), obtained with Bruker MaXis Impact.

Synthesis of 9

(2*S*)-4-{[(*tert*-Butoxy)carbonyl]amino}-2-hydroxybutanoic acid[6] **S5**

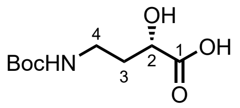

To a solution of (*S*)-4-amino-2-hydroxybutanoic acid (500 mg, 4.20 mmol, 1 equiv) and K_2CO_3 (1.45 g, 10.5 mmol, 2.5 equiv) in H_2O (10 mL), a solution of di-*tert*-butyl dicarbonate (1.05 g, 4.83 mmol, 1.2 equiv) in 1,4-dioxane (5.5 mL) was added dropwise. The reaction mixture was stirred at 25 °C and monitored by the ninhydrin test. After 16 hours, the reaction was complete and the reaction mixture was extracted with Et_2O (2 × 20 mL), then the aqueous layer was acidified to pH = 1 using HCl (2 M in H_2O) and saturated with NaCl. The aqueous layer was extracted with EtOAc (5 × 50 mL), and the organic layer was dried over Na_2SO_4, filtered, and the solvent was evaporated under reduced pressure to yield the title compound as a colourless oil (921 mg, 4.20 mmol, >99% yield).

R_f = 0.40 (10% EtOAc/hexane)
$[\alpha]_D^{20}$ −4.28 (*c* 0.5, MeOH)
^{1}H NMR (501 MHz, DMSO-*d6*) δ 6.76 (t, *J* = 6.0 Hz, 1H, N<u>H</u>), 3.93 (dd, *J* = 8.6, 3.9 Hz, 1H, 2-C<u>H</u>), 3.01 (app. q, *J* = 6.0 Hz, 2H, 4-C$\underline{H_2}$), 1.84 – 1.70 (m, 1H, 3-C$\underline{H}_{2A}$), 1.64 – 1.51 (m, 1H, 3-C$\underline{H}_{2B}$), 1.37 (s, 9H, C(C$\underline{H}_3$)$_3$).
Analytical data consistent with that reported previously.[6]

tert-Butyl (3-hydroxy-4-oxo-4-(prop-2-yn-1-ylamino)butyl)carbamate **2**

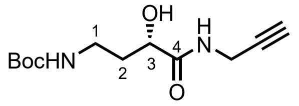

To a solution of hydroxy acid **S5** (100 mg, 0.46 mmol, 1 equiv) in DMF (2mL), a solution of benzotriazol-1-yloxytripyrrolidinophosphonium hexafluorophosphate (PyBOP, 290 mg, 0.55 mmol, 1.2 equiv) in DMF (2 mL) was added and stirred at 20 °C for 10 minutes. A solution of propargylamine (40 µL, 0.55 mmol, 1.2 equiv) and *N*,*N*-diisopropylethylamine (120 μL, 0.68 mmol, 1.5 equiv) in DMF (2 mL) was added to the reaction mixture dropwise. The reaction mixture was stirred at 25 °C for 3 hours. The reaction mixture was diluted with EtOAc (50 mL), washed with saturated $NaHCO_3$ (3 × 100 mL), H_2O (3 × 100 mL) and brine (3 × 100 mL). The organic layer was dried over Na_2SO_4, filtered, and the solvent was evaporated under reduced pressure. The crude material was purified by flash column chromatography (EtOAc) to yield the title compound as a white solid (61 mg, 0.24 mmol, 52% yield).

R_f = 0.43 (EtOAc)

$[\alpha]_D^{20}$ −26.64 (*c* 0.5, MeOH)

FTIR (neat) cm^{-1}: 3297 (m, br, OH, NH stretch), 2979 (m, CH stretch), 1697 (s, CO stretch), 1638 (s, CO stretch).

^{1}H NMR (501 MHz, $CDCl_3$) δ 7.21 (s, 1H, amide N$\underline{H}$), 5.02 (t, *J* = 6.4 Hz, 1H, carbamate N$\underline{H}$), 4.92 (s, br, 1H, O$\underline{H}$), 4.13 – 4.07 (m, 1H, 3-C$\underline{H}$), 4.06 (ddd, *J* = 17.5, 5.7, 2.7 Hz, 1H, $\underline{H}_{2A}$CC≡C), 4.05 (ddd, *J* = 17.5, 5.7, 2.7 Hz, 1H, $\underline{H}_{2B}$CC≡C), 3.53 – 3.42 (m, 1H, 1-C$\underline{H}_{2A}$), 3.18 – 3.08 (m, 1H, 1-C$\underline{H}_{2B}$), 2.22 (app. t, *J* = 2.7 Hz, 1H, C≡C$\underline{H}$), 2.03 (dddd, *J* = 14.3, 10.9, 5.0, 3.5 Hz, 1H, 2-C$\underline{H}_{2A}$), 1.65 (app. ddt, *J* = 14.3, 10.2, 4.0 Hz, 1H, 2-C$\underline{H}_{2B}$), 1.42 (s, 9H, C(C$\underline{H}_3$)$_3$).

^{13}C NMR (126 MHz, $CDCl_3$) δ 173.6 (4-$\underline{C}$=O), 158.1 (carbamate $\underline{C}$=O), 80.4 ($\underline{C}$≡CH), 79.5 (C≡$\underline{C}$H), 71.7 ($\underline{C}$(CH$_3$)$_3$), 68.8 (3-$\underline{C}$H), 36.4 (1-$\underline{C}$H$_2$), 35.4 (2-$\underline{C}$H$_2$), 28.9 (H$_2$$\underline{C}$C≡C), 28.4 (C($\underline{C}H_3$)$_3$).

HRMS (ESI-TOF) m/z: $[M + Na]^+$ Calcd for $C_{12}H_{20}N_2O_4Na$ 279.1315; Found 279.1318 Δ = 1.07 ppm.

4-((*tert*-Butoxycarbonyl)amino)-1-oxo-1-(prop-2-yn-1-ylamino)butan-2-yl pivalate **S6**

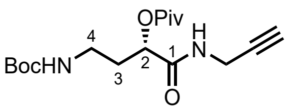

To a solution of hydroxy amide **2** (300 mg, 1.17 mmol, 1 equiv) in CH_2Cl_2 (2 mL), a solution of triethylamine (400 µL, 2.93 mmol, 2.5 equiv) and 4-dimethylaminopyridine (15 mg, 0.12 mmol, 0.1 equiv) in CH_2Cl_2 (3 mL) was added. A solution of pivaloyl chloride (250 μL, 2.00 mmol, 1.7 equiv) in CH_2Cl_2 (5 mL) was added to the reaction mixture dropwise. The reaction mixture was stirred at 25 °C for 48 hours. The reaction mixture was diluted with CH_2Cl_2 (20 mL), washed with saturated $NaHCO_3$ (3 × 20 mL), saturated NH_4Cl (3 × 20 mL) and brine (3 × 20 mL). The organic layer was dried over Na_2SO_4, filtered, and the solvent was evaporated under reduced pressure. The crude material was purified by flash column chromatography (0 – 50% EtOAc/hexane) to yield the title compound as a colourless oil (230 mg, 0.67 mmol, 57% yield).

R_f = 0.14 (30% EtOAc/hexane)

$[\alpha]_D^{20}$ −44.96 (*c* 0.5, MeOH)

FTIR (neat) cm^{-1}: 3258 (m, br, OH, NH stretch), 2974 (m, CH stretch), 1707 (s, CO stretch), 1686 (s, CO stretch), 1663 (s, CO stretch).

^{1}H NMR (501 MHz, $CDCl_3$) δ 6.55 (s, br, 1H, amide N$\underline{H}$), 5.14 (dd, *J* = 7.4, 6.5 Hz, 1H, 2-C$\underline{H}$), 4.92 (s, br, 1H, carbamate N$\underline{H}$), 4.06 (ddd *J* = 17.6, 5.3, 2.6 Hz, 1H, $\underline{H}_{2A}$CC≡C), 3.98 (ddd, *J* = 17.6, 5.3, 2.6 Hz, 1H, $\underline{H}_{2B}$CC≡C), 3.27 (app. dq, *J* = 12.6, 6.6, 1H, 4-C$\underline{H}_{2A}$), 3.10 (app. dq, *J* = 12.6, 6.4 Hz, 1H, 4-C$\underline{H}_{2B}$), 2.23 (app. t, *J* = 2.6 Hz, 1H, C≡C$\underline{H}$), 2.05 (app. ddt, *J* = 13.5, 7.4, 6.5 Hz, 1H, 3-C$\underline{H}_{2A}$), 1.95 (app. dq, *J* = 13.5, 6.5 Hz, 1H, 3-C$\underline{H}_{2B}$), 1.39 (s, 9H, Boc C(C$\underline{H}_3$)$_3$), 1.24 (s, 9H, Piv C(C$\underline{H}_3$)$_3$).

^{13}C NMR (126 MHz, $CDCl_3$) δ 177.3 (ester $\underline{C}$=O), 169.6 (1-$\underline{C}$=O), 156.1 (carbamate $\underline{C}$=O), 79.5 (Boc $\underline{C}(CH_3)_3$), 79.0 (C≡$\underline{C}$H), 72.1 ($\underline{C}$≡CH), 71.3 (2-$\underline{C}$H), 38.9 (Piv $\underline{C}(CH_3)_3$), 36.4 (3-$\underline{C}H_2$), 32.3 (4-$\underline{C}H_2$), 29.2 ($H_2\underline{C}$C≡C), 28.5 (Boc C($\underline{C}H_3$)$_3$), 27.1 (Piv C($\underline{C}H_3$)$_3$).

HRMS (ESI-TOF) m/z: [M + Na]$^+$ Calcd for $C_{17}H_{28}N_2O_5Na$ 363.1890; Found 363.1889 Δ = −0.28 ppm.

4-Amino-1-oxo-1-(prop-2-yn-1-ylamino)butan-2-yl pivalate hydrochloride **3**

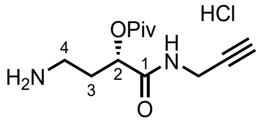

HCl in 1,4-dioxane (4 M, 3 mL) was added to amide **S6** (300 mg, 0.881 mmol) and stirred at 25 °C for 3 hours. The solvent was evaporated under reduced pressure to yield the title compound as an orange solid, which was carried forward without further purification (244 mg, 0.881 mmol, >99% yield).

$[\alpha]_D^{20}$ −37.96 (*c* 0.5, MeOH)

FTIR (neat) cm^{-1}: 3389 (m, NH stretch), 3268 (m, NH stretch), 1631 (s, CO stretch), 1623 (s, CO stretch).

^{1}H NMR (501 MHz, D_2O) δ 5.04 (dd, *J* = 12.5, 6.4 Hz, 1H, 2-C$\underline{H}$), 4.05 – 4.00 (m, 2H, $\underline{H}_2$CC≡C), 3.24 – 3.15 (m, 2H, 4-C$\underline{H}_2$), 2.69 – 2.62 (m, 1H, C≡C$\underline{H}$), 2.31 – 2.22 (m, 2H, 3-C$\underline{H}_2$), 1.26 (s, 9H, C(C$\underline{H}_3$)$_3$).

^{13}C NMR (126 MHz, D_2O) δ 180.3 (ester $\underline{C}$=O), 171.3 (1-$\underline{C}$=O), 79.1 (C≡$\underline{C}$H), 72.2 ($\underline{C}$≡CH), 71.7 (2-$\underline{C}$H), 38.6 ($\underline{C}(CH_3)_3$), 36.0 (4-$\underline{C}H_2$), 28.8 ($H_2\underline{C}$C≡C), 26.3 (C($\underline{C}H_3$)$_3$).

HRMS (ESI-TOF) m/z: [M + H]$^+$ Calcd for $C_{12}H_{21}N_2O_3$ 241.1547; Found 241.1548 Δ = 0.41 ppm.

4-Formamido-1-oxo-1-(prop-2-yn-1-ylamino)butan-2-yl pivalate **S7**

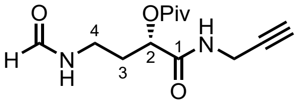

To a solution of hydrochloride salt **3** (50 mg, 0.18 mmol, 1 equiv) in ethyl formate (3 mL), *N*,*N*-diisopropylethylamine (55 µL, 0.41 mmol, 2.25 equiv) was added dropwise. The reaction mixture was

refluxed for 4 hours, and the volatiles were removed under reduced pressure. The crude mixture was purified by flash column chromatography (50 – 100% EtOAc/hexane) to yield the title compound as a colourless oil (34 mg, 0.13 mmol, 71% yield).

R_f = 0.23 (EtOAc)

$[\alpha]_D^{20}$ −16.00 (*c* 0.5, MeOH)

FTIR (neat) cm^{-1}: 3278 (m, br, NH, stretch), 1730 (m, CO stretch), 1662 (s, CO stretch).

^{1}H NMR (501 MHz, $CDCl_3$) δ 8.17 (s, 1H, C$\underline{H}$O), 6.42 (s, br, 1H, amide NH), 6.00 (s, br, 1H, formamide NH), 5.19 (dd, *J* = 6.7, 5.7 Hz, 1H, 2-C$\underline{H}$), 4.07 (ddd, *J* = 17.2, 5.2, 2.6 Hz, 1H, $\underline{H}_{2A}$CC≡C), 4.03 (ddd, *J* = 17.2, 5.2, 2.6 Hz, 1H, $\underline{H}_{2B}$CC≡C), 3.55 (dddd, *J* = 13.5, 7.1, 6.7, 5.7 Hz, 1H, 4-C$\underline{H}_{2A}$), 3.25 (dddd, *J* = 13.5, 7.7, 5.7, 5.3 Hz, 1H, 4-C$\underline{H}_{2B}$), 2.26 (app. t, *J* = 2.6 Hz, 1H, C≡C$\underline{H}$), 2.13 (app. ddt, *J* = 14.4, 7.7, 5.7, 1H, 3-C$\underline{H}_{2A}$), 2.03 (app. dtd, *J* = 14.4, 6.7, 5.3, 1H, 3-C$\underline{H}_{2B}$), 1.28 (s, 9H, C(C$\underline{H}_3$)$_3$).

^{13}C NMR (126 MHz, $CDCl_3$) δ 177.4 (ester $\underline{C}$=O), 169.6 (1-$\underline{C}$=O), 161.6 (formyl $\underline{C}$=O), 78.8 ($H_2\underline{C}$C≡C), 72.2 ($\underline{C}$≡CH), 71.2 (2-$\underline{C}$H), 39.0 ($\underline{C}$(CH_3)$_3$), 33.9 (3-$\underline{C}H_2$), 31.8 (4-$\underline{C}H_2$), 29.3 (C≡$\underline{C}$H), 27.2 (C($\underline{C}H_3$)$_3$).

HRMS (ESI-TOF) m/z: [M + H]$^+$ Calcd for $C_{13}H_{21}N_2O_4$ 269.1496; Found 269.1496 Δ = −0.00 ppm.

4-Isocyano-1-oxo-1-(prop-2-yn-1-ylamino)butan-2-yl pivalate **9**

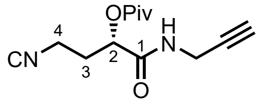

To a solution of formamide **S7** (133 mg, 0.50 mmol, 1.0 equiv) in MeCN (5 mL), a solution of *p*-toluenesulfonyl chloride (380 mg, 1.98 mmol, 4.0 equiv) in pyridine (2 mL) was added dropwise at 0 °C. The reaction mixture was stirred at 0 °C for 2 hours. The reaction was warmed to 25 °C, quenched with H_2O (10 mL), saturated with NaCl, and extracted with EtOAc (3 × 30 mL).[2] The organic layer was dried over Na_2SO_4, filtered, and the solvent was removed under reduced pressure. The crude mixture was purified by flash column chromatography (0 – 50% EtOAc/hexane) to yield the title compound as a colourless oil (77 mg, 0.31 mmol, 62% yield).

R_f = 0.74 (EtOAc)

$[\alpha]_D^{20}$ −28.00 (*c* 0.5, MeOH)

FTIR (neat) cm^{-1}: 3302 (s, CH stretch), 2150 (m, CN stretch), 1734 (s, CO stretch), 1672 (s, CO stretch).

^{1}H NMR (501 MHz, $CDCl_3$) δ 6.25 (s, 1H, N$\underline{H}$), 5.27 (dd, *J* = 7.1, 5.0 Hz, 1H, 2-C$\underline{H}$), 4.07 (app. dt, *J* = 5.0, 2.3 Hz, 2H, $\underline{H}_2$CC≡C), 3.52 (app. t, *J* = 7.0 Hz, 2H, 4-C$\underline{H}_2$), 2.42 – 2.17 (m, 3H, 3-C$\underline{H}_2$, C≡C$\underline{H}$), 1.29 (s, 9H, C(C$\underline{H}_3$)$_3$).

---

[2] Using a commercially available cyanide test kit (Supplier: Merck, Catalogue No.: 1.10044.0001), the aqueous layer was determined to contain <1 mg/L cyanide.

^{13}C NMR (126 MHz, $CDCl_3$) δ 177.2 (ester C=O), 168.3 (1-C=O), 157.9 (3 lines 1:1:1, $^{1}J_{C^{14}N}$ = 4.9 Hz, N≡C), 78.7 (C≡CH), 72.4 (C≡CH), 70.4 (2-CH), 39.1 (C(CH3)3), 37.7 (3 lines 1:1:1, $^{1}J_{C^{14}N}$ = 6.5 Hz, 4-CH_2), 31.2 (3-CH_2), 29.4 (H_2CC≡C), 27.2 ($C(CH_3)_3$).
HRMS (ESI-TOF) m/z: $[M + Na]^+$ Calcd for $C_{13}H_{18}N_2NaO_3$ 273.1206; Found 273.1210 Δ = 1.46 ppm.

Synthesis of 10

4-Ethynylbenzaldehyde[7] **5**

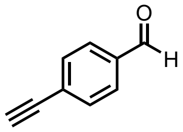

4-Bromobenzaldehyde (1.00 g, 5.40 mmol, 1.0 equiv), trimethylsilylacetylene (0.83 mL, 5.95 mmol, 1.1 equiv) and triethylamine (2.2 mL) were dissolved in THF (2 mL), and the solution was degassed *via* inert gas bubbling for 30 minutes. $Pd(PPh_3)_2Cl_2$ (38 mg, 0.05 mmol, 0.01 equiv) and CuI (10 mg, 0.05 mmol, 0.1 equiv) were dissolved in THF (1.2 mL) and degassed *via* inert gas bubbling for 30 minutes. The solution of $Pd(PPh_3)_2Cl_2$ and CuI was added to the solution containing 4-bromobenzaldehyde and was stirred at 65 °C for 16 hours. The volatiles were removed under reduced pressure and the solid was resuspended in Et_2O (10 mL), and filtered through a silica plug. The filtrate was concentrated under reduced pressure to produce a brown oil. The crude oil was dissolved in MeOH (5 mL), and K_2CO_3 (50 mg) was added to the solution. The reaction was stirred at 25 °C for 2 hours. The solvent was removed under reduced pressure, redissolved in CH_2Cl_2 (20 mL), and was filtered through a silica pad. The filtrate was concentrated under reduced pressure to produce a yellow solid (650 mg, 5.0 mmol, 92% yield) that was taken forward to the next step without further purification.

^{1}H NMR (501 MHz, $CDCl_3$) δ 10.02 (s, 1H, CHO), 7.85 (d, J = 8.4 Hz, 2H, ArH), 7.64 (d, J = 8.4 Hz, 2H, ArH), 3.29 (s, 1H, C≡CH).
Analytical data corresponds with that reported.[8]

(±)-*tert*-Butyl ((4-ethynylphenyl)(phenylsulfonyl)methyl)carbamate[9] **S8**

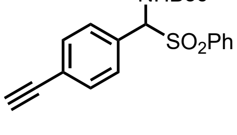

Benzaldehyde **5** (550 mg, 4.23 mmol, 1.0 equiv), *tert*-butylcarbamate (740 mg, 6.34 mmol, 1.5 equiv) and sodium benzene sulfinate (1.64 g, 8.45 mmol, 2.0 equiv) were dissolved in H_2O/THF (11 mL, 2:1). Formic acid (430 µL, 11.4 mmol, 2.7 equiv) was added to the mixture and was stirred at 25 °C for 72 hours, during which a precipitate formed. The reaction was filtered, and the residue was washed with H_2O (100 mL), followed by hexane (100 mL). The residue was dried *in vacuo* for 5 hours to yield

the title compound as an orange solid (1.23 g, 3.30 mmol, 78% yield), which was carried forward without further purification.

^{1}H NMR (501 MHz, $CDCl_3$) δ 7.90 (d, *J* = 7.4 Hz, 2H, ArH), 7.68 – 7.63 (m, 1H, ArH), 7.58 – 7.51 (m, 4H, ArH), 7.40 (d, *J* = 8.1 Hz, 2H, ArH), 5.92 (d, *J* = 10.8 Hz, 1H, NH), 5.70 (d, *J* = 10.8 Hz, 1H, CH), 3.15 (s, 1H, C≡CH), 1.26 (s, 9H, $(CH_3)_3$).

tert-Butyl (4-ethynylbenzylidene)carbamate [9] **6**

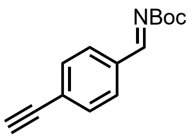

The crude carbamate **S8** (949 mg, 2.55 mmol, 1.0 equiv), K_2CO_3 (2.12 g, 15.3 mmol, 6.0 equiv) and Na_2SO_4 (2.61 g, 18.4 mmol, 7.2 equiv) were suspended in THF (15 mL), and the mixture was refluxed for 16 hours. The suspension was filtered through alternating layers of celite, Na_2SO_4 and celite, and the filter cake was washed with THF. The filtrate was concentrated to yield the title compound as a brown oil (586 mg, 2.55 mmol, >99% yield), which was carried forward without further purification.

FTIR (neat) cm^{-1}: 1688 (s, CO stretch), 1674 (s, CN stretch).
^{1}H NMR (501 MHz, $CDCl_3$) δ 8.84 (s, 1H, CHN), 7.87 (d, *J* = 8.4 Hz, 2H, 2-ArH), 7.58 (d, *J* = 8.4 Hz, 2H, 3-ArH), 3.26 (s, 1H, C≡CH), 1.59 (s, 9H, $(CH_3)_3$).
^{13}C NMR (126 MHz, $CDCl_3$) δ 168.7 (C=O), 162.6 (C=N), 134.3 (4-ArC), 132.7 (3-ArCH), 130.1 (2-ArCH), 127.4 (1-ArC), 83.0 ($C(CH_3)_3$), 82.7 (C≡CH), 80.7 (C≡CH), 28.1 ($(CH_3)_3$).

tert-Butyl (*R*)-(1-(4-ethynylphenyl)-3-hydroxypropyl)carbamate [10,11] **7**

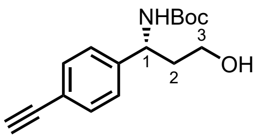

To a solution of imine **6** (1.33 g, 5.78 mmol, 1.0 equiv) in MeCN (10 mL), acetaldehyde in MeCN (15.6 mL, 0.74 M, 11.57 mmol, 2.0 equiv) was added at 0 °C. *L*-Proline (130 mg, 1.16 mmol, 0.2 equiv) was added to the mixture and the reaction was stirred at 0 °C for 3 hours. The reaction was quenched with H_2O (10 mL) and extracted with EtOAc (3 × 20 mL). The combined organic layer was washed with brine (3 × 50 mL), dried (Na_2SO_4), filtered, and the solvent was removed under reduced pressure. The major impurities were removed with a silica plug (30% EtOAc/hexane) to yield a white solid, which was carried forward without further purification (520 mg, 33% crude yield).

To a solution of crude *tert*-butyl (*R*)-(1-(4-ethynylphenyl)-3-oxopropyl)carbamate (520 mg, 1.91 mmol, 1.0 equiv) in MeOH (17 mL), $NaBH_4$ (145 mg, 3.83 mmol, 2.0 equiv) was added at 0 °C and the reaction was stirred at 25 °C for 2 hours. The reaction was quenched with H_2O (30 mL) and extracted

with EtOAc (3 × 50 mL). The combined organic layer was washed with brine (3 × 100 mL), dried (Na_2SO_4), filtered, and the solvent was removed under reduced pressure. The crude material was purified *via* flash column chromatography (0 – 50% EtOAc/hexane) to yield the title compound as a colourless oil (343 mg, 1.25 mmol, 21% yield over two steps).

R_f = 0.31 (50% EtOAc/hexane)

$[\alpha]_D^{20}$ −40.00 (*c* 0.5, MeOH)

FTIR (neat) cm^{-1}: 3292 (m, br, OH stretch), 2976 (m, NH stretch), 1687 (s, CO stretch).

^{1}H NMR (501 MHz, $CDCl_3$) δ 7.47 (d, *J* = 8.2 Hz, 2H, 3-ArH), 7.26 (d, *J* = 8.2 Hz, 1H, 2-ArH), 5.07 (d, *J* = 8.2 Hz, 1H, 1-CH), 4.92 – 4.88 (m, 1H, NH), 3.70 – 3.67 (m, 2H, 3-CH_2), 3.07 (s, 1H, C≡CH), 2.85 (s, 1H, OH), 2.11 – 1.99 (m, 1H, 2-CH_{2A}), 1.86 – 1.75 (m, 1H, 2-CH_{2B}), 1.44 (s, 9H, $(CH_3)_3$).

^{13}C NMR (126 MHz, $CDCl_3$) δ 156.4 (C=O), 143.0 (1-ArC), 132.7 (3-ArCH), 126.5 (2-ArCH), 121.4 (4-ArC), 83.4 (C≡CH), 80.3 ($C(CH_3)_3$), 77.4 (C≡CH), 59.2 (3-CH_2), 51.6 (1-CH), 39.2 (2-CH_2), 28.5 ($(CH_3)_3$).

HRMS (ESI-TOF) m/z: $[M + Na]^+$ Calcd for $C_{16}H_{21}NNaO_3$ 298.1414; Found 298.1408 Δ = −2.01 ppm.

(*R*)-3-((*tert*-Butoxycarbonyl)amino)-3-(4-ethynylphenyl)propyl pivalate **S9**

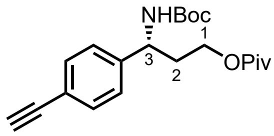

To a solution of amino alcohol **7** (343 mg, 1.25 mmol, 1.0 equiv) in CH_2Cl_2 (2.3 mL), a solution of triethylamine (440 μL, 3.11 mmol, 2.5 equiv) and 4-dimethylaminopyridine (15 mg, 0.12 mmol, 0.1 equiv) in CH_2Cl_2 (5.4 mL) was added. A solution of pivaloyl chloride (260 μL, 2.12 mmol, 1.7 equiv) in CH_2Cl_2 (5.2 mL) was added to the mixture dropwise. The reaction was stirred at 25 °C for 16 h. The reaction was diluted with CH_2Cl_2 (50 mL), washed with saturated $NaHCO_3$ (3 × 50 mL), saturated NH_4Cl (3 × 50 mL), and brine (3 × 50 mL). The organic layer was dried (Na_2SO_4), filtered, and the solvent was removed under reduced pressure. The crude material was purified *via* flash column chromatography (30% EtOAc/hexane) to yield the title compound as a colourless oil (406 mg, 1.13 mmol, 90% yield).

R_f = 0.61 (50% EtOAc/hexane)

$[\alpha]_D^{20}$ −24.00 (*c* 0.5, MeOH)

FTIR (neat) cm^{-1}: 3297 (m, br, NH stretch), 1701 (m, CO stretch), 1696 (s, CO stretch), 1166 (s, CO stretch).

^{1}H NMR (501 MHz, $CDCl_3$) δ 7.46 (d, *J* = 8.2 Hz, 2H, 3-ArH), 7.22 (d, *J* = 8.1 Hz, 2H, 2-ArH), 5.03 (s, 1H, NH), 4.81 – 4.77 (m, 1H, 3-CH), 4.09 – 3.99 (m, 2H, 1-CH_2), 3.06 (s, 1H, C≡CH), 2.10 – 2.03 (m, 2H, 2-CH_2), 1.40 (s, 9H, Boc $(CH_3)_3$), 1.21 (s, 9H, Piv $(CH_3)_3$).
^{13}C NMR (126 MHz, $CDCl_3$) δ 178.4 (Piv C=O), 155.1 (Boc C=O), 143.1 (4-ArC), 132.7 (3-ArCH), 126.3 (2-ArCH), 121.3 (1-ArC), 83.5 (C≡CH), 79.9 (Boc $C(CH_3)_3$), 77.4 (C≡CH), 61.5 (CH_2OPiv), 60.6 (CHNH), 38.9 (Piv $C(CH_3)_3$), 35.4 (CHNHCH_2), 28.5 (Boc $C(CH_3)_3$), 27.3 (Piv $C(CH_3)_3$).
HRMS (ESI-TOF) m/z: $[M + Na]^+$ Calcd for $C_{21}H_{29}NNaO_4$ 382.1989; Found 382.1984 Δ = −1.31 ppm.

(*R*)-3-Amino-3-(4-ethynylphenyl)propyl pivalate hydrogen chloride **8**

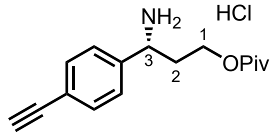

HCl in 1,4-dioxane (4 M, 5 mL) was added to carbamate **S9** (406 mg, 1.13 mmol) and stirred at 25 °C for 2 h. The solvent was removed under reduced pressure to yield the title compound as a white solid, which was carried forward without further purification (274 mg, 0.93 mmol, 82% yield).

R_f = 0 (100% EtOAc)
$[\alpha]_D^{20}$ +64.00 (*c* 0.5, MeOH)
FTIR (neat) cm^{-1}: 2971 (br, NH stretch), 1726 (m, CO stretch), 1161 (s, CO stretch).
^{1}H NMR (501 MHz, $CDCl_3$) δ 8.88 (s, 3H, NH_3^+), 7.50 (d, *J* = 8.0 Hz, 2H, 3-ArH), 7.41 (d, *J* = 8.0 Hz, 2H, 2-ArH), 4.33 (s, 1H, 3-CH), 4.08 (app. dt, *J* = 11.5, 5.3 Hz, 1H, 1-CH_{2A}), 3.82 (ddd, *J* = 11.5, 7.9, 4.9 Hz, 1H, 1-CH_{2B}), 3.13 (s, 1H, C≡CH), 2.53 – 2.37 (m, 1H, 2-CH_{2A}), 2.35 – 2.19 (m, 1H, 2-CH_{2B}), 1.16 (s, 9H, Piv $(CH_3)_3$).
^{13}C NMR (126 MHz, $CDCl_3$) δ 178.2 (C=O), 135.7 (1-ArC), 133.2 (3-ArCH), 127.7 (2-ArCH), 123.7 (4-ArC), 82.7 (C≡CH), 78.9 (C≡CH), 60.1 (1-CH_2), 53.6 (3-CH), 38.9 ($C(CH_3)_3$), 33.5 (2-CH_2), 27.3 ($C(CH_3)_3$).
HRMS (ESI-TOF) m/z: $[M + H]^+$ Calcd for $C_{16}H_{22}NO_2$ 260.1645; Found 260.1642 Δ = −1.15 ppm.

(*R*)-3-(4-Ethynylphenyl)-3-formamidopropyl pivalate **S10**

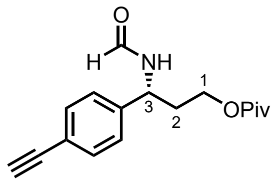

To a solution of hydrochloride salt **8** (274 mg, 0.93 mmol, 1.0 equiv) in ethyl formate (4 mL), *N*,*N*-diisopropylethylamine (360 µL, 2.08 mmol, 2.25 equiv) was added to the dropwise. The reaction was refluxed for 16 h, and the volatiles were removed under reduced pressure. The crude material was

purified *via* flash column chromatography (50 – 100% EtOAc/hexane) to yield the title compound as a colourless oil (240 mg, 0.84 mmol, 90% yield).

R_f = 0.66 (100% EtOAc)
$[\alpha]_D^{20}$ −60.00 (*c* 0.5, MeOH)
FTIR (neat) cm^{-1}: 3042 (w, NH stretch), 1723 (m, CO stretch), 1658 (s, CO stretch), 1152 (s, CO stretch).
^1H NMR (501 MHz, $CDCl_3$) δ 8.18 (s, 1H, CHO), 7.46 (d, *J* = 8.3 Hz, 2H, 3-ArH), 7.24 (d, *J* = 8.3 Hz, 2H, 2-ArH), 6.25 (d, *J* = 8.0 Hz, 1H, NH), 5.18 (app. td, *J* = 8.0, 6.9 Hz, 1H, 3-CH), 4.12 – 3.98 (m, 2H, 1-CH_2), 3.07 (s, 1H, C≡CH), 2.16 – 2.10 (m, 2H, 2-CH_2), 1.19 (s, 9H, $(CH_3)_3$).
^{13}C NMR (126 MHz, $CDCl_3$) δ 178.6 (C=O), 160.5 (CHO), 141.6 (1-ArC), 132.8 (3-ArCH), 126.5 (2-ArCH), 121.8 (4-ArC), 83.2 (C≡CH), 77.7 (C≡CH), 61.2 (1-CH_2), 49.5 (3-CH), 38.9 ($C(CH_3)_3$), 34.7 (2-CH_2), 27.3 ($C(CH_3)_3$).
HRMS (ESI-TOF) m/z: $[M + H]^+$ Calcd for $C_{17}H_{22}NO_3$ 288.1594; Found 288.1593 Δ = −0.35 ppm.

(*R*)-3-(4-Ethynylphenyl)-3-isocyanopropyl pivalate **10**

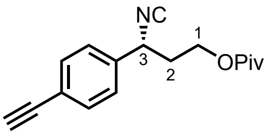

To a solution of formamide **S10** (164 mg, 0.57 mmol, 1.0 equiv) in MeCN (1.8 mL), 4-toluenesulfonyl chloride (135 mg, 0.71 mmol, 4.0 equiv) in pyridine (710 µL) was added at 0 °C and was stirred at 25 °C for 16 h. The reaction was quenched with H_2O (10 mL), saturated with NaCl, and extracted with EtOAc (3 × 30 mL). The combined organic layer was dried (Na_2SO_4), filtered, and the solvent was removed under reduced pressure. The crude material was purified *via* flash column chromatography (50% EtOAc/hexane) to yield the title compound as a colourless oil (97 mg, 0.36 mmol, 63% yield).

R_f = 0.79 (100% EtOAc)
$[\alpha]_D^{20}$ −8.00 (*c* 0.5, MeOH)
FTIR (neat) cm^{-1}: 2138 (m, CN stretch), 1723 (m, CO stretch), 1150 (s, CO stretch).
^1H NMR (501 MHz, $CDCl_3$) δ 7.53 (d, *J* = 8.3 Hz, 2H, 3-ArH), 7.31 (d, *J* = 8.3 Hz, 2H, 2-ArH), 4.82 (dd, *J* = 8.7, 5.5 Hz, 1H, 3-CH), 4.23 (ddd, *J* = 11.5, 7.9, 5.0 Hz, 1H, 1-CH_{2A}), 4.16 (app. dt, *J* = 11.5, 5.6 Hz, 1H, 1-CH_{2B}), 3.11 (s, 1H, C≡CH), 2.28 – 2.13 (m, 2H, 2-CH_2), 1.22 (s, 9H, $(CH_3)_3$).
^{13}C NMR (126 MHz, $CDCl_3$) δ 178.3 (C=O), 158.7 (3 lines 1:1:1, $^1J_{C^{14}N}$ = 3.4 Hz, NC), 137.2 (1-ArC), 133.0 (3-ArCH), 126.0 (2-ArCH), 122.9 (4-ArC), 82.8 (C≡CH), 78.4 (C≡CH), 60.2 (1-CH_2), 55.6 (3 lines 1:1:1, $^1J_{C^{14}N}$ = 5.6 Hz, 3-CH), 39.0 ($C(CH_3)_3$), 37.6 (2-CH_2), 27.3 ($C(CH_3)_3$).
HRMS (ESI-TOF) m/z: $[M + Na]^+$ Calcd for $C_{17}H_{19}NNaO_2$ 292.1308; Found 292.1308 Δ = −0.00 ppm.

Synthesis of 17

tert-Butyl (2-formamidoethyl)carbamate[12] **S11**

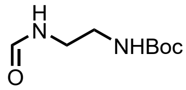

tert-Butyl (2-aminoethyl)carbamate **15** (1.00 mL, 6.25 mmol) was dissolved in ethyl formate (10 mL) and was refluxed for 16 hours. The solvent was removed under reduced pressure to yield the title compound as a colourless oil, which was carried forward without further purification (1.17 g, 6.25 mmol, >99% yield)

^{1}H NMR (501 MHz, $CDCl_3$) δ 8.18 (s, 1H, C<u>H</u>O), 6.35 (s, 1H, N<u>H</u>), 4.94 (s, 1H, N<u>H</u>), 3.41 (app. q, *J* = 5.6 Hz, 2H, C<u>H</u>$_2$), 3.29 (app. q, *J* = 5.7 Hz, 2H, C<u>H</u>$_2$), 1.44 (s, 9H, C(C<u>H</u>$_3$)$_3$).
Analytical data corresponds to that reported in the literature.[13]

N-(2-Aminoethyl)formamide hydrochloride **14**

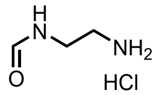

HCl in 1,4-dioxane (4 M, 10 mL) was added to carbamate **S11** (1.17 g, 6.25 mmol). The reaction was stirred at 25 °C for 2 hours. The volatiles were removed under reduced pressure to yield the title compound as a white solid, which was carried forward without further purification (1.26 g, 6.25 mmol, >99% yield).

FTIR (neat) cm^{-1}: 3230 (s, NH stretch), 2964 (s, NH stretch), 1654 (m, CO stretch).
^{1}H NMR (501 MHz, D_2O) δ 8.16 (s, 1H, C<u>H</u>O), 3.57 (t, *J* = 6.0 Hz, 2H, C<u>H</u>$_2$), 3.19 (t, *J* = 6.0 Hz, 2H, C<u>H</u>$_2$).

2,5-Dioxopyrrolidin-1-yl 3',6'-bis(dimethylamino)-3-oxo-3*H*-spiro[isobenzofuran-1,9'-xanthene]-5-carboxylate[14] **S12**

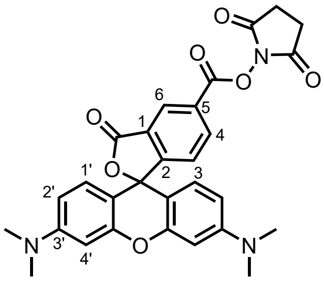

To a solution of 5-carboxytetramethylrhodamine **15** (50 mg, 0.11 mmol, 1.0 equiv) in DMF (4.5 mL), *N*-hydroxysuccinimide (13 mg, 0.11 mmol, 1.0 equiv), and 1-ethyl-3-(3-dimethylaminopropyl)carbodiimide (66 mg, 0.34 mmol, 3.0 equiv) were added. *N,N*-

Diisopropylethylamine (175 µL, 0.34 mmol, 3.0 equiv) was added to the reaction and the mixture was stirred at 25 °C for 16 hours. The reaction was diluted with EtOAc (50 mL) and was washed with aqueous NaH_2PO_4 (3 × 50 mL) and brine (3 × 50 mL). The combined organic layer was dried (Na_2SO_4), filtered, and the solvent was removed under reduced pressure to yield the title compound as a purple solid, which was carried forward without further purification (50 mg, 0.10 mmol, 87% yield).

FTIR (neat) cm^{-1}: 1769 (m, CO stretch), 1739 (s, CO stretch), 1613 (s, CO stretch).
^{1}H NMR (501 MHz, $CDCl_3$) δ 8.78 (dd, *J* = 1.5, 0.7 Hz, 1H, 6-ArH), 8.35 (dd, *J* = 8.0, 1.5 Hz, 1H, 4-ArH), 7.32 (dd, *J* = 8.0, 0.7 Hz, 1H, 3-ArH), 6.55 (d, *J* = 8.9 Hz, 2H, 1'-ArH), 6.48 (d, *J* = 2.6 Hz, 2H, 4'-ArH), 6.40 (dd, *J* = 8.9, 2.6 Hz, 2H, 2'-ArH), 2.98 (s, 12H, $N(CH_3)_2$), 2.93 (s, 4H, CH_2).
^{13}C NMR (126 MHz, $CDCl_3$) δ 169.1 (NHS C=O), 168.2 (COOR), 160.9 (COON), 153.0 (ArC-C1'), 152.4 (3'-ArC), 136.3 (2-ArC), 128.8 (4-ArCH), 128.6 (1'-ArCH), 127.8 (3-ArCH), 126.8 (5-ArC), 125.2 (6-ArCH), 109.0 (2'-ArCH), 105.5 (ArC-C4'), 98.6 (4'-ArCH), 40.4 ($N(CH_3)_2$), 25.8 (CH_2).
HRMS (ESI-TOF) m/z: $[M + Na]^+$ Calcd for $C_{29}H_{25}N_3NaO_7$ 550.1585; Found 550.1580 Δ = −0.91 ppm.

3',6'-*Bis*(dimethylamino)-*N*-(2-formamidoethyl)-3-oxo-3*H*-spiro[isobenzofuran-1,9'-xanthene]-5-carboxamide **16**

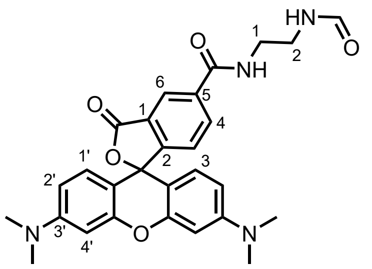

To a solution of NHS ester **S12** (29 mg, 0.05 mmol, 1.0 equiv) and triethylamine (91 µL, 0.66 mmol, 12.0 equiv) in MeCN (3 mL), hydrochloride salt **14** (41 mg, 0.33 mmol, 6.0 equiv) was added at 0 °C. The reaction was stirred at 0 °C for 8 hours, and the volatiles were removed under reduced pressure. The crude material was purified *via* flash column chromatography (10 – 50% MeOH/CH_2Cl_2) to yield the title compound as a purple solid (27 mg, 0.05 mmol, 93% yield).

R_f = 0.68 (50% MeOH/CH_2Cl_2)
FTIR (neat) cm^{-1}: 3290 (br, NH stretch), 1721 (m, CO stretch), 1645 (m, CO stretch), 1595 (m, CC stretch).
^{1}H NMR (501 MHz, CD_3OD) δ 8.54 (d, *J* = 1.8 Hz, 1H, 6-ArH), 8.09 (s, 1H, CHO), 8.04 (dd, *J* = 7.9, 1.8 Hz, 1H, 4-ArH), 7.34 (d, *J* = 7.9 Hz, 1H, 3- ArH), 7.22 (d, *J* = 9.5 Hz, 2H, 1'-ArH), 6.99 (dd, *J* = 9.5, 2.5 Hz, 2H, 2'-ArH), 6.88 (d, *J* = 2.5 Hz, 2H, 4'-ArH), 3.56 (t, *J* = 6.1 Hz, 2H, 1-CH_2), 3.49 (t, *J* = 6.1 Hz, 2H, 2-CH_2), 3.26 (s, 12H, $N(CH_3)_2$).
^{13}C NMR (126 MHz, CD_3OD) δ 169.3 (NHCO), 167.4 (COOR), 164.3 (CHO), 159.0 (ArC-C1'), 158.6 (3'-ArC), 142.1 (1-ArC), 137.0 (2-ArC), 137.0 (5-ArC), 132.5 (1'-ArCH), 130.8 (3-ArCH), 129.6 (6-

ArCH), 129.5 (4-ArCH), 115.0 (ArC-C4’), 114.7 (2’-ArCH), 97.4 (4’-ArCH), 47.6 (ArC2-C), 40.8 ($N(CH_3)_2$), 40.7 (1-CH_2), 38.6 (2-CH_2).
HRMS (ESI-TOF) m/z: $[M + H]^+$ Calcd for $C_{28}H_{28}N_4O_5$ 501.2132; Found 501.2132 Δ = 0.00 ppm.

3',6'-*Bis*(dimethylamino)-*N*-(2-isocyanoethyl)-3-oxo-3*H*-spiro[isobenzofuran-1,9'-xanthene]-5-carboxamide **17**

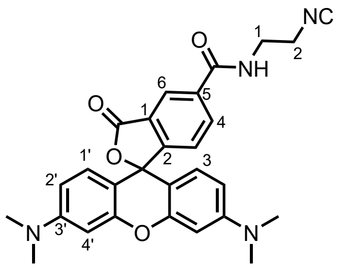

To a solution of formamide **16** (28 mg, 0.06 mmol, 1.0 equiv) in MeCN (5 mL), Burgess reagent (20 mg, 0.08 mmol, 1.5 equiv) was added. The reaction was stirred at 25 °C for 16 hours, and the volatiles were removed under reduced pressure. The crude material was purified *via* flash column chromatography (20% MeOH/CH_2Cl_2) to yield the title compound as a purple solid (17 mg, 0.04 mmol, 63% yield).

R_f = 0.34 (20% MeOH/CH_2Cl_2)
FTIR (neat) cm^{-1}: 3263 (br, NH stretch), 2162 (w, CN stretch), 1741 (m, C=O stretch), 1596 (s, C=O stretch).
^{1}H NMR (501 MHz, CD_3OD) δ 8.55 (d, *J* = 1.8 Hz, 1H, 6-ArH), 8.08 (dd, *J* = 7.9, 1.8 Hz, 1H, 4-ArH), 7.38 (d, *J* = 7.9 Hz, 1H, 3-ArH), 7.26 (d, *J* = 9.5 Hz, 2H, 1’-ArH), 7.02 (dd, *J* = 9.5, 2.5 Hz, 2H, 2’-ArH), 6.92 (d, *J* = 2.5 Hz, 2H, 4’-ArH), 3.79 (t, *J* = 6.0 Hz, 2H, 1-CH_2), 3.72 (t, *J* = 6.0 Hz, 2H, 2-CH_2), 3.28 (s, 12H, $N(CH_3)_2$).
^{13}C NMR (126 MHz, CD_3OD) δ 169.5 (NHCO), 161.9 (COOR), 159.1 (ArC-C1’), 158.7 (ArC-C4’), 157.5 (NC[3]), 142.3 (1-ArC), 137.2 (2-ArC), 136.6 (5-ArC), 132.6 (1’-ArCH), 130.9 (3-ArCH), 129.5 (4-ArCH), 129.5 (6-ArCH), 115.0 (2’-ArCH), 114.8 (3’-ArC), 97.3 (4’-ArCH), 53.6 (C-ArC2), 41.9 (3 lines 1:1:1, $^{1}J_{C^{14}N}$ = 6.9 Hz, 2-CH_2), 40.8 (1-CH_2), 40.4 (CH_2).
HRMS (ESI-TOF) m/z: $[M + H]^+$ Calcd for $C_{28}H_{27}N_4O_4$ 483.2027; Found 483.2013 Δ = −2.90 ppm.

Synthesis of 12

1-(4-Ethynylphenyl)ethan-1-one[15] **S4**

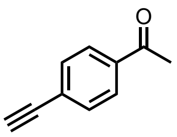

[3] C–^{14}N coupling was not observed

1-(4-Iodophenyl)ethan-1-one **S3** (1.0 g, 4.06 mmol, 1.0 equiv) and ethynyltrimethylsilane (790 μL, 5.69 mmol, 1.4 equiv) were dissolved in NEt_3 (22.7 mL) and the mixture was degassed *via* inert gas bubbling for 3 hours. $Pd(PPh_3)_2Cl_2$ (28.5 mg, 0.04 mmol, 0.01 equiv) and CuI (7.7 mg, 0.04 mmol, 0.01 equiv) were added and the reaction was stirred at 25 °C for 72 hours. The reaction was filtered through a silica plug, which was flushed with Et_2O. The filtrate was concentrated under reduced pressure to yield a yellow oil, which was carried forward without further purification.

The crude 1-(4-((trimethylsilyl)ethynyl)phenyl)ethan-1-one and excess K_2CO_3 (≈3.0 g) were suspended in MeOH (30 mL) and stirred at 25 °C for 2 hours. The solvent was removed under reduced pressure, and the resulting solid was resuspended in CH_2Cl_2 (70 mL). The suspension was filtered through a silica plug, which was flushed with CH_2Cl_2. The filtrate was concentrated under reduced pressure to yield the crude material. The crude material was purified *via* flash column chromatography (0 – 30% EtOAc/hexane) to yield the title compound as a white solid (326 mg, 2.23 mmol, 55% yield).

R_f = 0.35 (30% EtOAc/hexane)

^{1}H NMR (501 MHz, $CDCl_3$) δ 7.91 (d, *J* = 8.5 Hz, 2H, Ar<u>H</u>), 7.58 (d, *J* = 8.5 Hz, 2H, Ar<u>H</u>), 3.25 (s, 1H, C<u>H</u>), 2.60 (s, 3H, C<u>H</u>$_3$).

Analytical data corresponds with that reported in the literature.[15]

1-(4-Ethynylphenyl)prop-2-en-1-one[16] **12**

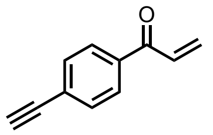

Diisopropylamine (2 mL) was dissolved in Et_2O (10 mL) and was cooled to 0 °C. Trifluoroacetic acid (1.5 mL) was added dropwise and the solution was stirred at 0 °C for 5 minutes. The reaction was filtered and the filtrate was washed with Et_2O and dried under vacuum to yield diisopropylammonium trifluoroacetate as a white solid.

To a solution of acetophenone **S4** (325 mg, 2.23 mmol, 1.0 equiv) and paraformaldehyde (135 mg, 4.47 mmol, 2.0 equiv) in THF (3.3 mL), diisopropylammonium trifluoroacetate (485 mg, 2.26 mmol, 1.0 equiv) and trifluoroacetic acid (35 μL, 0.23 mmol, 0.1 equiv) were added, and the mixture was refluxed for 2 hours. The reaction was cooled to 25 °C and paraformaldehyde (135 mg, 4.47 mmol, 2.0 equiv) was added. The mixture was refluxed for a further 20 hours. The reaction was cooled to 25 °C, and the volatiles were removed under reduced pressure. The crude material was redissolved in Et_2O (50 mL), and was washed with aqueous HCl (1 M, 3 × 50 mL), aqueous NaOH (1 M, 3 × 50 mL) and brine (3 × 50 mL). The organic layer was dried (Na_2SO_4), filtered, and the solvent was removed under reduced pressure. The crude material was purified *via* flash column chromatography (100% toluene) to yield the title compound as a yellow solid (72 mg, 0.46 mmol, 20% yield).

R_f = 0.42 (100% toluene)

^{1}H NMR (501 MHz, $CDCl_3$) δ 7.90 (d, *J* = 8.6 Hz, 2H, ArH), 7.59 (d, *J* = 8.6 Hz, 2H, ArH), 7.13 (dd, *J* = 17.1, 10.6 Hz, 1H, C=CH), 6.45 (dd, *J* = 17.1, 1.6 Hz, 1H, C=CH_{2A}), 5.96 (dd, *J* = 10.6, 1.6 Hz, 1H, C=CH_{2B}), 3.26 (s, 1H, CH).

Analytical data corresponds with that reported in the literature.[17]

Synthesis of Mosher's Amides S1 and S2

To a solution of ammonium salt (0.08 mmol, 1.0 equiv) in CH_2Cl_2 (1.0 mL), MTPA-Cl (*R* or *S*, 17.2 µL, 0.09 mmol, 1.1 equiv) and *N*,*N*-diisopropylethylamine (32 µL, 0.18 mmol, 2.2 equiv) were added, and the solution was stirred at 25 °C for 1 hour. The reaction was washed with brine (3 × 1 mL), dried (Na_2SO_4), filtered and the solvent was removed under reduced pressure to yield the crude product (95 – 99% yield). The crude material of each diastereoisomer was analysed *via* ^{1}H and ^{19}F NMR spectroscopy.

References

1 D. C. Lim, V. Joukov, T. J. Rettenmaier, A. Kumagai, W. G. Dunphy, J. A. Wells and M. B. Yaffe, *Sci. Signaling*, 2020, **13**, eabb6707.

2 S. G. Burgess and R. Bayliss, *Acta Crystallogr., Sect. F:Struct. Biol. Commun.*, 2015, **71**, 315–319.

3 J. Sherma and B. Fried, *Handbook of Thin Layer Chromatography*, Marcel Dekker Inc, New York, 1991, vol. 55.

4 W. C. Still, M. Kahn and A. Mitra, *J. Org. Chem.*, 1978, **43**, 2923–2925.

5 H. E. Gottlieb, V. Kotlyar and A. Nudelman, *J. Org. Chem.*, 1997, **62**, 7512–7515.

6 M. E. Farkas, B. C. Li, C. Dose and P. B. Dervan, *Bioorg. Med. Chem. Lett.*, 2009, **19**, 3919–3923.

7 R. M. Edkins, M. R. Probert, C. M. Robertson, J. A. K. Howard and A. Beeby, *RSC Adv.*, 2014, **4**, 5351–5356.

8 W. B. Austin, N. Bilow, W. J. Kelleghan and K. S. Y. Lau, *J. Org. Chem.*, 1981, **46**, 2280–2286.

9 E. Ronchi, S. M. Paradine and E. N. Jacobsen, *J. Am. Chem. Soc.*, 2021, **143**, 7272–7278.

10 T. Kano, Y. Yamaguchi and K. Maruoka, *Angew. Chem., Int. Ed.*, 2009, **48**, 1838–1840.

11 G. Jersovs, M. Bojars, P. A. Donets and E. Suna, *Org. Lett.*, 2022, **24**, 4625–4629.

12 W. Szymański, W. A. Velema and B. L. Feringa, *Angew. Chem., Int. Ed.*, 2014, **53**, 8682–8686.

13 P. Xu, T. Zhang, W. Wang, X. Zou, X. Zhang and Y. Fu, *Synthesis (Stuttg)*, 2003, **2003**, 1171–1176.

14 T. Terai, M. Kohno, G. Boncompain, S. Sugiyama, N. Saito, R. Fujikake, T. Ueno, T. Komatsu, K. Hanaoka, T. Okabe, Y. Urano, F. Perez and T. Nagano, *J. Am. Chem. Soc.*, 2015, **137**, 10464–10467.

15 X. Shao, X. Wang, T. Yang, L. Lu and Q. Shen, *Angew. Chem., Int. Ed.*, 2013, **52**, 3457–3460.

16 A. Bugarin, K. D. Jones and B. T. Connell, *Chem. Commun.*, 2010, **46**, 1715.

17 Y.-F. Liu, P.-Y. Ji, J.-W. Xu, Y.-Q. Hu, Q. Liu, W.-P. Luo and C.-C. Guo, *J. Org. Chem.*, 2017, **82**, 7159–7164.

NMR Spectra

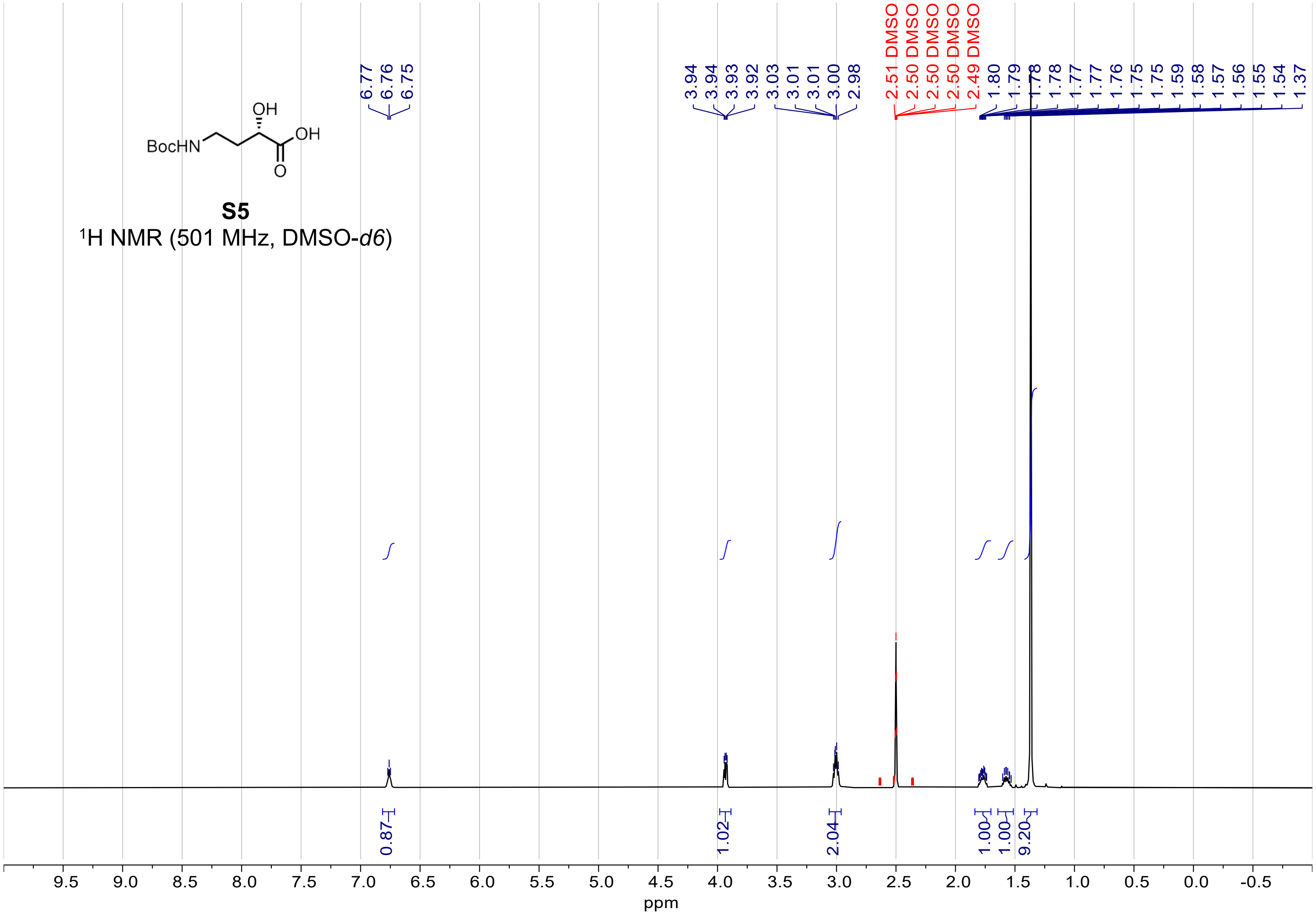

S5

^{1}H NMR (501 MHz, DMSO-*d6*)

Figure S9. *^{1}H NMR spectrum of (S)-4-((tert-butoxycarbonyl)amino)-2-hydroxybutanoic acid* ***S5****.*

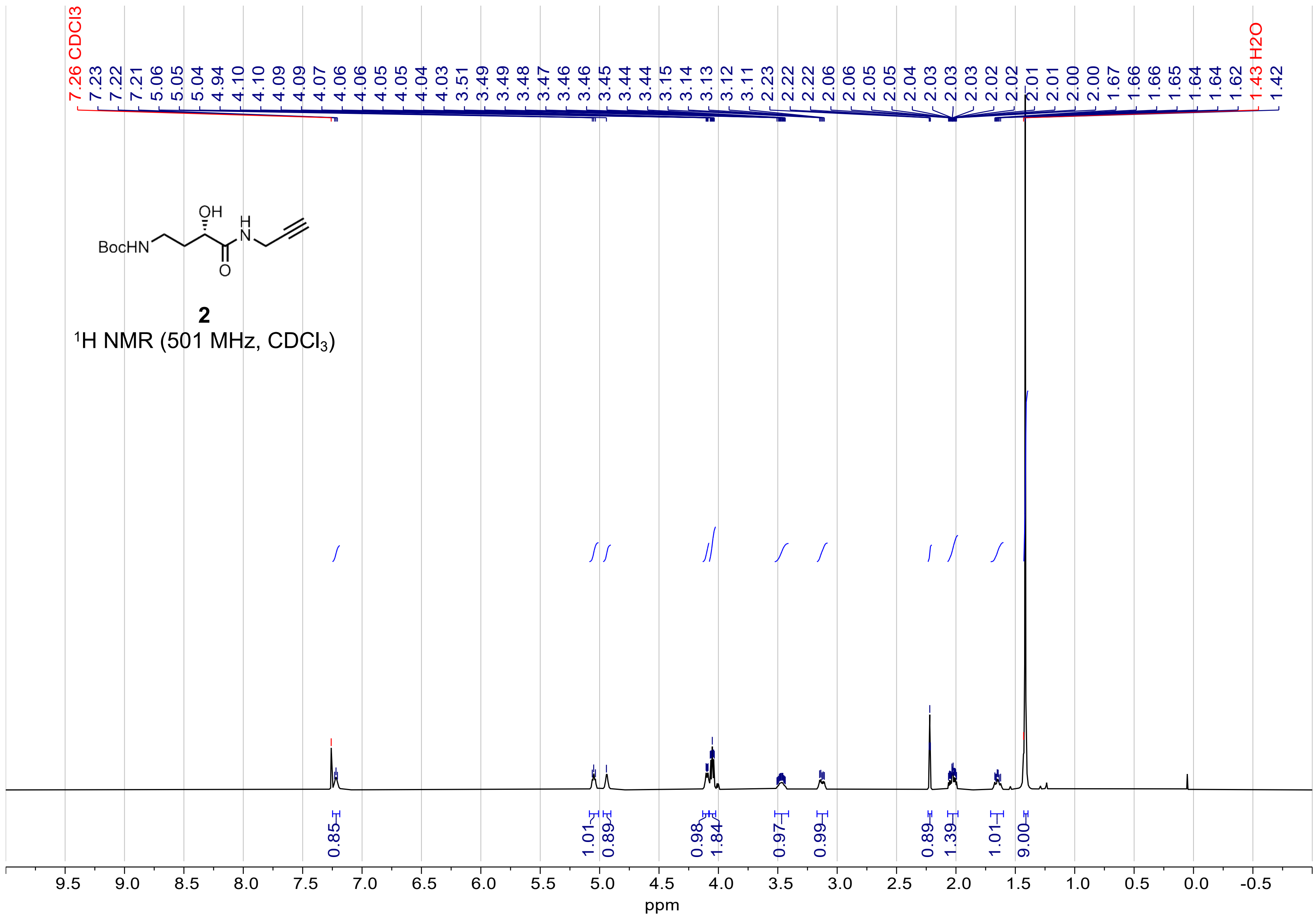

Figure S10. *1H NMR spectrum of tert-Butyl (3-hydroxy-4-oxo-4-(prop-2-yn-1-ylamino)butyl)carbamate* ***2****.*

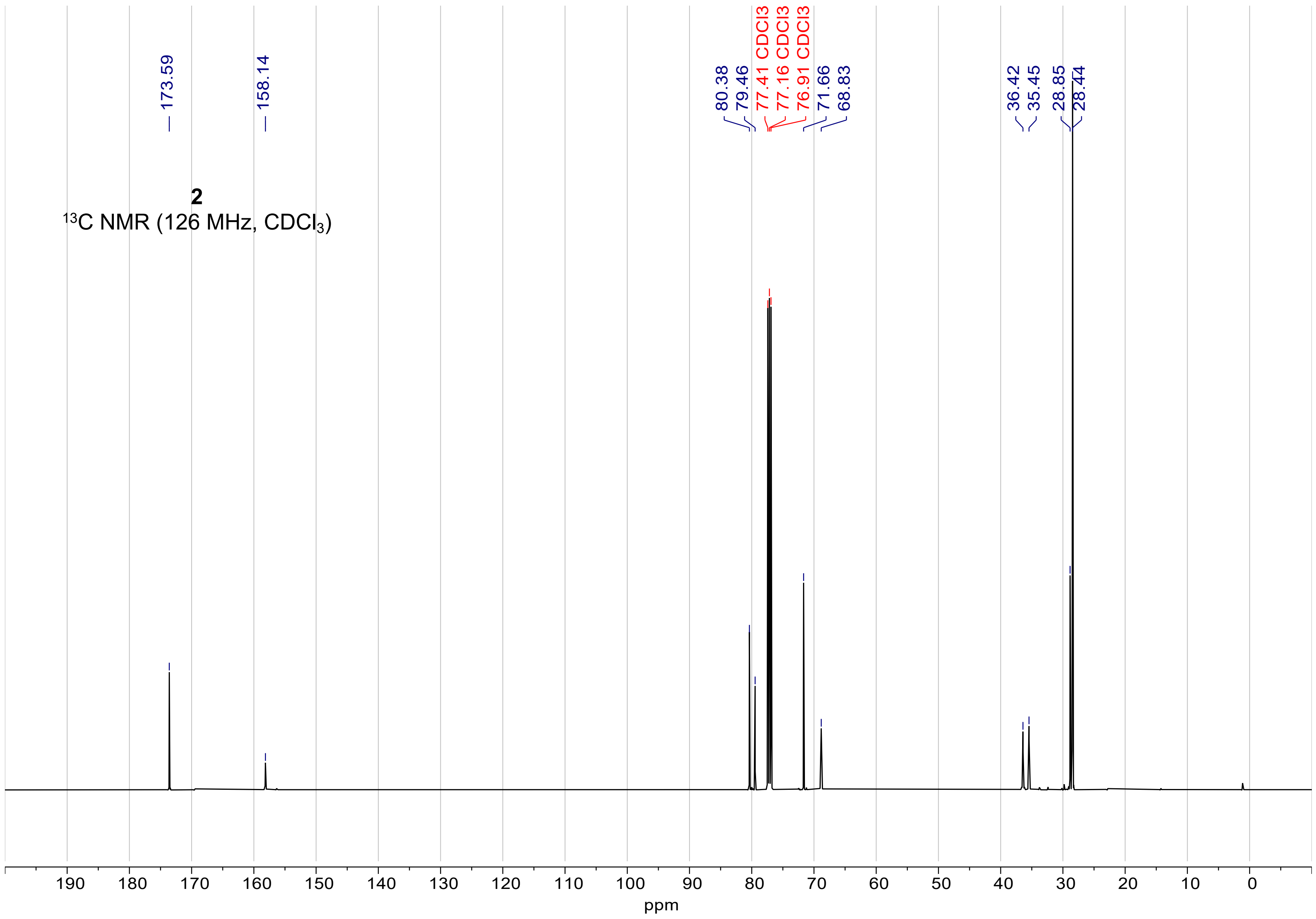

Figure S11. *^{13}C NMR spectrum of tert-Butyl (3-hydroxy-4-oxo-4-(prop-2-yn-1-ylamino)butyl)carbamate* ***2****.*

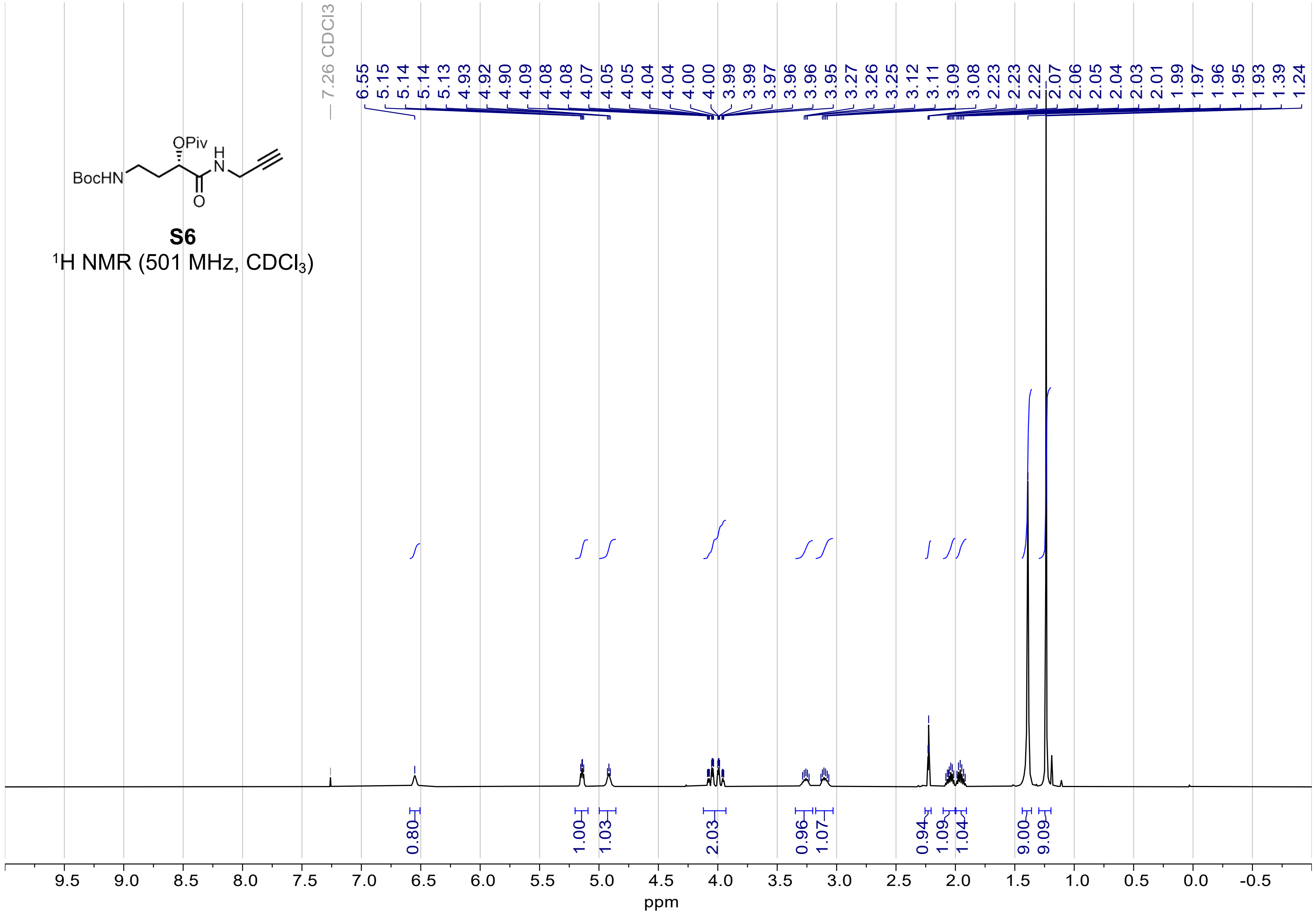

Figure S12. *^{1}H NMR spectrum of 4-((tert-butoxycarbonyl)amino)-1-oxo-1-(prop-2-yn-1-ylamino)butan-2-yl pivalate* ***S6****.*

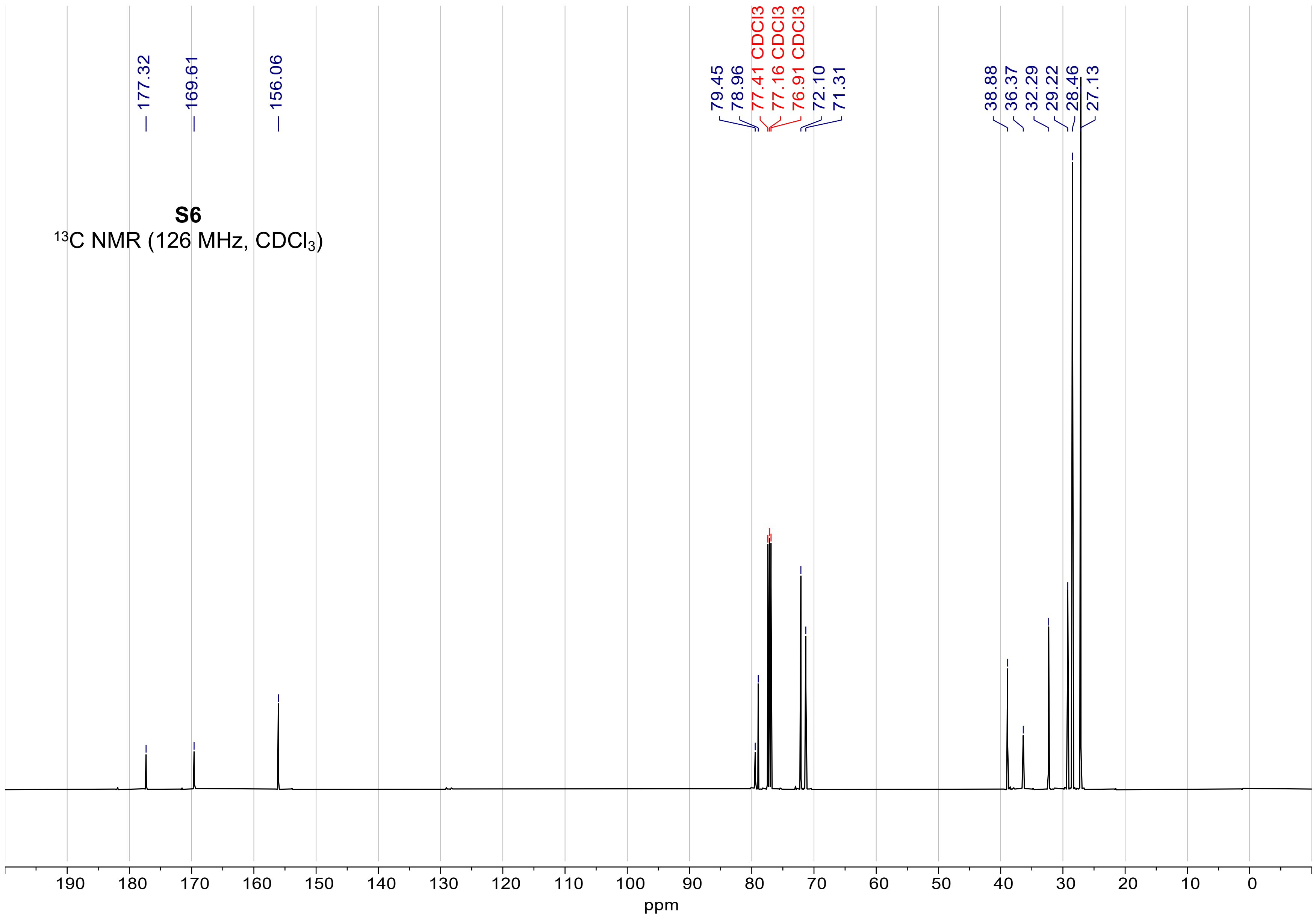

Figure S13. *^{13}C NMR spectrum of 4-((tert-butoxycarbonyl)amino)-1-oxo-1-(prop-2-yn-1-ylamino)butan-2-yl pivalate* ***S6****.*

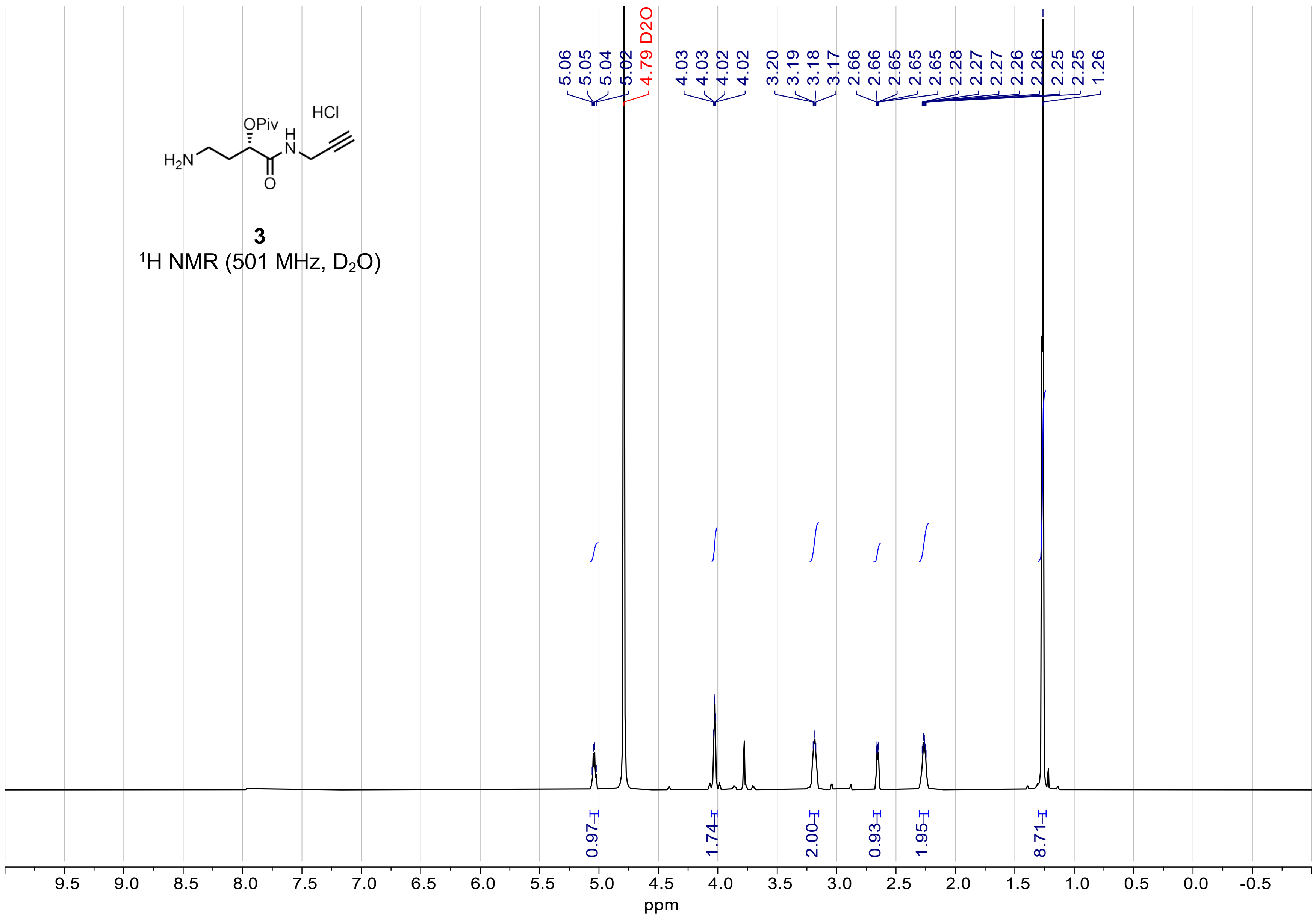

Figure S14. *^{1}H NMR spectrum of 4-amino-1-oxo-1-(prop-2-yn-1-ylamino)butan-2-yl pivalate hydrochloride* ***3****.*

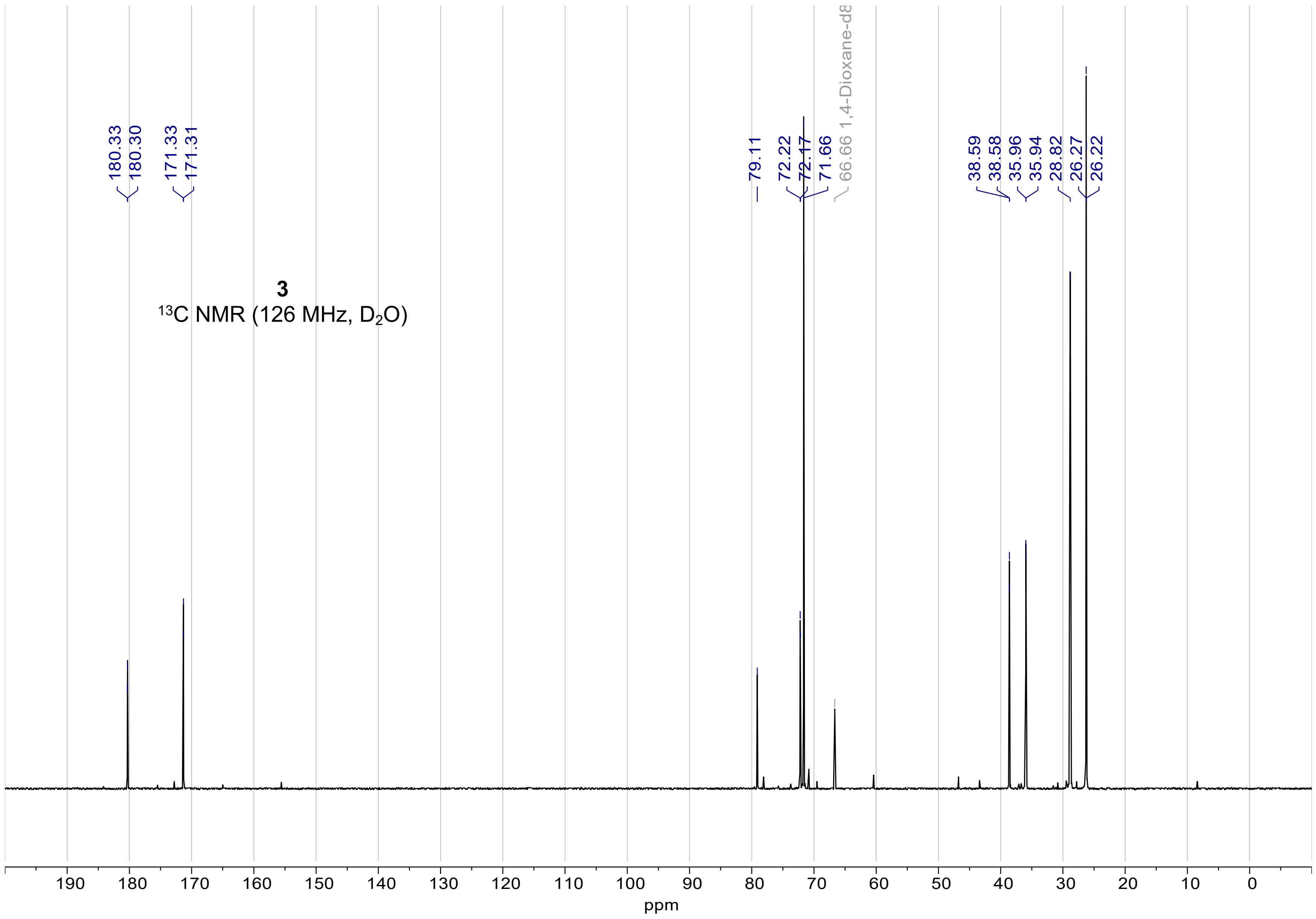

Figure S15. *^{13}C NMR spectrum of 4-amino-1-oxo-1-(prop-2-yn-1-ylamino)butan-2-yl pivalate hydrochloride **3**.*

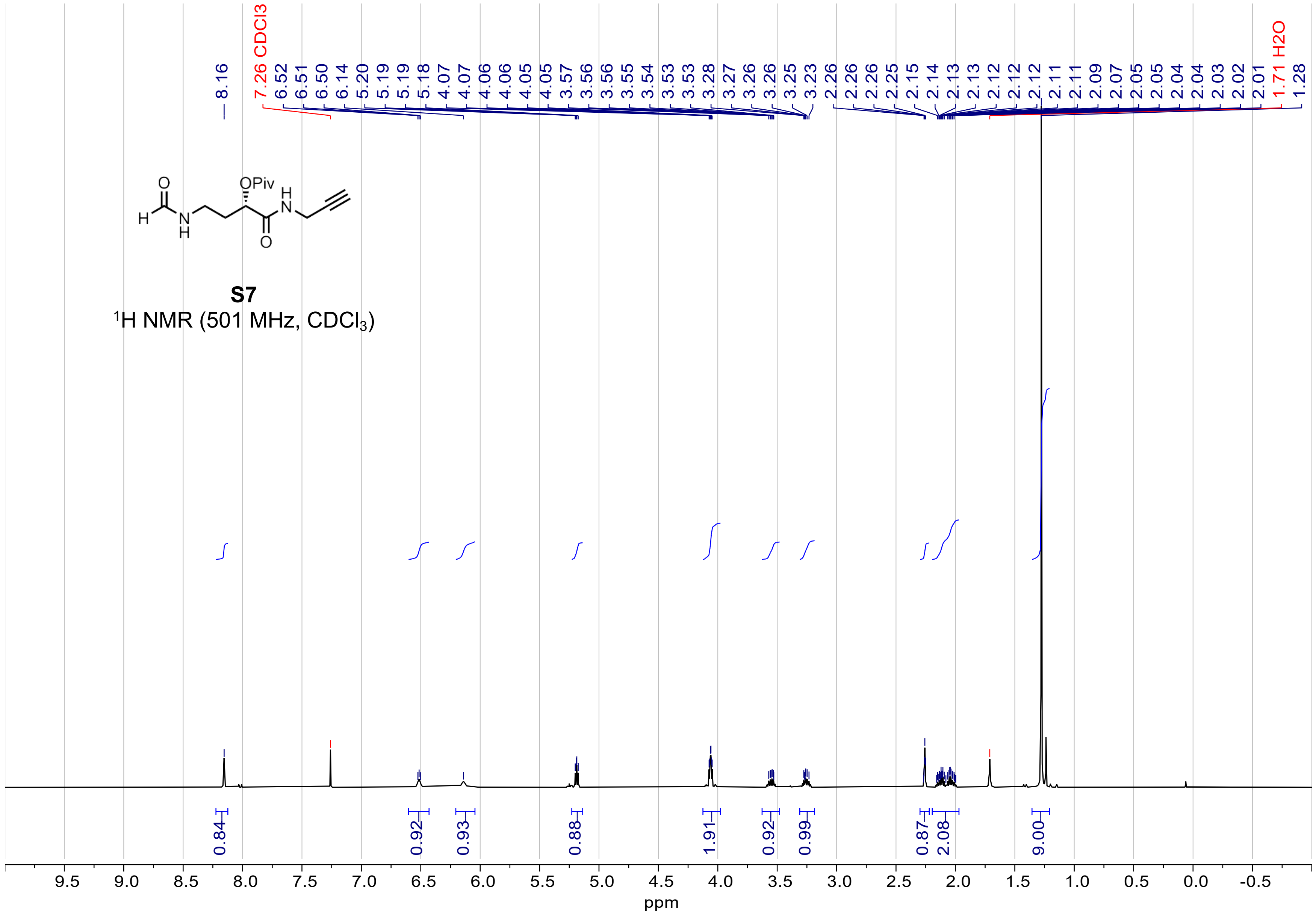

Figure S16. *^{1}H NMR spectrum of 4-formamido-1-oxo-1-(prop-2-yn-1-ylamino)butan-2-yl pivalate **S7**.*

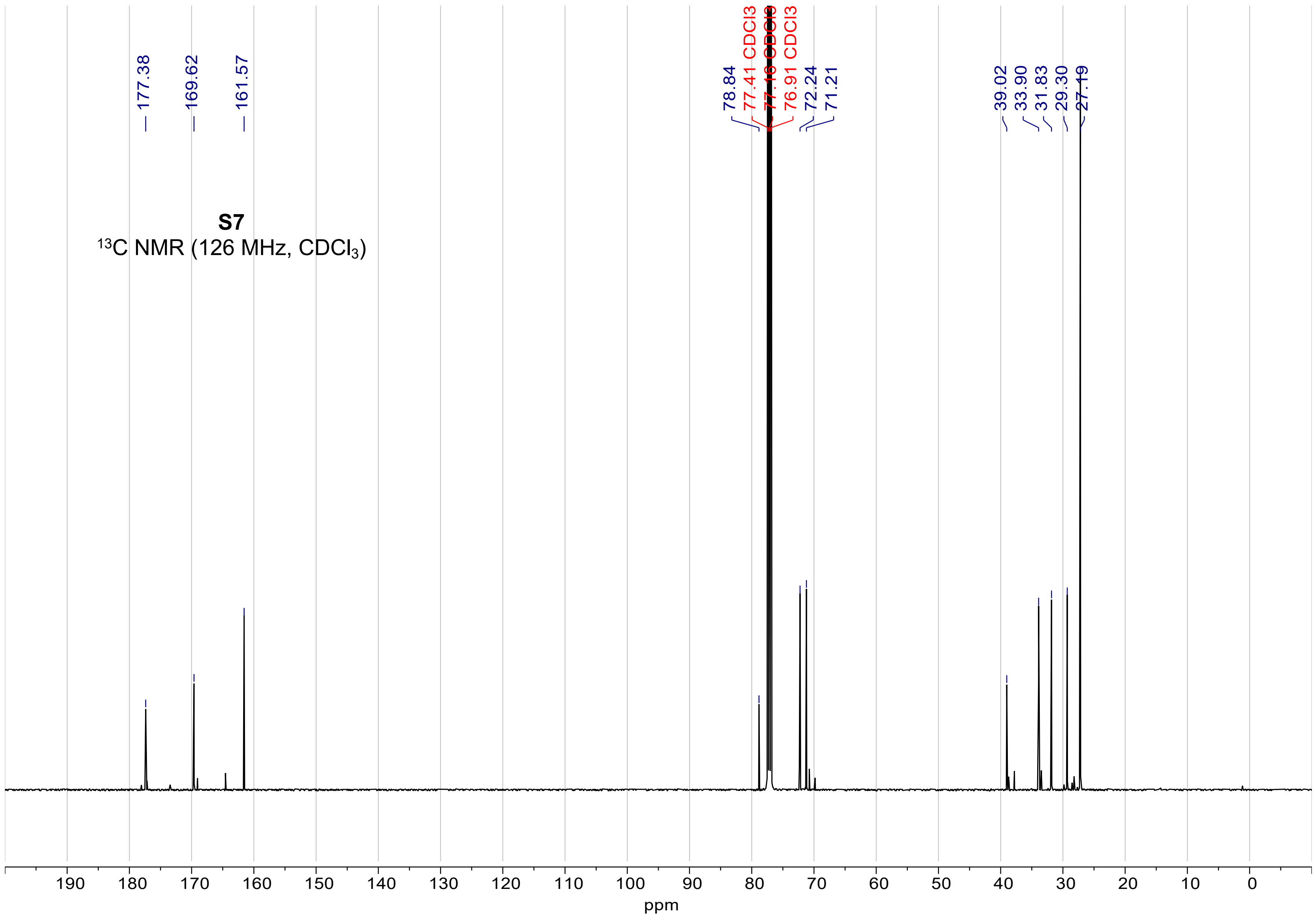

Figure S17. *^{13}C NMR spectrum of 4-formamido-1-oxo-1-(prop-2-yn-1-ylamino)butan-2-yl pivalate* ***S7****.*

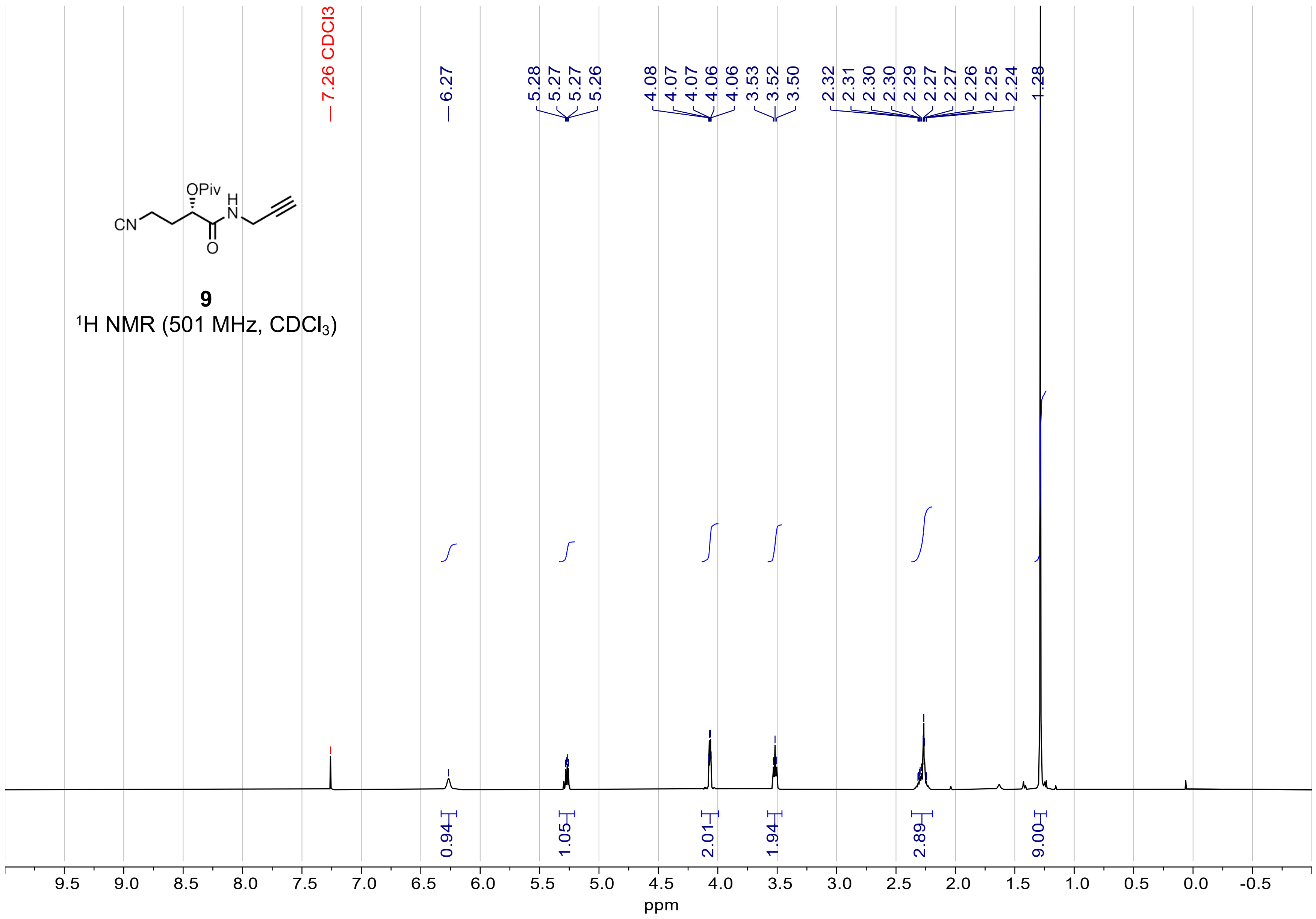

Figure S18. *^{1}H NMR spectrum of 4-isocyano-1-oxo-1-(prop-2-yn-1-ylamino)butan-2-yl pivalate **9**.*

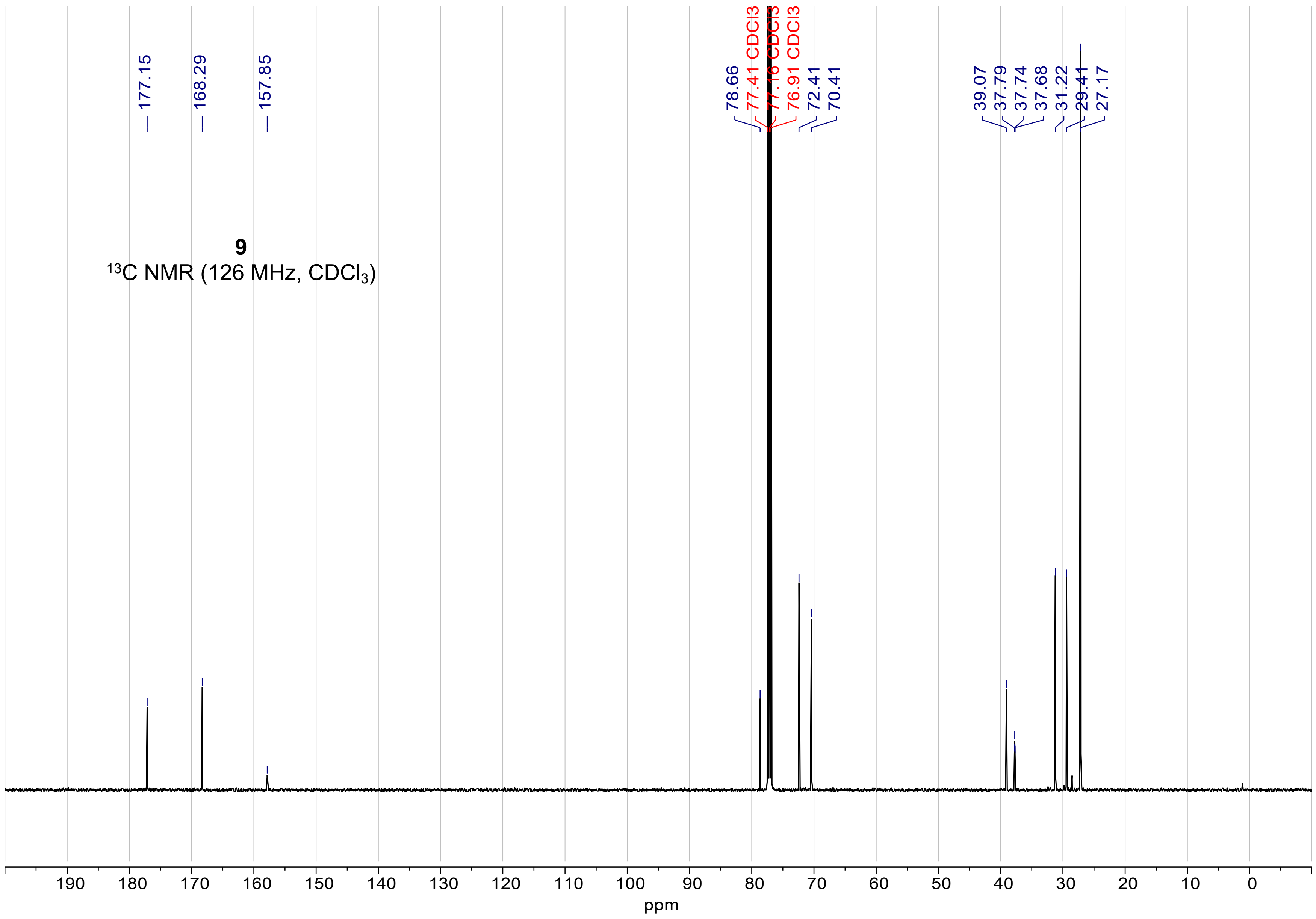

Figure S19. *^{13}C NMR spectrum of 4-isocyano-1-oxo-1-(prop-2-yn-1-ylamino)butan-2-yl pivalate **9**.*

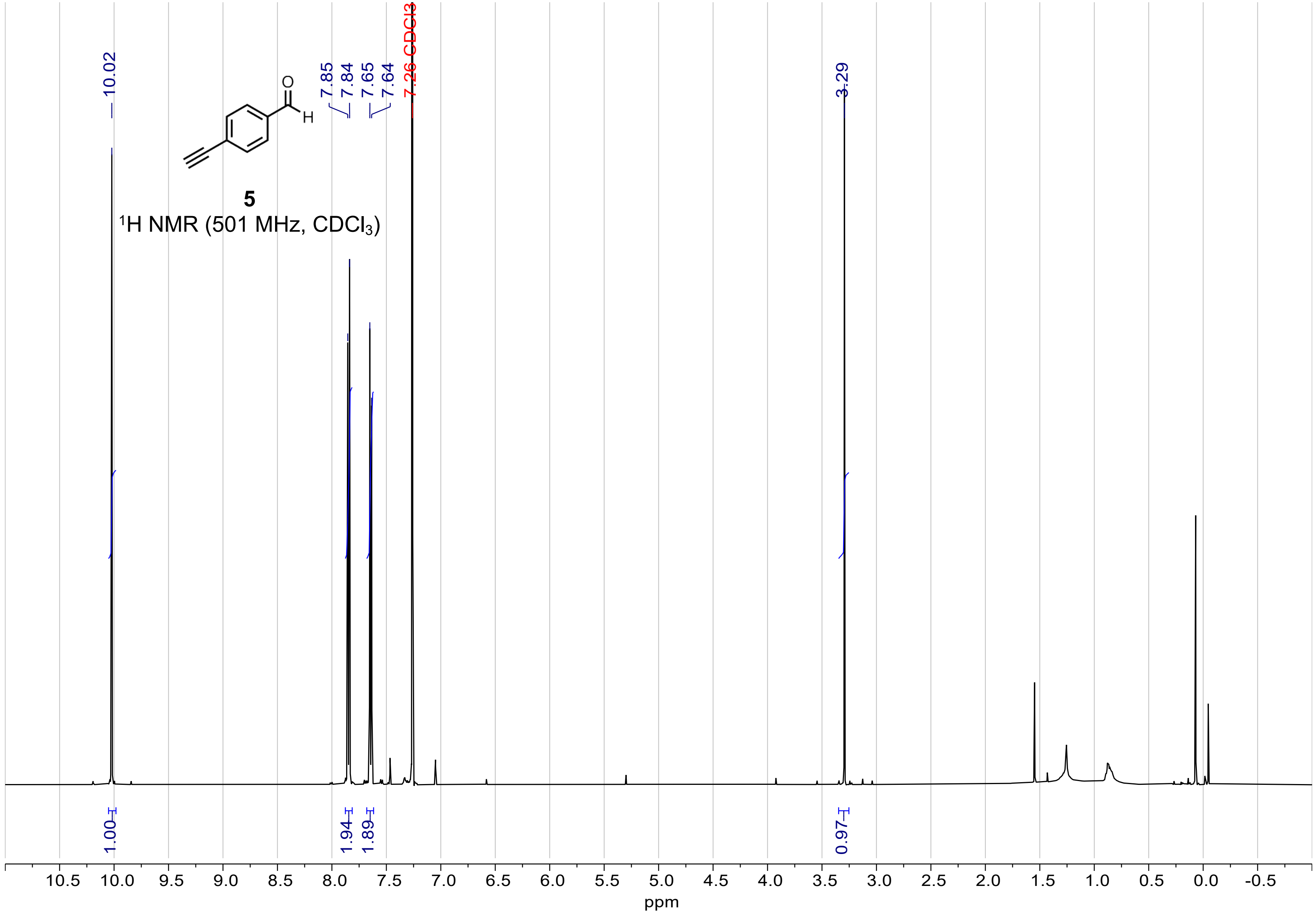

Figure S20. *^{1}H NMR spectrum of 4-ethynylbenzaldehyde **5**.*

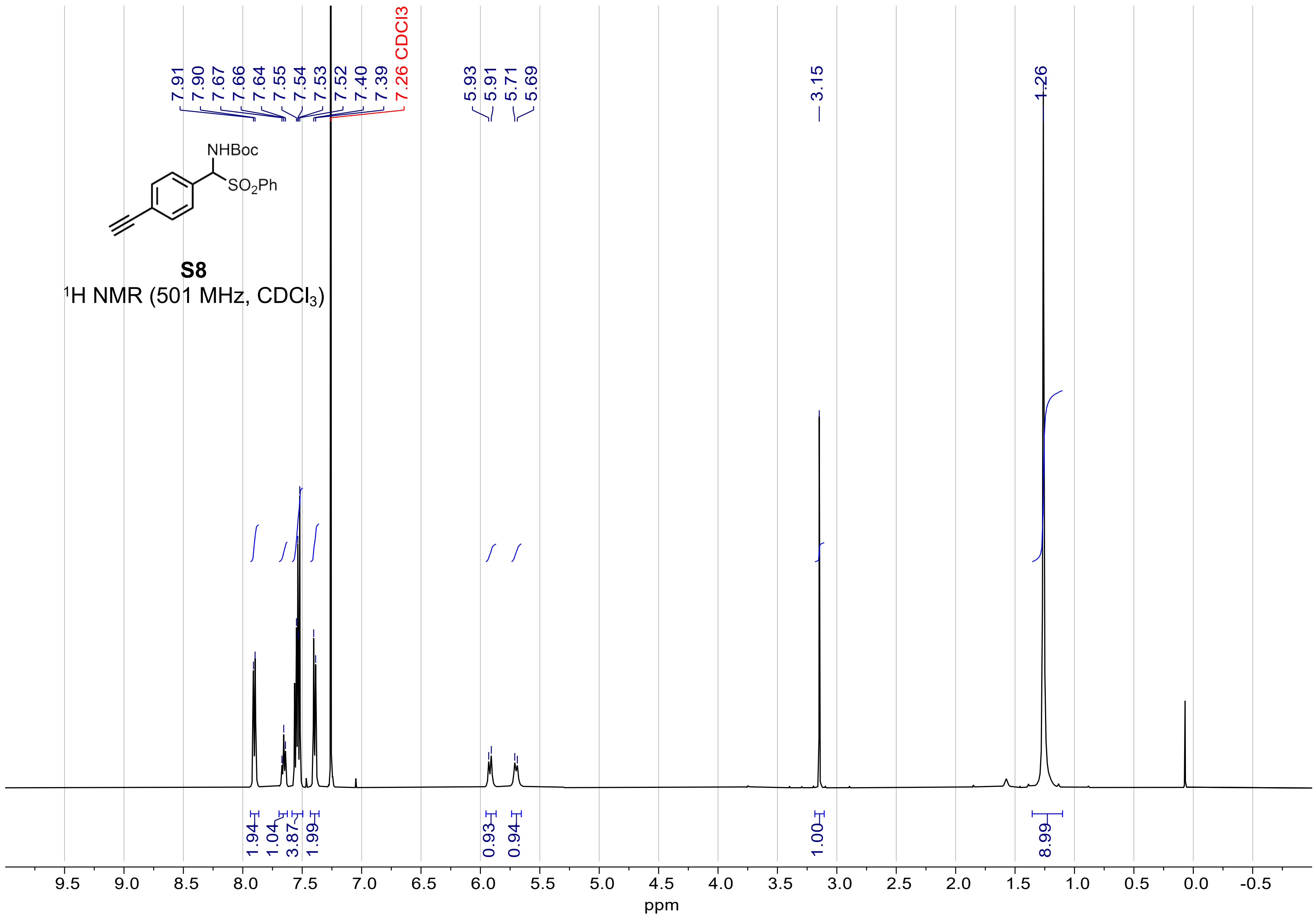

Figure S21. *^{1}H NMR spectrum of tert-butyl ((4-ethynylphenyl)(phenylsulfonyl)methyl)carbamate **S8**.*

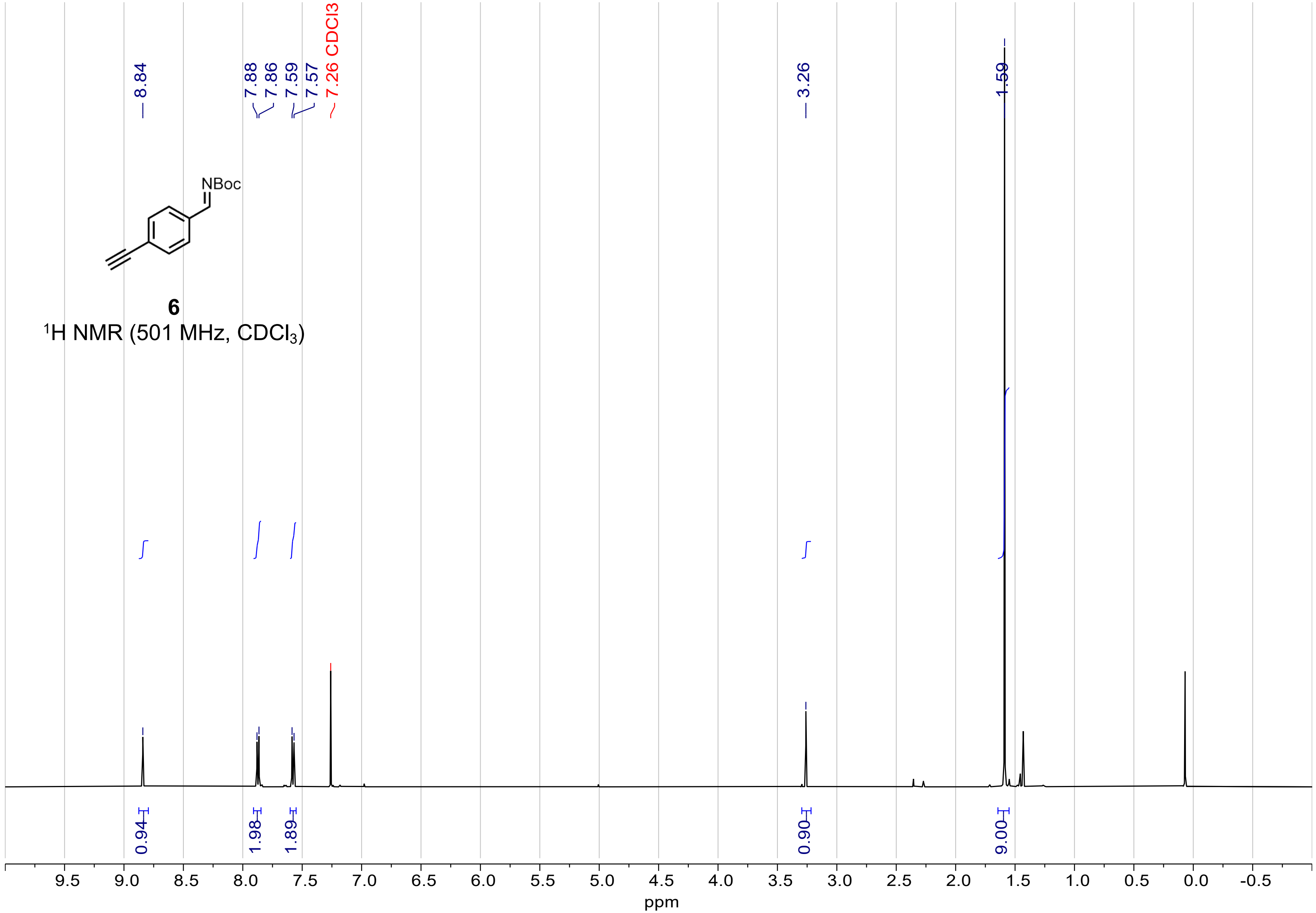

Figure S22. *^{1}H NMR spectrum of tert-butyl (4-ethynylbenzylidene)carbamate* ***6****.*

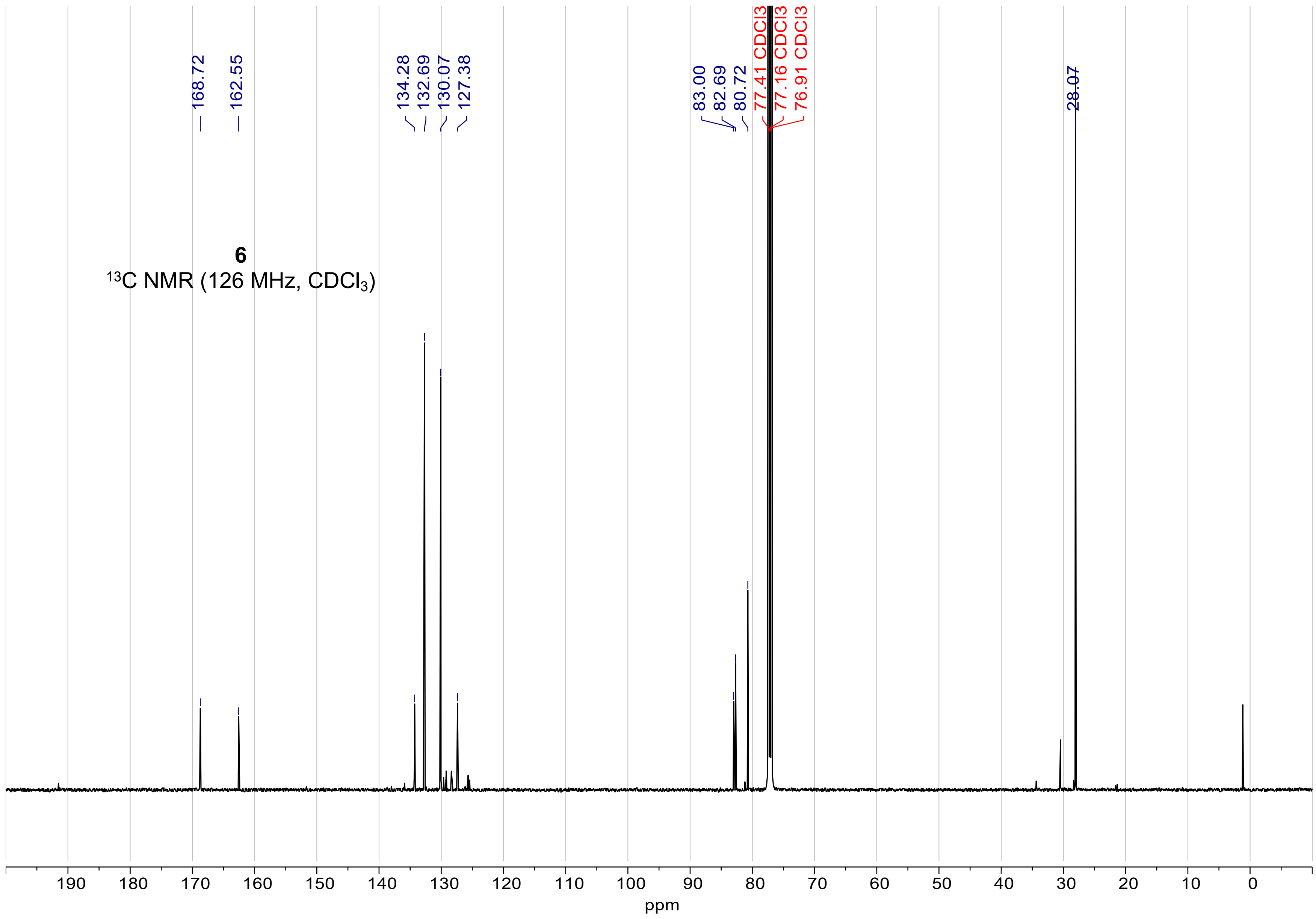

Figure S23. *^{13}C NMR spectrum of tert-butyl (4-ethynylbenzylidene)carbamate **6**.*

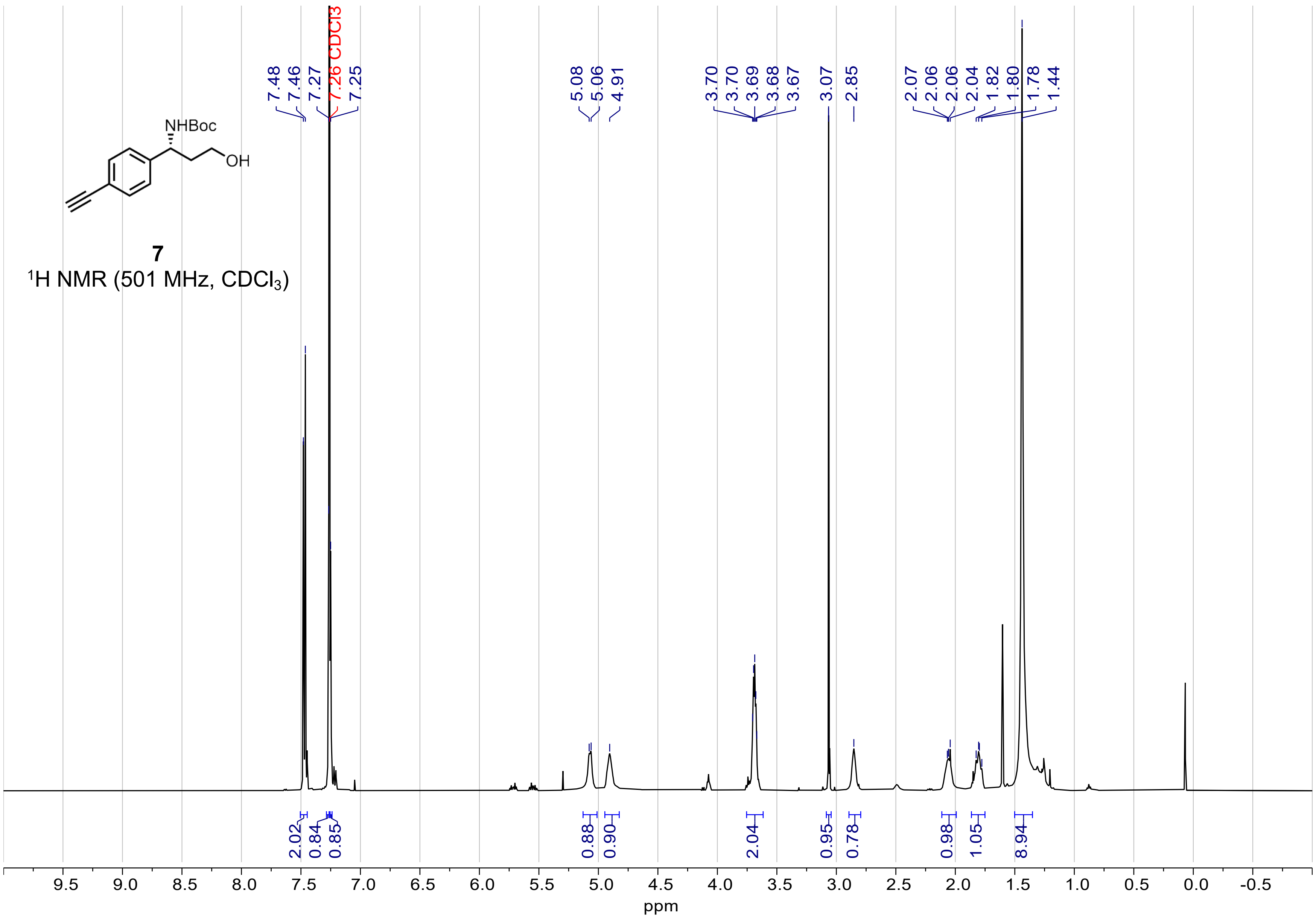

Figure S24. *^{1}H NMR spectrum of tert-butyl (R)-(1-(4-ethynylphenyl)-3-hydroxypropyl)carbamate **7**.*

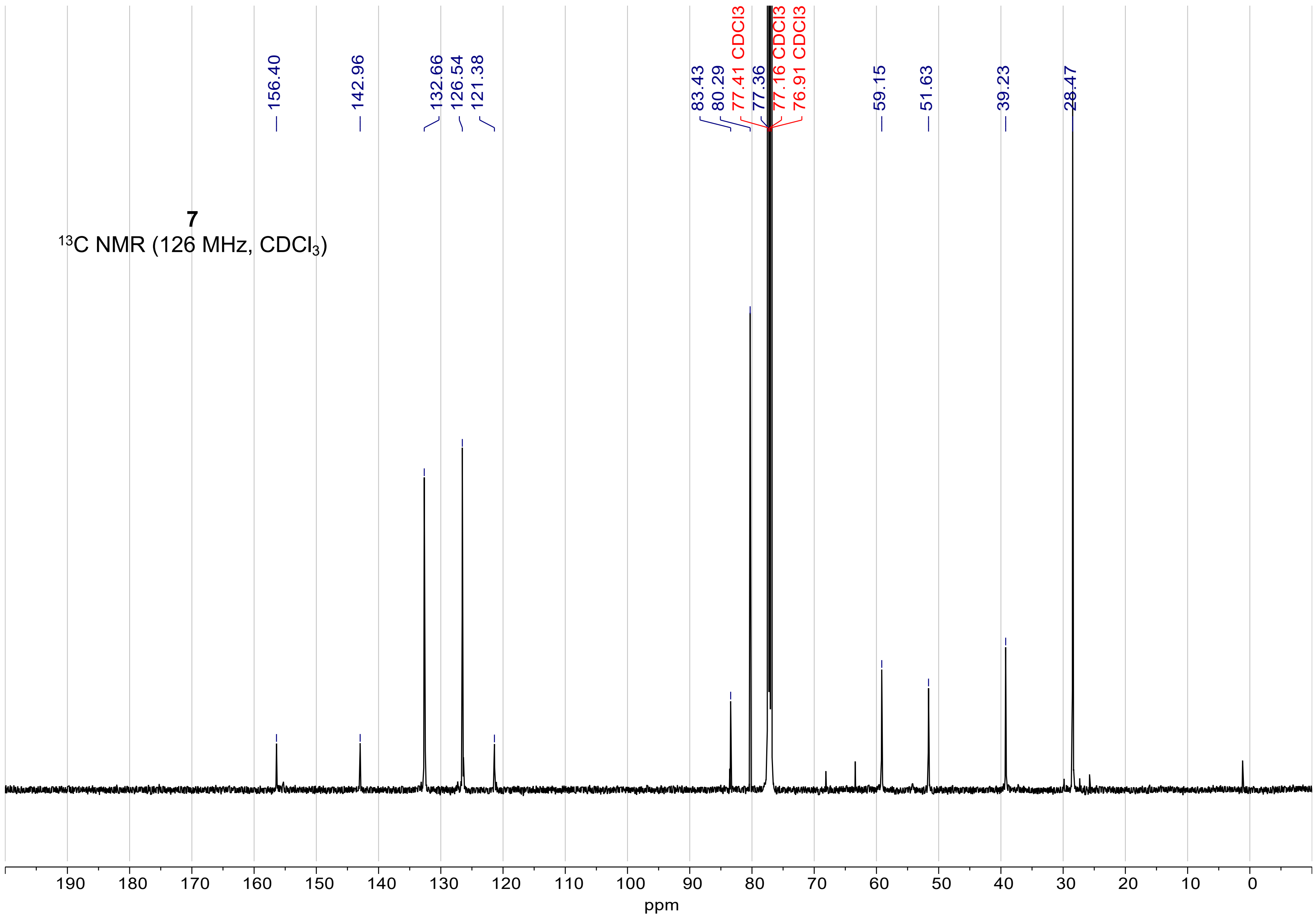

Figure S25. *^{13}C NMR spectrum of tert-butyl (R)-(1-(4-ethynylphenyl)-3-hydroxypropyl)carbamate* ***7****.*

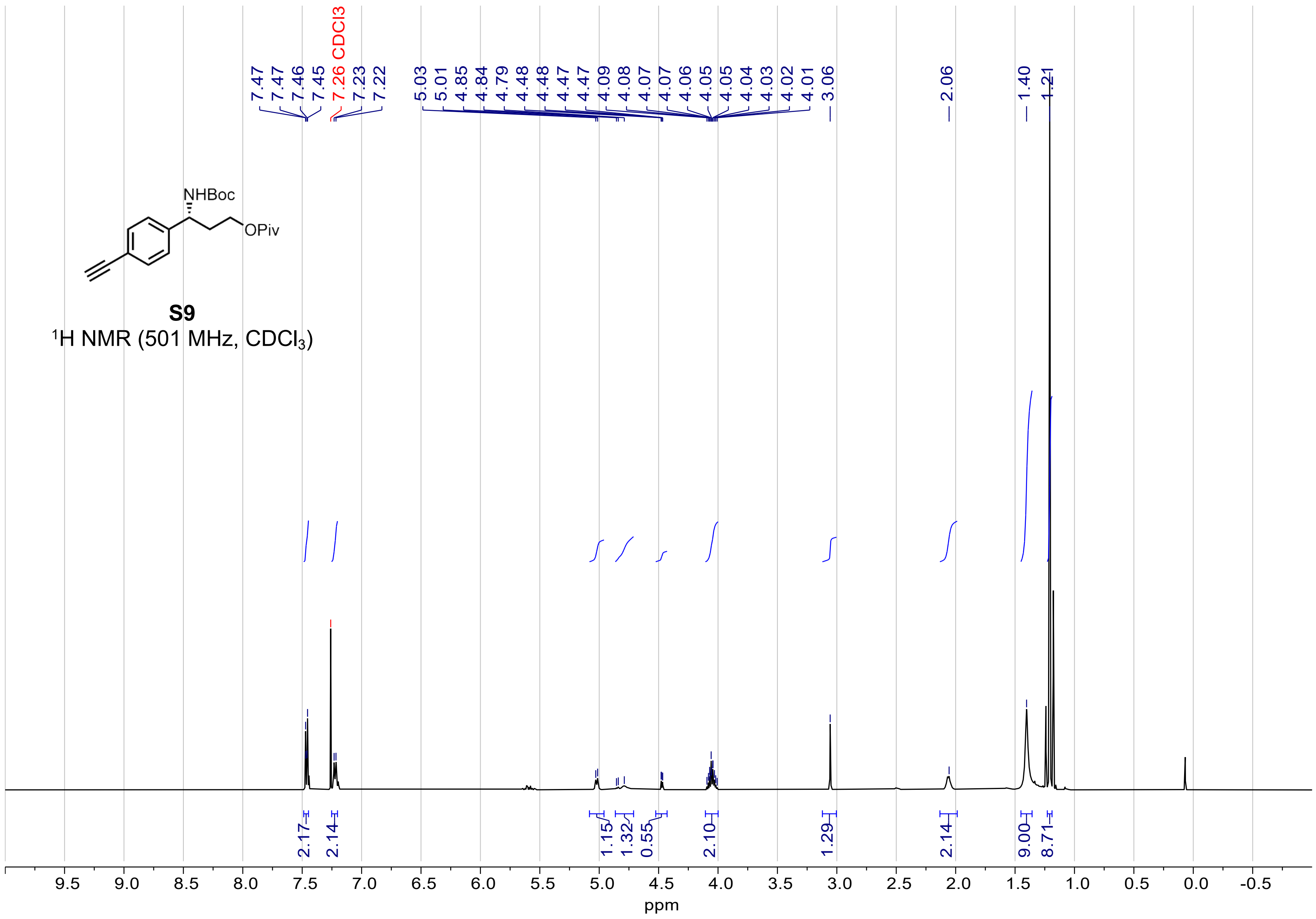

Figure S26. *^{1}H NMR spectrum of tert-butyl (R)-3-((tert-Butoxycarbonyl)amino)-3-(4-ethynylphenyl)propyl pivalate **S9**.*

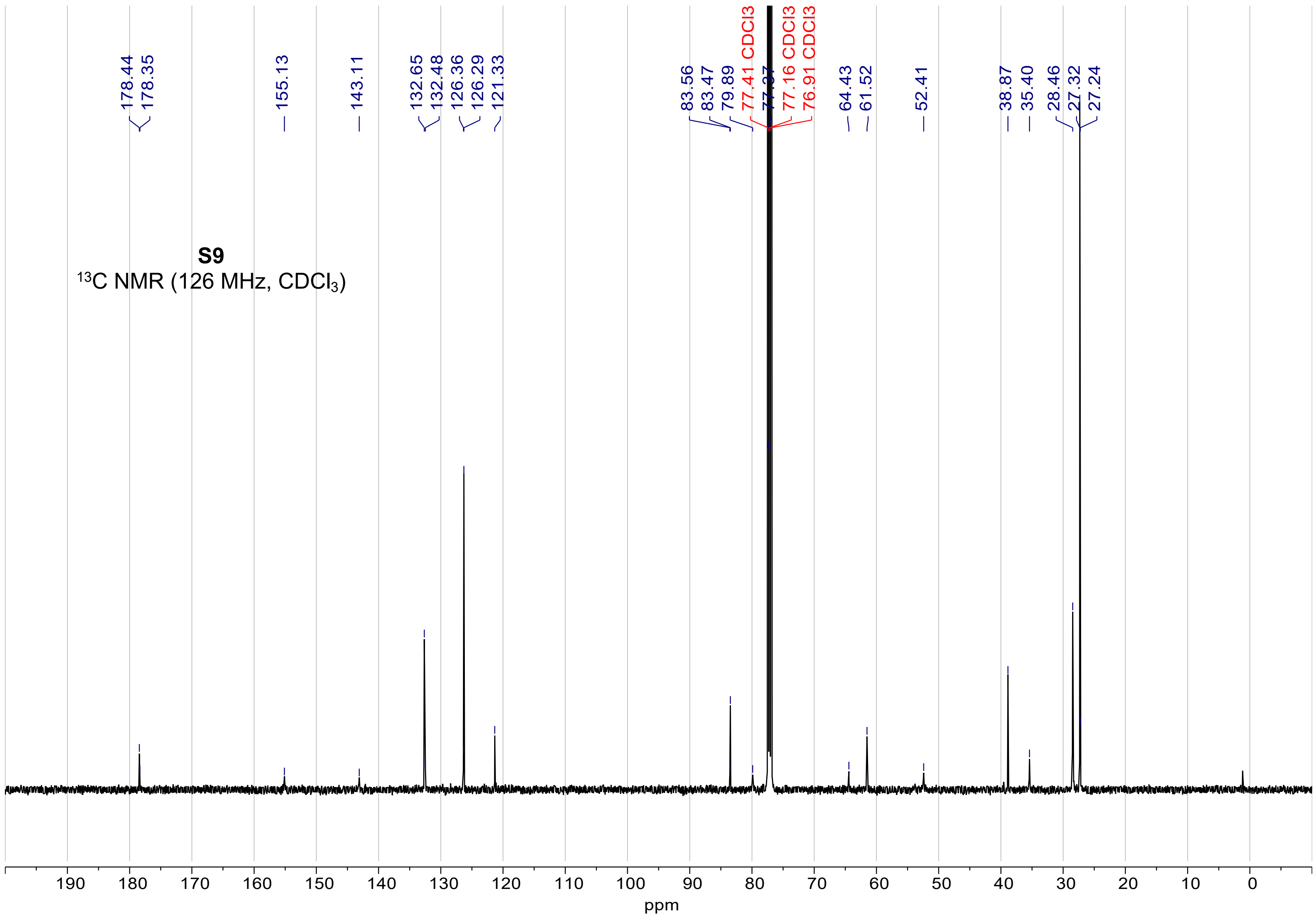

Figure S27. *^{13}C NMR spectrum of tert-butyl (R)-3-((tert-Butoxycarbonyl)amino)-3-(4-ethynylphenyl)propyl pivalate* ***S9****.*

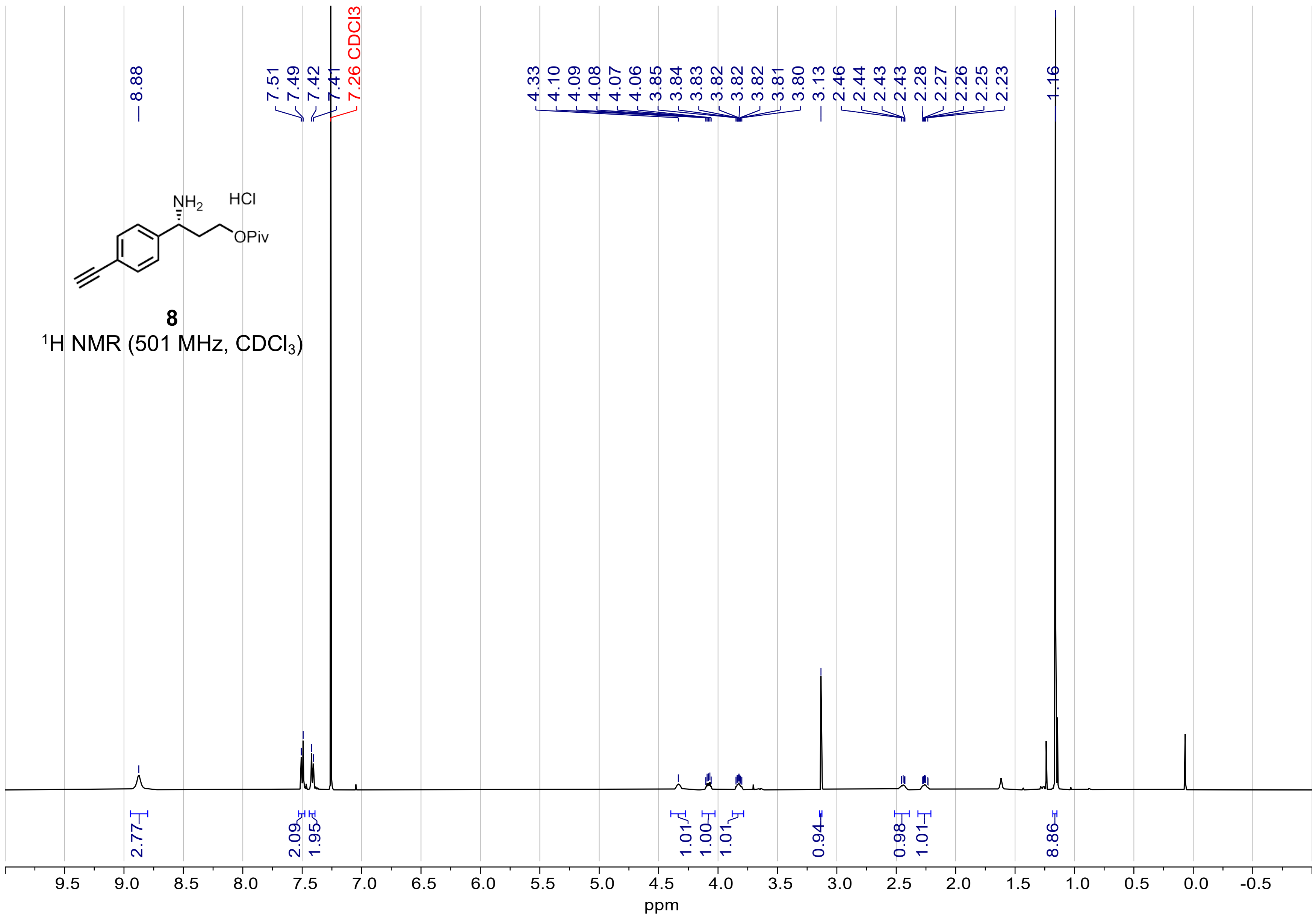

Figure S28. *^{1}H NMR spectrum of (R)-3-amino-3-(4-ethynylphenyl)propyl pivalate hydrogen chloride **8**.*

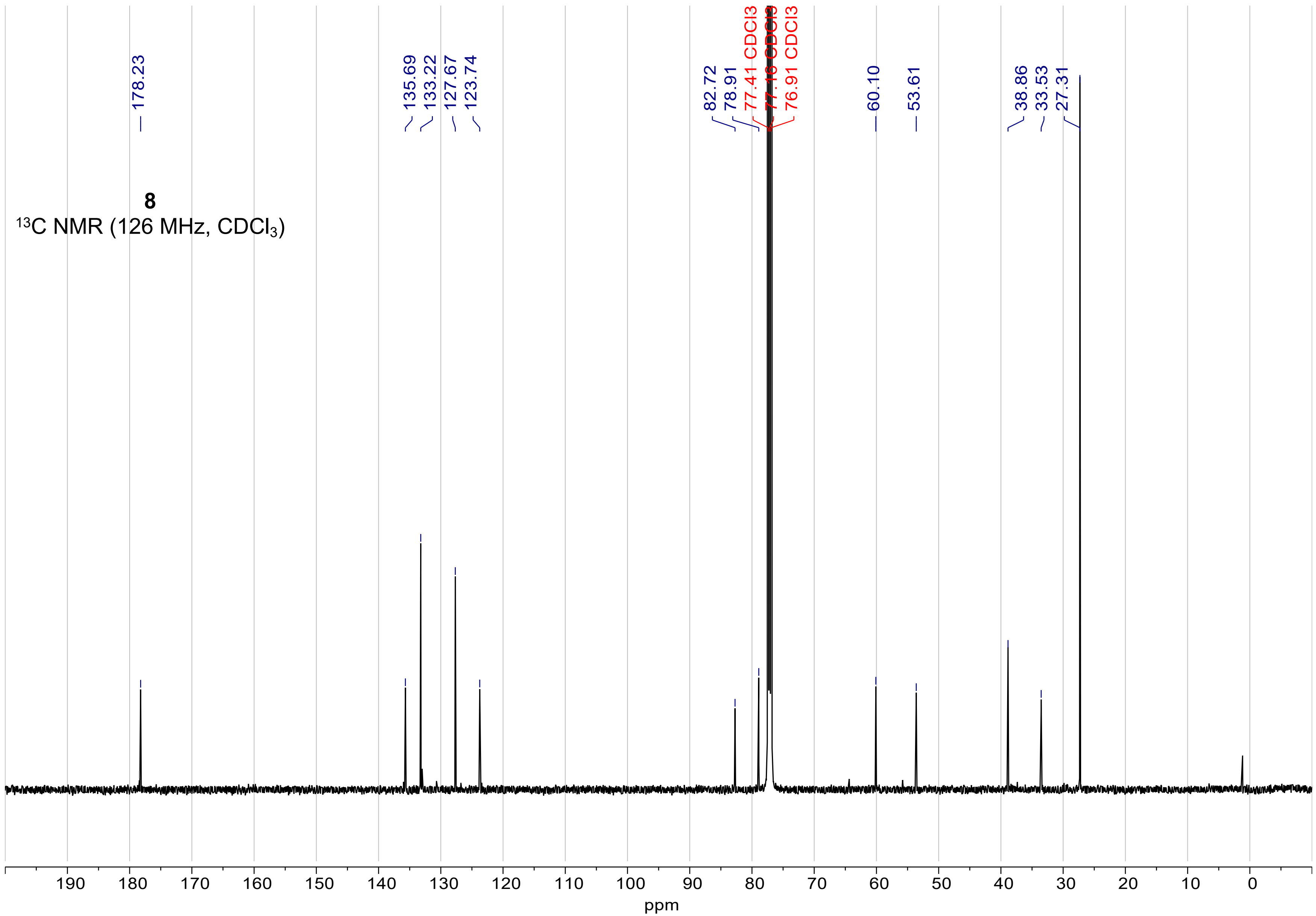

Figure S29. *^{13}C NMR spectrum of (R)-3-amino-3-(4-ethynylphenyl)propyl pivalate hydrogen chloride* ***8****.*

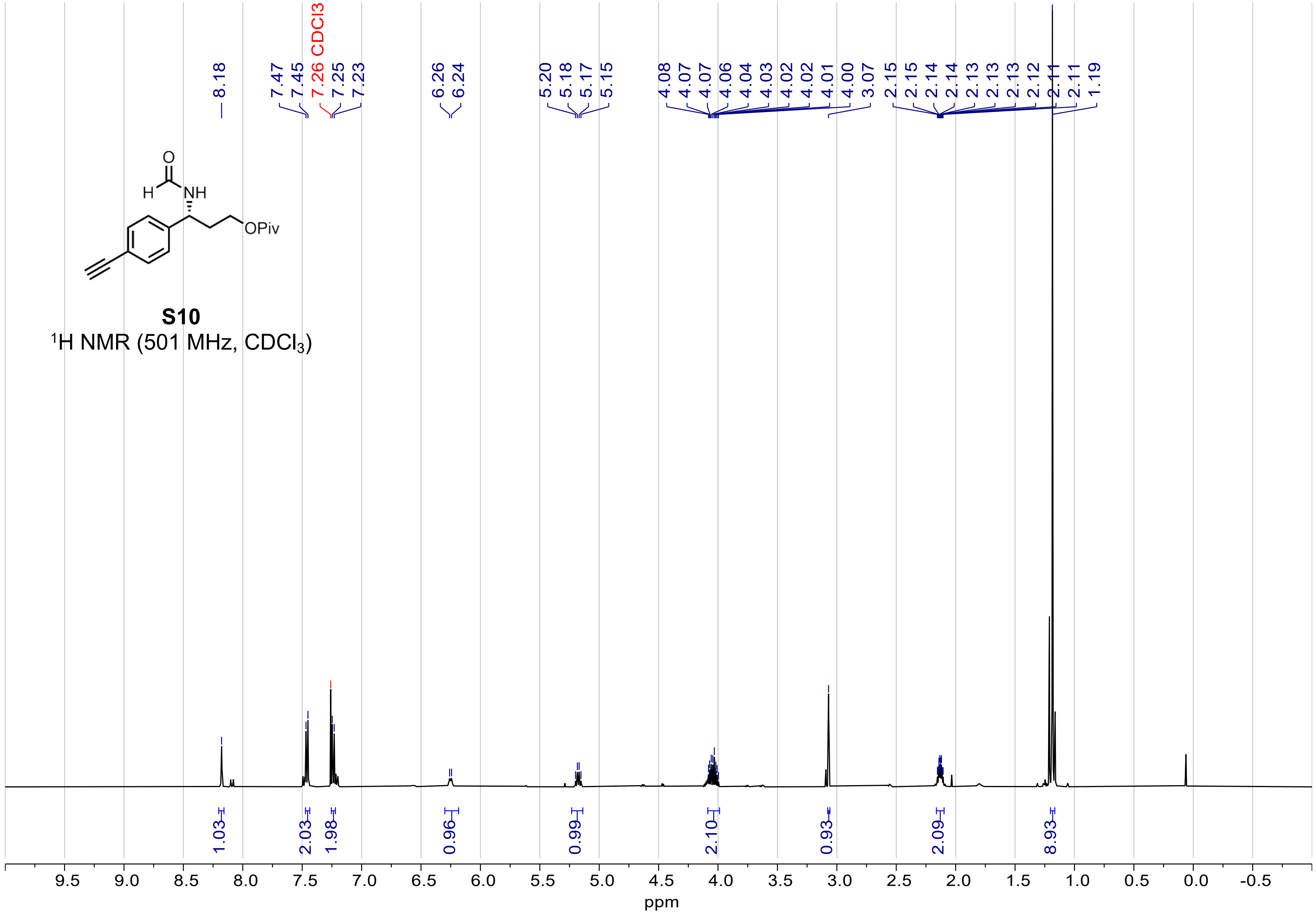

Figure S30. *^{1}H NMR spectrum of (R)-3-(4-ethynylphenyl)-3-formamidopropyl pivalate* ***S10***.

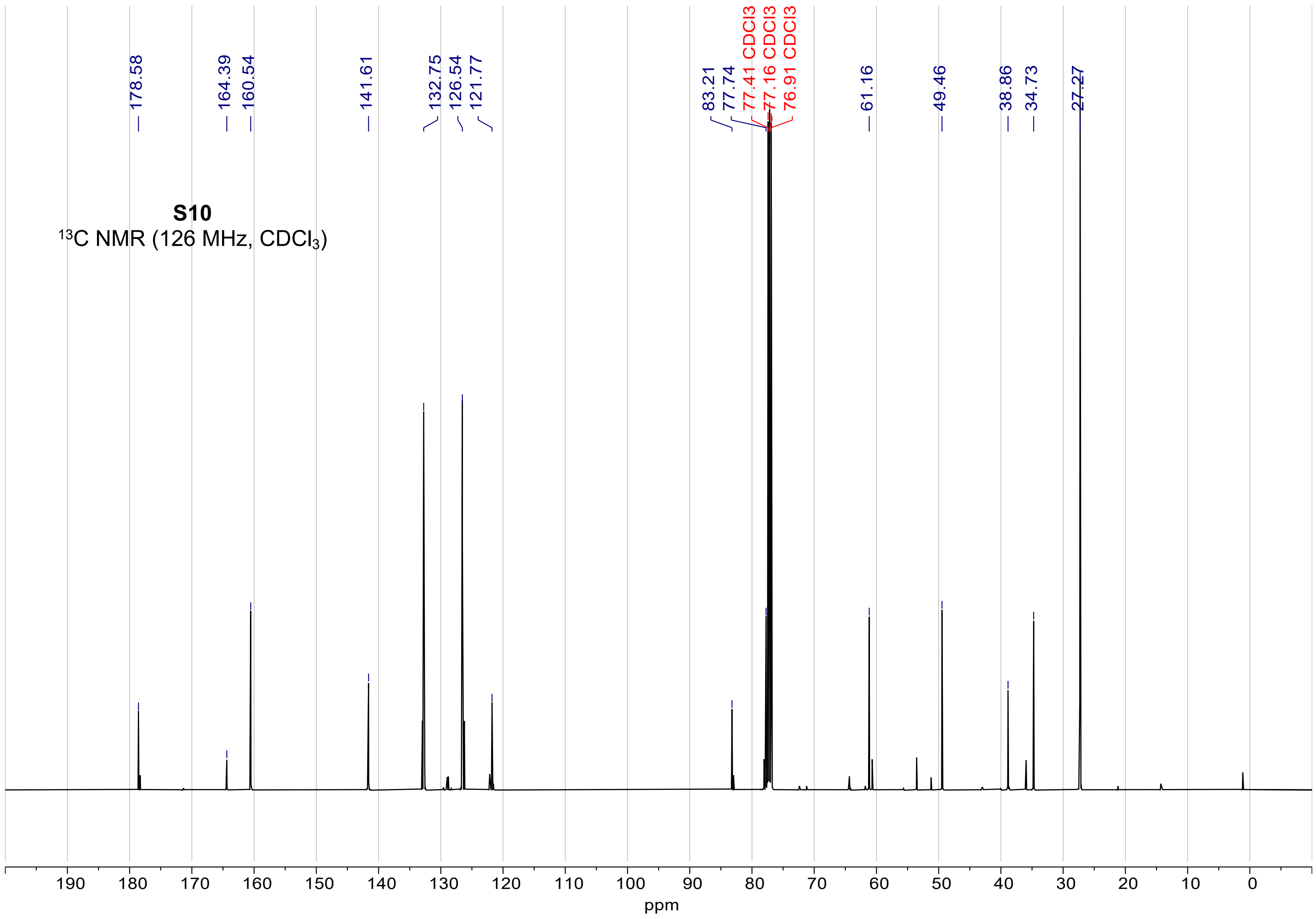

Figure S31. *^{13}C NMR spectrum of (R)-3-(4-ethynylphenyl)-3-formamidopropyl pivalate **S10**.*

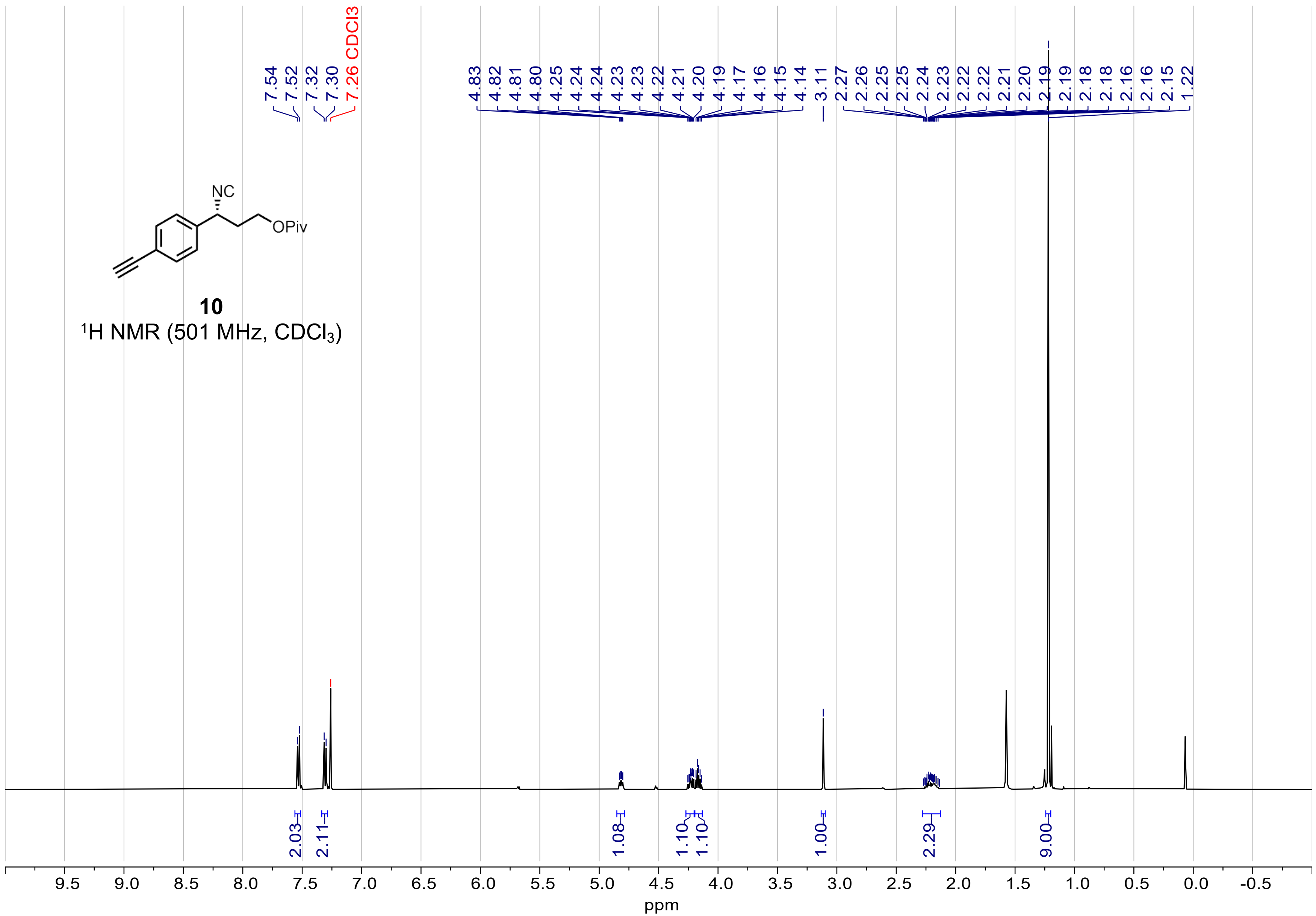

Figure S32. *^{1}H NMR spectrum of (R)-3-(4-ethynylphenyl)-3-isocyanopropyl pivalate* ***10***.

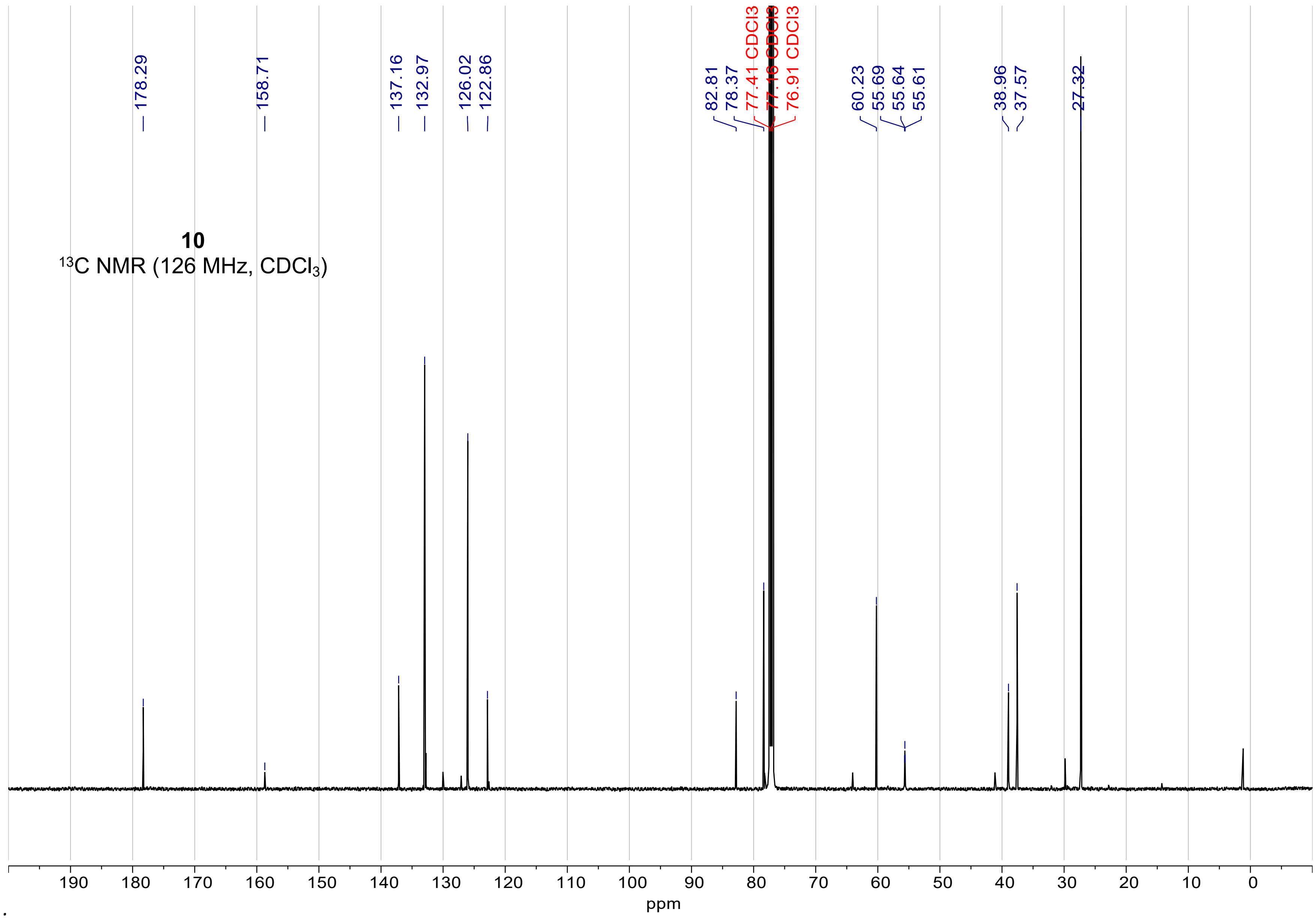

Figure S33. *^{13}C NMR spectrum of (R)-3-(4-ethynylphenyl)-3-isocyanopropyl pivalate **10**.*

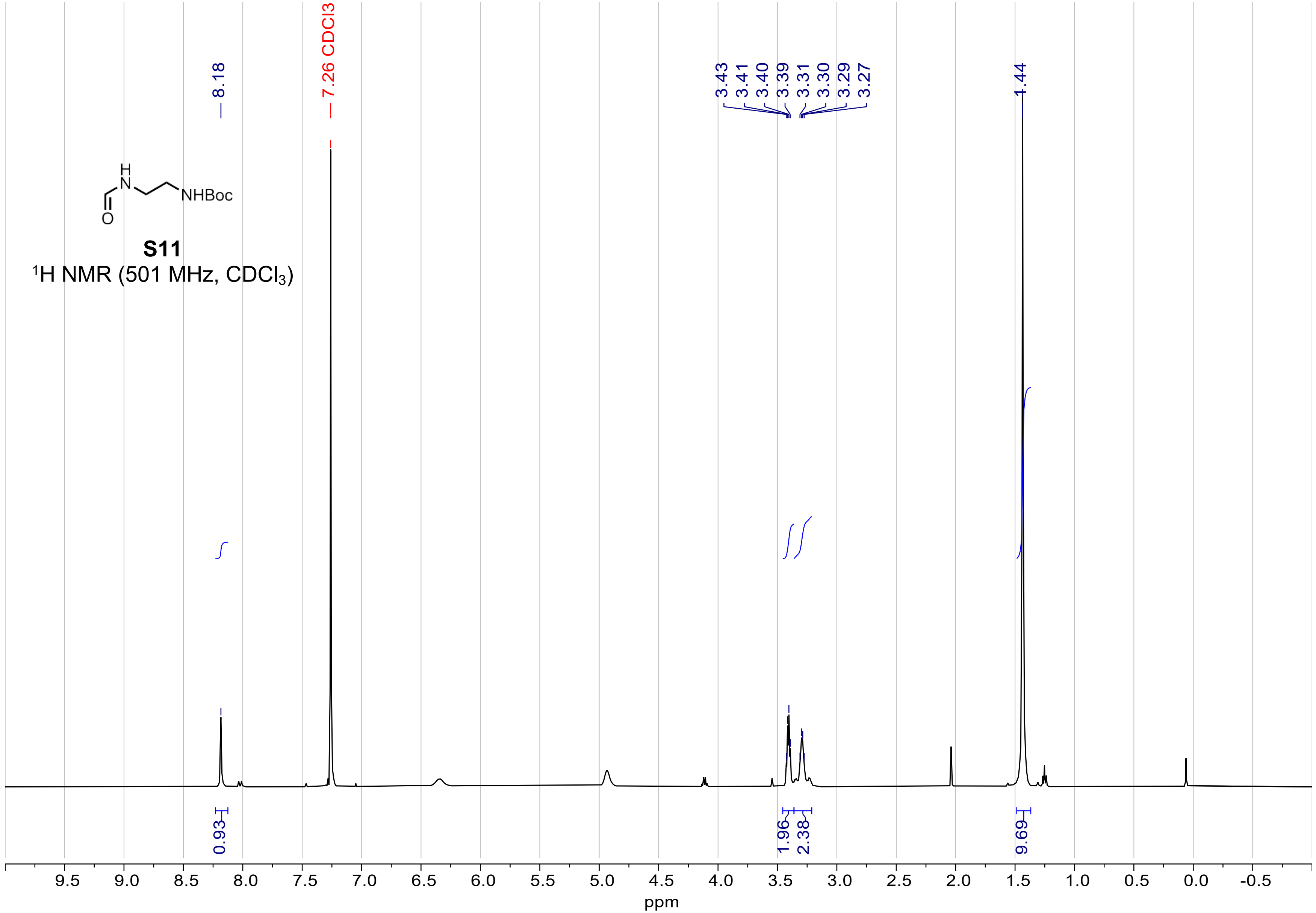

Figure S34. *^{1}H NMR spectrum of tert-butyl (2-formamidoethyl)carbamate **S11**.*

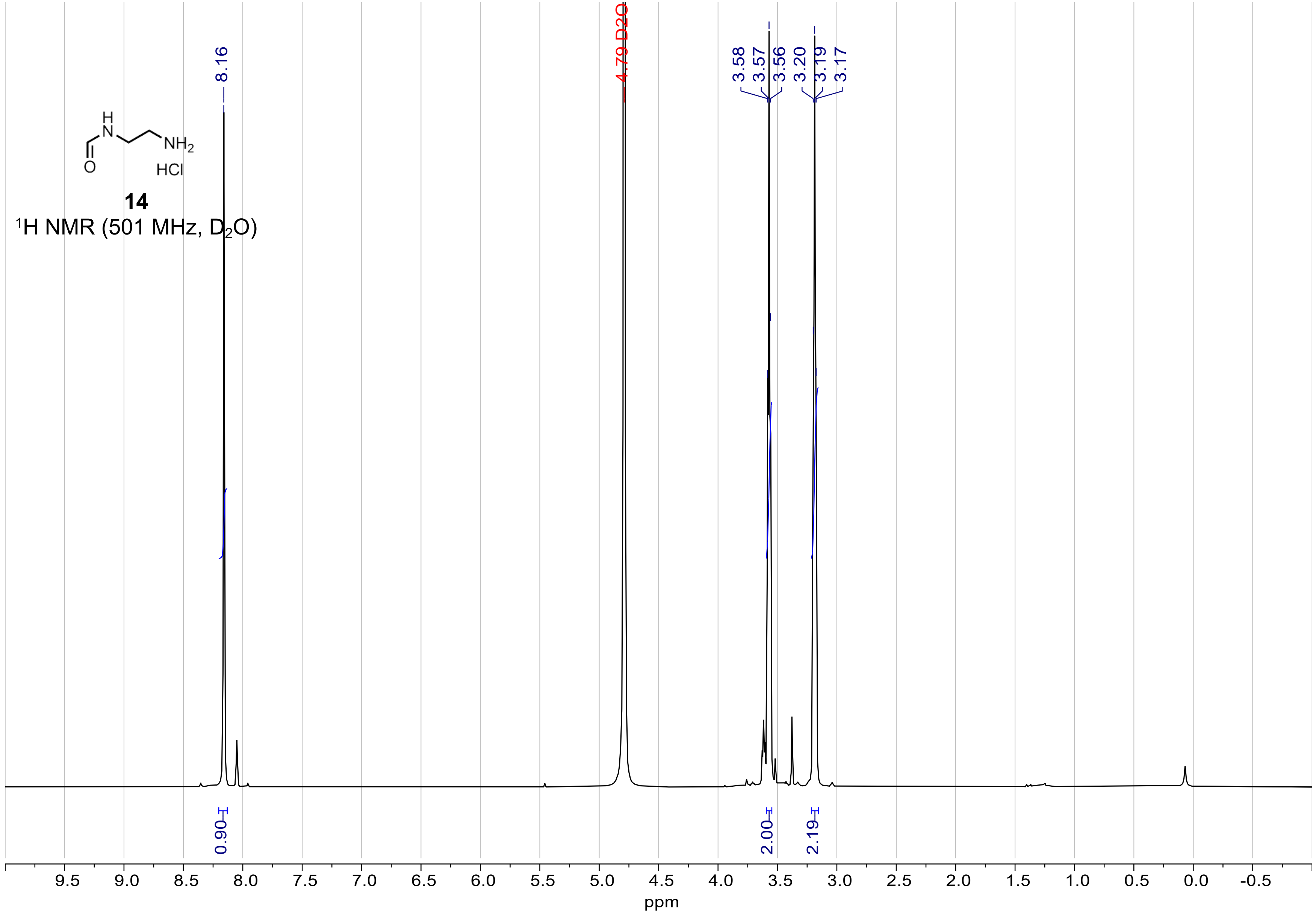

Figure S35. *^{1}H NMR spectrum of N-(2-aminoethyl)formamide hydrochloride* ***14****.*

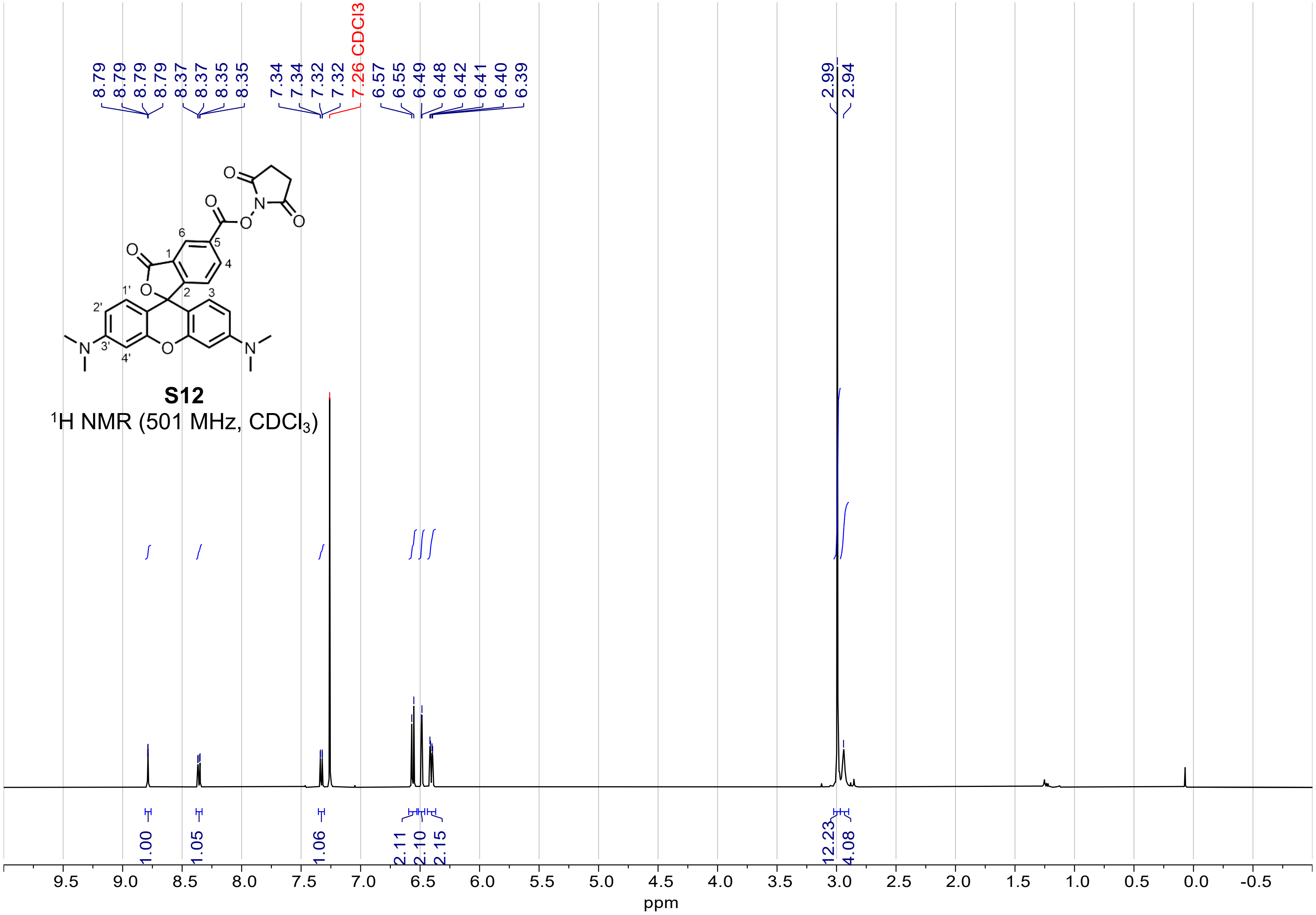

Figure S36. *^{1}H NMR spectrum of 2,5-dioxopyrrolidin-1-yl 3',6'-bis(dimethylamino)-3-oxo-3H-spiro[isobenzofuran-1,9'-xanthene]-5-carboxylate* ***S12****.*

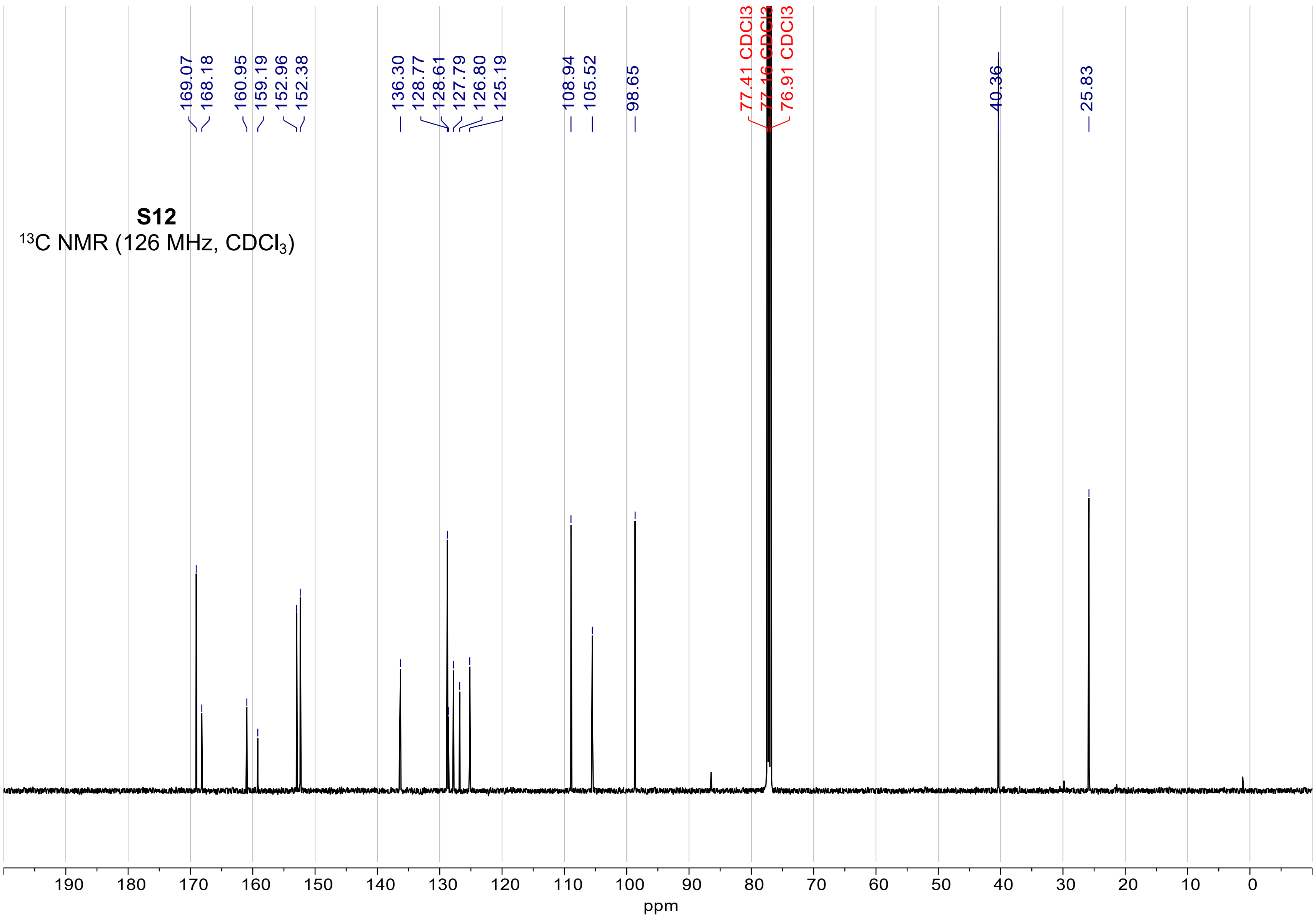

Figure S37. *^{13}C NMR spectrum of 2,5-dioxopyrrolidin-1-yl 3',6'-bis(dimethylamino)-3-oxo-3H-spiro[isobenzofuran-1,9'-xanthene]-5-carboxylate* ***S12****.*

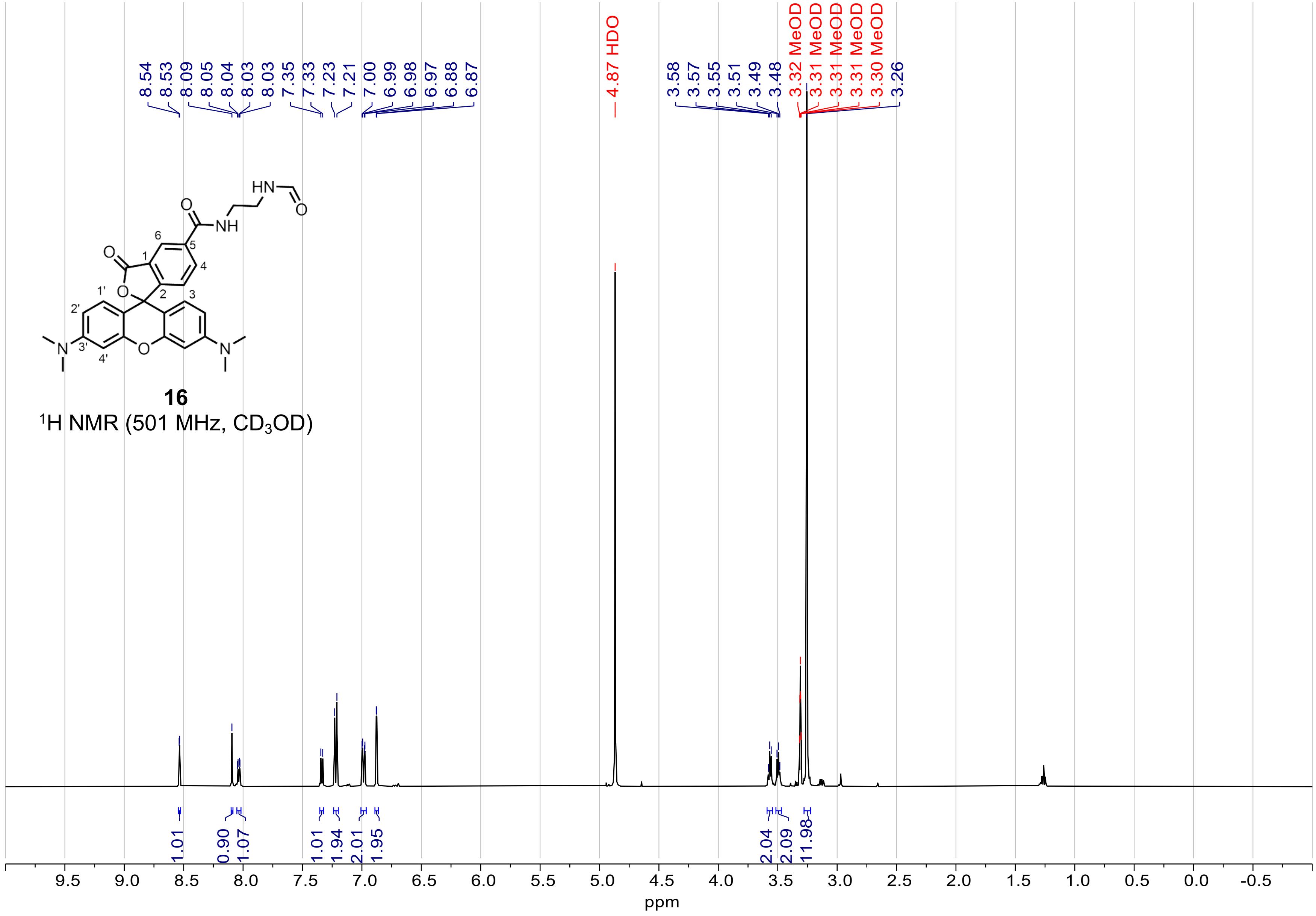

16

^{1}H NMR (501 MHz, CD_3OD)

Figure S38. *^{1}H NMR spectrum of 3',6'-bis(dimethylamino)-N-(2-formamidoethyl)-3-oxo-3H-spiro[isobenzofuran-1,9'-xanthene]-5-carboxamide* ***16***.

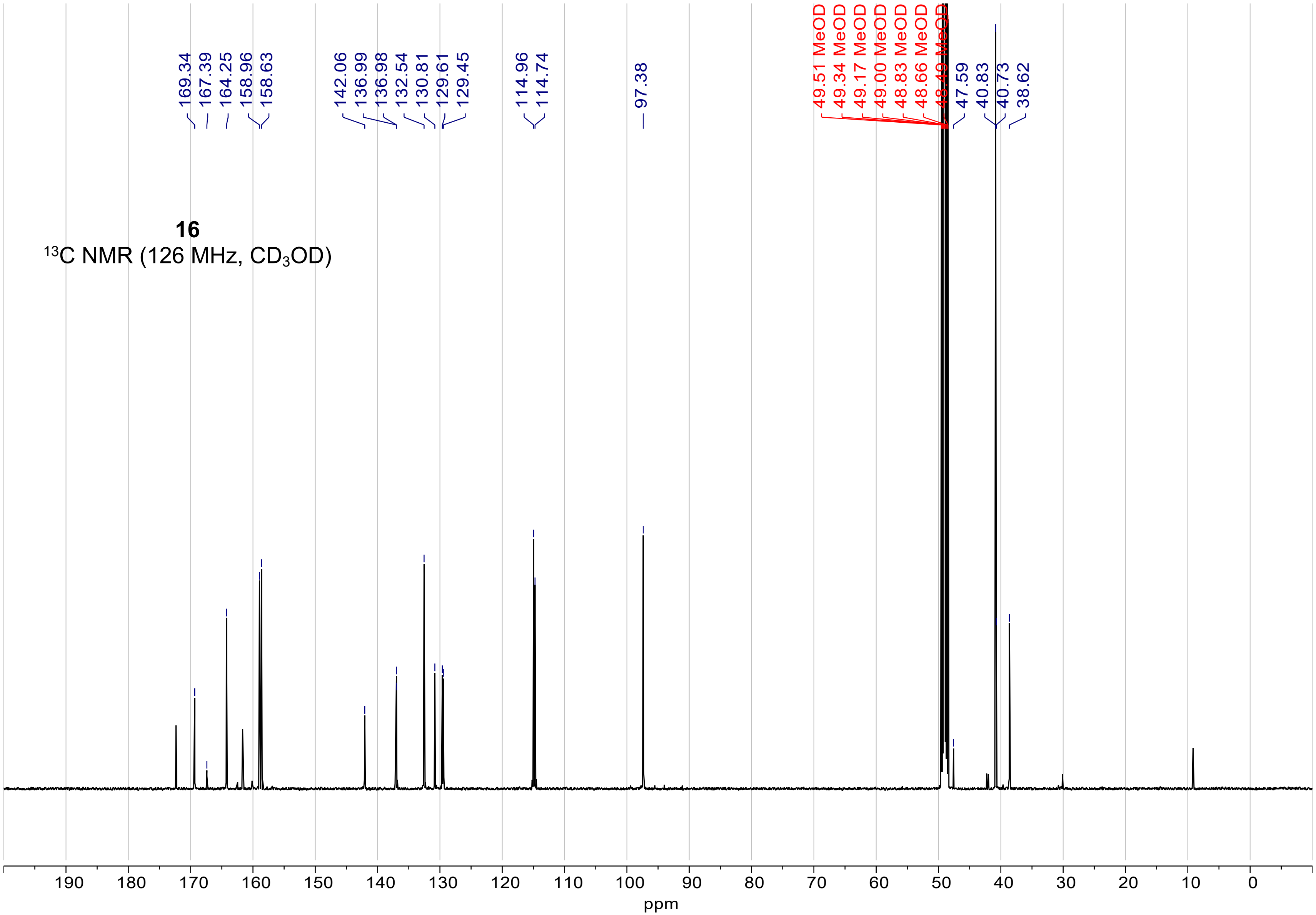

Figure S39. *^{13}C NMR spectrum of 3',6'-bis(dimethylamino)-N-(2-formamidoethyl)-3-oxo-3H-spiro[isobenzofuran-1,9'-xanthene]-5-carboxamide* ***16***.

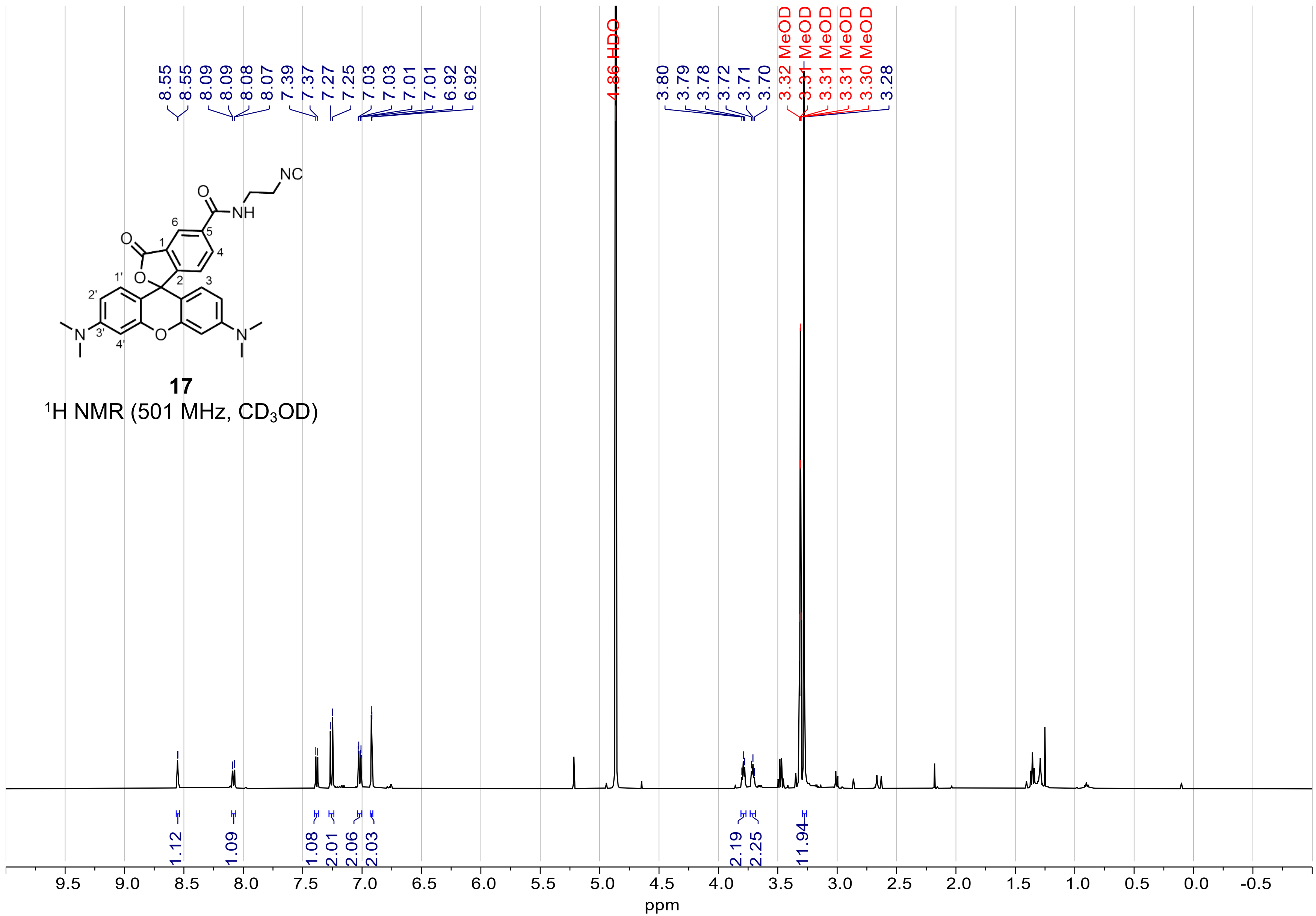

Figure S40. *^{1}H NMR spectrum of 3',6'-bis(dimethylamino)-N-(2-isocyanoethyl)-3-oxo-3H-spiro[isobenzofuran-1,9'-xanthene]-5-carboxamide* ***17***.

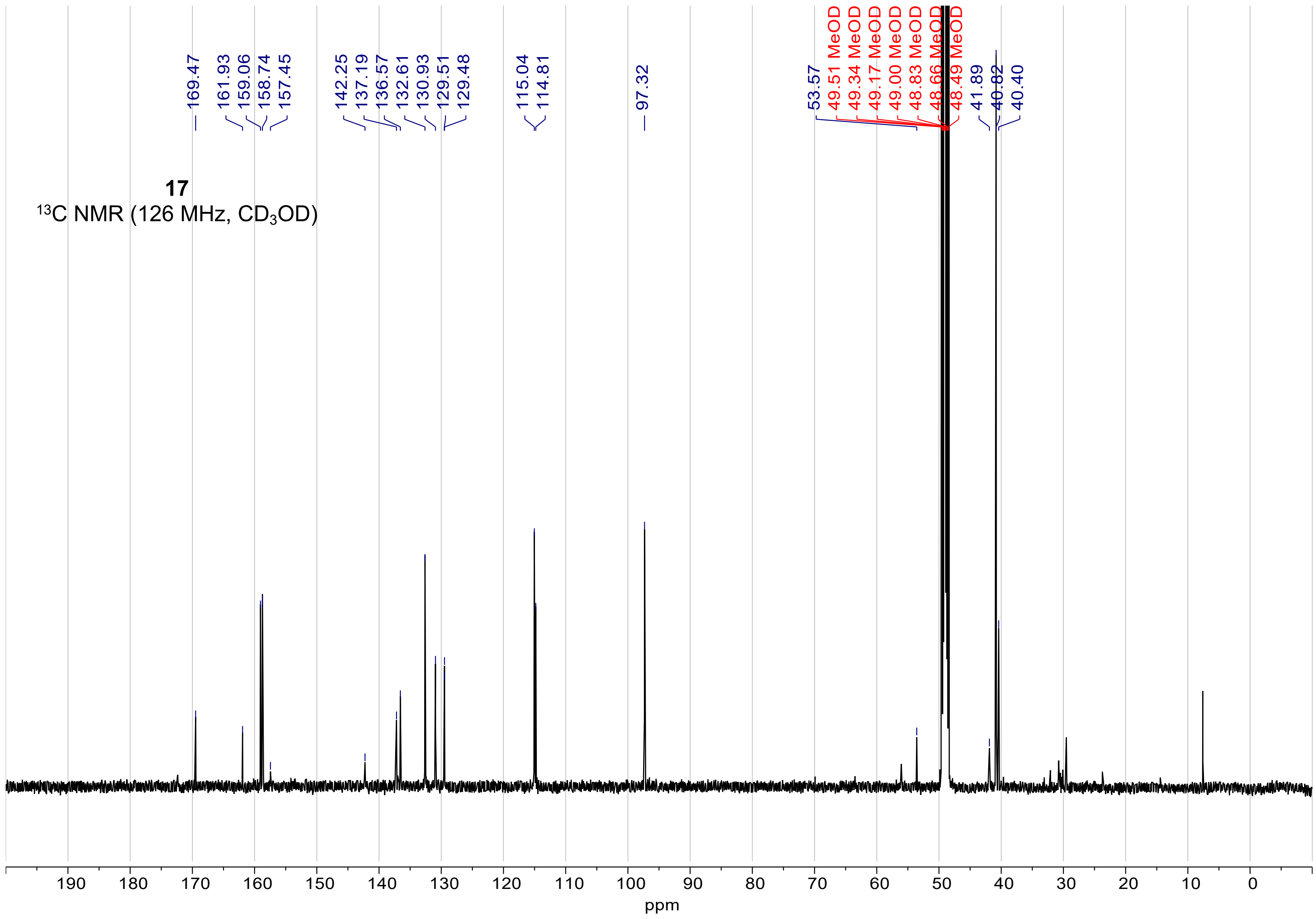
169.47
161.93
159.06
158.74
157.45
142.25
137.19
136.57
132.61
130.93
129.51
129.48
115.04
114.81
97.32
53.57
49.51 MeOD
49.34 MeOD
49.17 MeOD
49.00 MeOD
48.83 MeOD
48.66 MeOD
48.49 MeOD
41.89
40.82
40.40
17
13C NMR (126 MHz, CD3OD)
190
180
170
160
150
140
130
120
110
100
90
80
70
60
50
40
30
20
10
0
ppm

Figure S41. *^{13}C NMR spectrum of 3',6'-bis(dimethylamino)-N-(2-isocyanoethyl)-3-oxo-3H-spiro[isobenzofuran-1,9'-xanthene]-5-carboxamide* **17**.

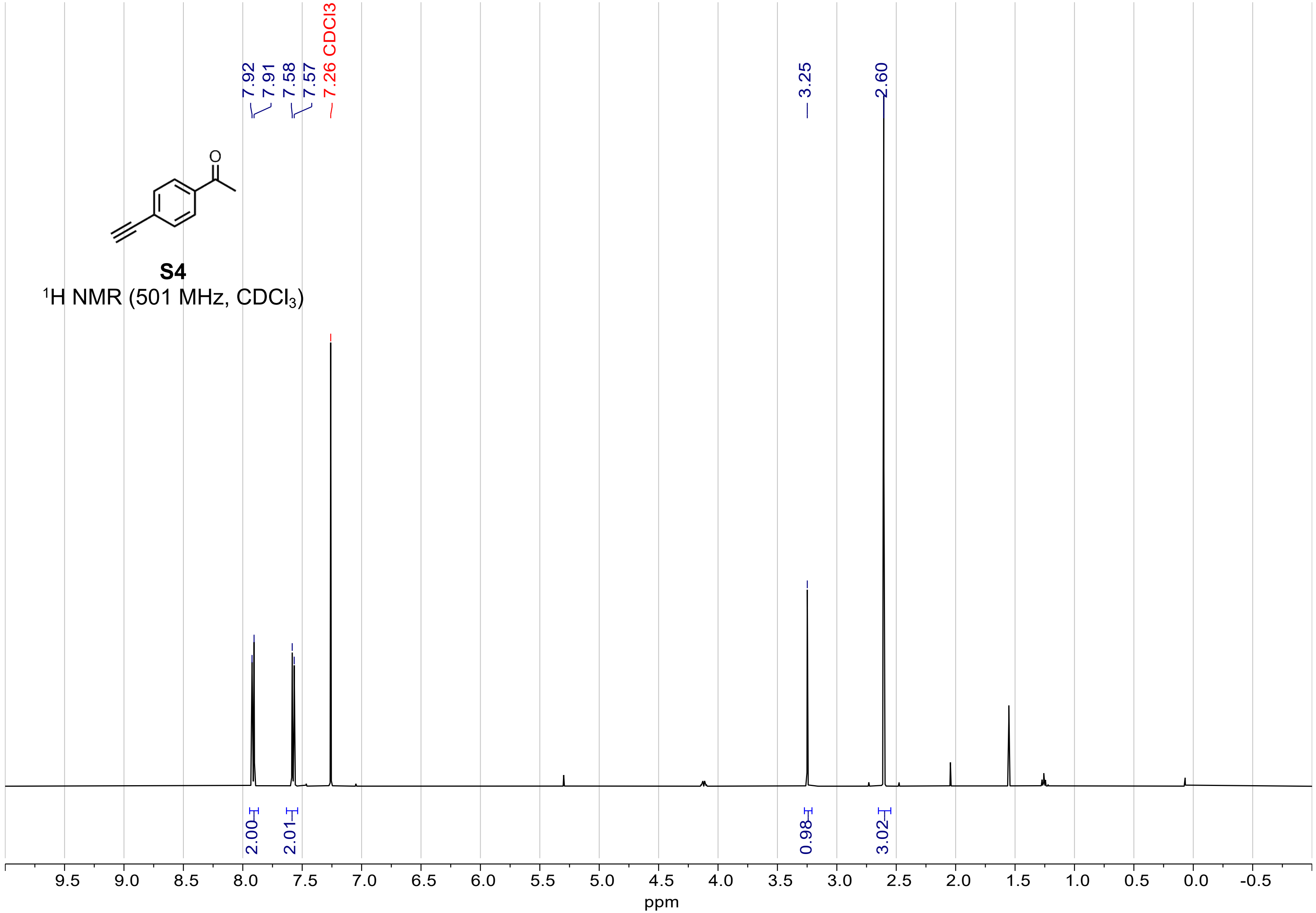

Figure S42. *^{1}H NMR spectrum of 1-(4-ethynylphenyl)ethan-1-one* ***S4****.*

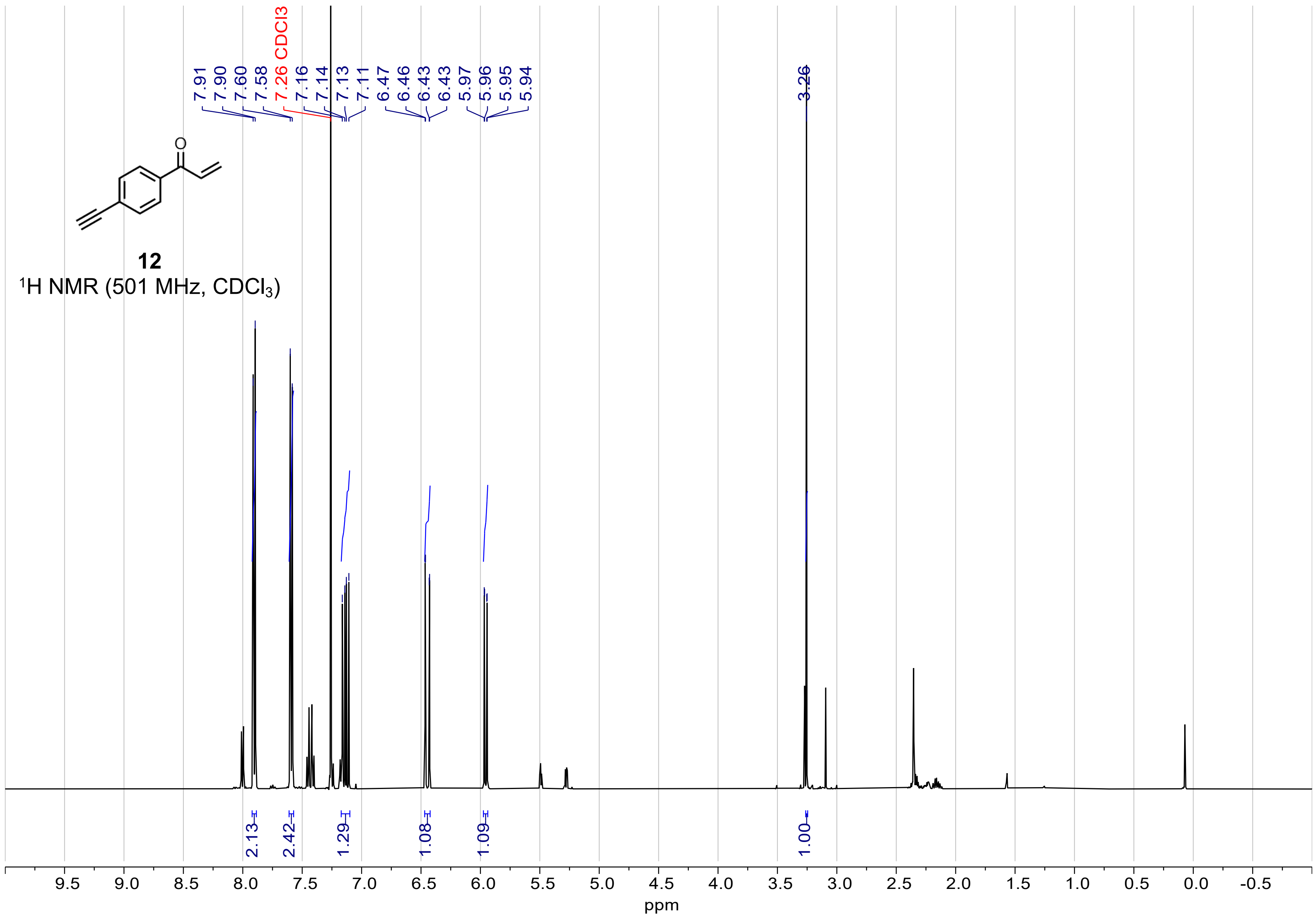

Figure S43. *^{1}H NMR spectrum of 1-(4-ethynylphenyl)prop-2-en-1-one* ***12****.*
